# The Human Phenotype Ontology: Semantic Unification of Common and Rare Disease

**DOI:** 10.1016/j.ajhg.2015.05.020

**Published:** 2015-07-02

**Authors:** Tudor Groza, Sebastian Köhler, Dawid Moldenhauer, Nicole Vasilevsky, Gareth Baynam, Tomasz Zemojtel, Lynn Marie Schriml, Warren Alden Kibbe, Paul N. Schofield, Tim Beck, Drashtti Vasant, Anthony J. Brookes, Andreas Zankl, Nicole L. Washington, Christopher J. Mungall, Suzanna E. Lewis, Melissa A. Haendel, Helen Parkinson, Peter N. Robinson

**Affiliations:** 1 School of Information Technology and Electrical Engineering, University of Queensland, St. Lucia, QLD 4072, Australia; 2 Garvan Institute of Medical Research, Darlinghurst, Sydney, NSW 2010, Australia; 3 Institute for Medical and Human Genetics, Charité-Universitätsmedizin Berlin, Augustenburger Platz 1, 13353 Berlin, Germany; 4 University of Applied Sciences, Wiesenstrasse 14, 35390 Giessen, Germany; 5 Library, Oregon Health & Science University, Portland, OR 97239, USA; 6 School of Paediatrics and Child Health, University of Western Australia, Perth, WA 6840, Australia; 7 Institute for Immunology and Infectious Diseases, Murdoch University, Perth, WA 6150, Australia; 8 Office of Population Health Genomics, Public Health and Clinical Services Division, Department of Health, Perth, WA 6004, Australia; 9 Genetic Services of Western Australia, King Edward Memorial Hospital, Perth, WA 6008, Australia; 10 Telethon Kids Institute, Perth, WA 6008, Australia; 11 Institute of Bioorganic Chemistry, Polish Academy of Sciences, 61-704 Poznań, Poland; 12 Department of Epidemiology and Public Health, School of Medicine, University of Maryland, Baltimore, MD 21201, USA; 13 Institute for Genome Sciences, School of Medicine, University of Maryland, Baltimore, MD 21201, USA; 14 Center for Biomedical Informatics and Information Technology, National Cancer Institute, 9609 Medical Center Drive, Rockville, MD 20850, USA; 15 Department of Physiology, Development and Neuroscience, University of Cambridge, Downing Street, Cambridge CB2 3EG, UK; 16 The Jackson Laboratory, Bar Harbor, ME 04609, USA; 17 Department of Genetics, University of Leicester, Leicester LE1 7RH, UK; 18 European Bioinformatics Institute, European Molecular Biology Laboratory, Wellcome Trust Genome Campus, Hinxton, Cambridge CB10 1SD UK; 19 Academic Department of Medical Genetics, The Children’s Hospital at Westmead, Sydney, NSW 2145, Australia; 20 Discipline of Genetic Medicine, Sydney Medical School, University of Sydney, Sydney, NSW 2145, Australia; 21 Genomics Division, Lawrence Berkeley National Laboratory, 1 Cyclotron Road, Berkeley, CA 94720, USA; 22 Max Planck Institute for Molecular Genetics, Ihnestrasse 63–73, 14195 Berlin, Germany; 23 Berlin Brandenburg Center for Regenerative Therapies, Charité-Universitätsmedizin Berlin, Augustenburger Platz 1, 13353 Berlin, Germany; 24 Institute of Bioinformatics, Department of Mathematics and Computer Science, Freie Universität Berlin, Takustrasse 9, 14195 Berlin, Germany

## Abstract

The Human Phenotype Ontology (HPO) is widely used in the rare disease community for differential diagnostics, phenotype-driven analysis of next-generation sequence-variation data, and translational research, but a comparable resource has not been available for common disease. Here, we have developed a concept-recognition procedure that analyzes the frequencies of HPO disease annotations as identified in over five million PubMed abstracts by employing an iterative procedure to optimize precision and recall of the identified terms. We derived disease models for 3,145 common human diseases comprising a total of 132,006 HPO annotations. The HPO now comprises over 250,000 phenotypic annotations for over 10,000 rare and common diseases and can be used for examining the phenotypic overlap among common diseases that share risk alleles, as well as between Mendelian diseases and common diseases linked by genomic location. The annotations, as well as the HPO itself, are freely available.

## Introduction

The Human Phenotype Ontology (HPO) provides a structured, comprehensive, and well-defined set of over 11,000 classes (terms) that describe phenotypic abnormalities seen in human disease.^1,2^ The HPO has been used for developing algorithms and computational tools for clinical differential diagnostics,^3–5^ for the prioritization of candidate disease-associated genes,^6–11^ in exome sequencing studies,^6–10^ and for diagnostics in clinical exome sequencing.^11^ In addition, the HPO has been used for translational research, including inferring novel drug indications,^12^ characterizing the proteome of the human postsynaptic density,^13^ analyzing Neandertal exomes,^14^ and other topics.^15–22^

The HPO project provides not only a standard phenotype terminology but also a collection of disease-phenotype annotations, i.e., computational assertions that a disease is associated with a given phenotypic abnormality. The HPO currently provides over 116,000 annotations to over 7,000 rare diseases; for instance, the disease Marfan syndrome (MIM: 154700) is annotated with the HPO terms “arachnodactyly” (HP: 0001166), “ectopia lentis” (HP: 0001083), and 46 others. The patterns and specificity of the annotations allow the information content (IC) of each term to be calculated; the IC reflects the clinical specificity of the term and represents a key component of most of the aforementioned algorithms.^23^ Additionally, computational logical definitions are provided for HPO terms. For instance, the HPO term “hypoglycemia” is defined on the basis of “decreased concentration” (PATO: 0001163) in “blood” (UBERON: 0000178) with respect to “glucose” (CHEBI: 17234); this definition uses terms from the ontologies PATO^24^ for describing qualities, UBERON for describing anatomy,^25,26^ and ChEBI for describing small biological molecules.^27^ These definitions are useful for a number of applications, including cross-species phenotype comparisons^6,28,29^ and computational quality control.^30^

The focus of the HPO has, to date, been on rare disease, and correspondingly, it has primarily been adopted by groups from various fields in human genetics, including the Sanger Institute’s Deciphering Developmental Disorders database^22^ and DECIPHER,^31^ the European Cytogeneticists Association Register of Unbalanced Chromosome Aberrations,^32^ the NIH Undiagnosed Diseases Program and Network, the rare-disease section of the UK’s 100,000 Genomes Project, and Genome Canada’s CARE for RARE program, but also by databases for genome-wide association studies (GWASs).^33–35^ Along with rapid technological advances in the field of next-generation sequencing (NGS), personalized medicine is quickly becoming reality,^36^ and initial attempts to use genome sequencing to predict phenotypic abnormalities in common, complex diseases are beginning to show promising results.^37^ In this work, we have extended the range of the HPO from rare to common human disease in order to provide a computational foundation for phenotype-driven analysis of genomes and other translational research in the field of genetics of complex human disease. We have generated over 132,000 phenotypic annotations from the HPO for 3,145 common diseases by using a text-mining approach and have made them freely available to the community. Finally, we demonstrate the uses to which this resource can be put and set out a framework for the future development of the HPO as a community-driven resource for phenotypic analysis of rare and common disease.

## Material and Methods

### Extraction of HPO Terms By Automatic Concept Recognition

Concept recognition (CR) extracts ontology terms from text with the aim of leveraging structured knowledge from unstructured data. For example, CR might be able to identify the term “macrocephaly” (HP: 0000256) within an abstract that contains the phrase “large head” because the latter is listed as a synonym in the entry HP: 0000256. Published CR approaches rely on direct dictionary lookup combined with stemming and word-permutation algorithms^38^ or use natural-language-processing pipelines with techniques such as sentence splitting, tokenization, and part-of-speech tagging.^39^ In our experiments we used a CR tool specifically tailored to address the challenges of extracting phenotype concepts—the Bio-LarK Concept Recognizer.^40^ Bio-LarK uses a two-step approach to index and retrieve ontology terms in combination with a series of language techniques to enable term normalization. In addition to providing standard CR, the system is able to decompose and align conjunctive terms (e.g., “short and broad fingers” aligns to “short finger” [HP: 0009381] and “broad finger” [HP: 0001500]), as well as recognize and process non-canonical phenotypes, such as “fingers are short and broad,” which would be aligned to the same terms as in the previous example. Our current CR approach does not attempt to detect negation, which might represent a cause of false-positive results. However, because of the post-processing steps used to generate the final annotations on the basis of threshold values for annotation frequency and IC (see below), our procedure will not, in general, be sensitive to isolated negative assertions.

### PubMed-MEDLINE 2014 Corpus

The CR process was performed on the 2014 release of the PubMed-MEDLINE corpus. The corpus contains 22,376,811 articles, of which 13,262,617 have a valid title and abstract (most of the missing entries represent articles in languages other than English and only their titles are listed). MEDLINE abstracts are associated with a series of medical subject headings (MeSHs); the main headings (descriptors) provide a schematic description of the topic of the article. The descriptors are divided into 16 categories, including category C, “diseases.” Category C contains 4,620 unique entries, and we refer to it here as “MeSH diseases.”

We note that although MeSH category C is described as comprising diseases, many of the terms in the complete tree C (4,620 entries) do not refer to specific diseases. For instance, many of the terms describe general categories, such as “brain diseases” (MeSH: D001927), veterinary diseases (e.g., “brucellosis, bovine” [MeSH: D002007]), and various other entities, such as “cadaver” (MeSH: D002102). Others represent phenotypic features of diseases rather than actual disease entities; one example is “Cheyne-Stokes respiration” (MeSH: D002639), which is an abnormal breathing pattern that can be observed in diseases such as central sleep apnea syndrome. We excluded such MeSH entries by careful manual curation, leaving a total of 3,145 MeSH category C descriptors that we judged to actually represent specific disease entries. Only these entries were used for the analysis described in this manuscript.

We filtered the 13,262,617 abstracts on the basis of the MeSH terms to retain only those abstracts that included at least one of the 3,145 disease entries from the MeSH disease list and then processed them with the Bio-LarK Concept Recognizer. In some cases, a single abstract was annotated with multiple MeSH disease terms, some of which were also featured as major topics for the article under scrutiny. For the purpose of this analysis, we included all abstracts independently of the number of associated MeSH terms or their major topic feature.

### Filtering HPO Annotations

Many abstracts that describe a given disease also mention a certain HPO term. Consequently, that disease is more likely to be characterized by the corresponding phenotypic abnormality. For instance, the PubMed abstract with the PubMed identifier PMID: 23886833 is indexed with the MeSH term “encephalitis, herpes simplex,” and parsing the record with Bio-LarK reveals a number of HPO terms, including “headache” (HP: 0002315). Therefore, one might be tempted to conclude that this type of encephalitis can be characterized by headaches, but from this single observation it cannot be guaranteed that the abstract is indeed making this assertion. The abstract could, for instance, be describing an adverse effect of a medication, a differential diagnosis, or one of a number of other things. We reasoned that if an HPO term were identified in multiple abstracts associated with a given disease from the MeSH disease list, then it would be more likely to represent a genuine phenotypic abnormality associated with the disease.

However, frequency alone is not a strong enough indicator of a correct association between a phenotype and a disease. Ideally, the phenotype should also be specific to (i.e., present only in a limited number of) certain diseases. Given this required balance, we developed a procedure that aims to distinguish the true annotations on the basis of three metrics: (1) the balance between frequency and specificity; (2) the IC of the term—i.e., the overall degree of specificity of the term in our corpus of diseases; and (3) the disease-category-driven density of a subset of terms, based on the shortest path between them in the HPO. The balance between frequency and specificity is measured with a standard information-retrieval technique: term frequency, inverse document frequency (TFIDF). The TFIDF weighs HPO terms highly if they occur with high frequency among abstracts annotated to a disease but down-weighs terms that are common within the entire corpus (see the following section).

**Figure 1:**
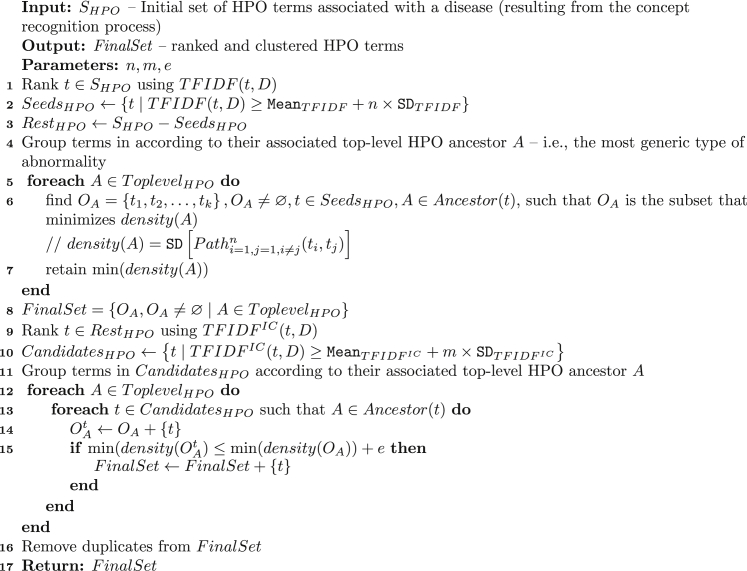
Algorithm 1 Summary of the algorithm used to identify a set of HPO term annotated to diseases. See Material and Methods for explanations.

Figure 1 summarizes the algorithm we have developed. It takes as input the initial set of HPO terms and, using three tuning parameters, produces a final set of candidates. The three tuning parameters control term cutoffs at different stages: (1) *n*, which defines the initial TFIDF threshold used for creating the clustering seeds; (2) *m*, which defines a second specificity threshold (over ${\text{TFIDF}}^{\text{IC}}$; see following section) used for pruning terms left over from the first threshold; and (3) *e*, which defines the density margin that dictates the inclusion or exclusion of a term in a cluster.

The algorithm consists of three steps. First, the initial set of terms is filtered with TFIDF for the creation of clustering seeds (lines 1–3). Second, these clustering seeds are grouped according to their common top-level HPO ancestor —i.e., the top-level HPO abnormality (e.g., blood or skeletal system; line 4). The intuition here is that most diseases affect, in principle, a very limited number of major organs, and hence, most true positives will be grouped according to these major organs (corresponding to the top-level HPO phenotypic abnormality terms). Once the clustering seeds are grouped, we look for the group-based subset of terms that form the single shortest ontological path among them (i.e., the sub-group with the minimum density; lines 5–7). This can be seen as an inverse analogy to the traveling salesman problem, where the shortest path between two terms (i.e., the number of edges required to connect them in the HPO) denotes the cost, and the goal is to minimize the SD of the array of shortest paths. We adapted the Hungarian algorithm to solve this problem. The resulting subset is added to the final list of candidates (line 8). Finally, the list of terms initially filtered out with TFIDF is pruned with ${\text{TFIDF}}^{\text{IC}}$ (lines 9 and 10), and the terms are grouped according to the top-level HPO abnormalities in the same manner as the clustering seeds (line 11). Incrementally, using the group-based density and set of seeds computed in the previous step, we append each leftover term to the seed subset and compute an aggregated density. If the new density is within the limits established by the density margin error parameter (*e*) with respect to the seed density, then the term is added to the final candidates (lines 12–15).

Given a gold-standard corpus, one of the main advantages of this algorithm is the opportunity for learning diverse values for the three parameters, subject to a particular goal. For example, the above-mentioned assumption (i.e., diseases affect a very limited set of major organs) can be transformed into a learning task based on disease categories. We experimented with the 41 manually curated diseases, split into 13 categories dictated by the top-level terms (e.g., cardiovascular diseases, integumentary system diseases, etc.) in the Disease Ontology (DO), and aimed to maximize the category-based true-positive rate. This can be realized by learning sets of parameters corresponding to each disease category. The experimental results showed an overall resulting precision of 66.8%, including highlights such as over 70% precision for diseases by infectious agents (73.0%), diseases of the nervous system (77.8%), or immune system diseases (82.8%). Similarly, we experimented with targeting a maximized overall F-score (i.e., the harmonic mean of precision and recall—a balance between coverage and true-positive rate) and achieved a value of 45.1%. This value is equivalent to an average precision of around 60% associated with a recall of around 40%.

### Information Theoretic Measures for HPO Annotations

The algorithm in Figure 1 uses several information theoretic measures, discussed below.

TFIDF is a standard information-retrieval metric for ranking terms on the basis of their co-occurrence and specificity in the context of a given set of documents. In our case, the goal is to rank HPO terms according to their frequency and specificity in the context of a particular disorder. TFIDF is adapted below (to take into account the disorder-specific context), where *t* denotes an HPO term, *D* denotes the disease under scrutiny, and *T*_*D*_ represents the total number of disorders (i.e., 3,145).

TFIDF(t,D)=TF(t,D)×IDF(t,D)

TF(t,D), the term frequency of HPO term *t* for disease *D*, is defined as the number of *D*-associated abstracts in which a term *t* appears at least once (regardless of the number of mentions in a particular abstract), and the inverse document frequency, IDF(*t, D*), is defined as the logarithm of the quotient of the total number of diseases (*T*_*D*_) divided by the number of diseases for which the HPO term in question is mentioned in at least one abstract.

$$\text{IDF}\left(t,D\right)=\text{log}\frac{T_{D}}{\left|\left\{d\;\in \;D\;:\;t\;\in \;d\right\}\right|}$$

The IC of an individual HPO term within the MEDLINE corpus can be estimated with its frequency among annotations of the entire corpus. Intuitively, the IC of a term such as “fever” (HP: 0001945) is less than that of a term such as “aortic arch calcification” (HP: 0005303) because fewer diseases are characterized by the latter abnormality, and so knowing that an individual has aortic arch calcification narrows down the differential diagnosis much more than knowing that an individual has fever. For each term *t* of the HPO, the IC is quantified as the negative logarithm of its frequency: IC(t)=−logp(t). If a disease is annotated with any term *t* in the HPO, it must also be annotated with all the ancestors of *t*. Therefore, the IC of terms is calculated on the basis of annotations with the term or any of its descendants in the HPO.^41^ For instance, if seven of 1,000 abstracts are annotated with a certain HPO term *t*′, and three more abstracts are annotated with descendants of *t*′, then the frequency of the term would be calculated as *p*(*t*′) = 10 / 1,000, and the IC of the term would be calculated as $\text{IC}{\left(t\right)}^{'}=-\log\;p\left(0.01\right)$. The higher (i.e., closer to the root) in the ontology a term is located, the lower its IC. We use this as an additional term to define ${\text{TFIDF}}^{\text{IC}}$ for HPO term *t* and disease *D* as

$${\text{TFIDF}}^{\text{IC}}\left(t,D\right)=\text{TFIDF}\left(t,\;D\right)\times \text{IC}\left(t\right).$$

### Calculation of Phenotypic Overlap with an Extended Jaccard Index

The Jaccard index is a standard measure of similarity between two sample sets, *A* and *B*, and is defined as the size of the intersection divided by the size of the union of the sample sets:

$$J\left(A,B\right)=\frac{\left|A\cap B\right|}{\left|A\cup B\right|}.$$

The value of the Jaccard index ranges from 0 for complete dissimilarity to 1 for identity. In a typical set-based context, the Jaccard index is computed on the strict intersection and union of the elements. However, in our context these elements represent ontology terms, structured in a logical hierarchy. And, as such, we can rely on the subsumption relation between terms when computing intersection and union. We exploited this aspect in the computation of the Jaccard index. A match between two terms was recorded not only when the two terms matched exactly (i.e., “cranial hyperostosis” is the same as “cranial hyperostosis”) but also when the subsumption relation was present (i.e., “cranial hyperostosis” is a parent of “calvarial hyperostosis” and an ancestor of “mandibular hyperostosis”; Figure S1).

### Validation of HPO Annotations for Common Disorders

We chose three to five common diseases from each of the 13 DO upper-level categories used in our common-disease network (CDN; see below) for a total of 41 diseases. We used a Perl script to choose diseases at random from among all diseases in the categories. We examined the diseases manually by assessing each HPO term mentioned at least once in any abstract describing the disease in question (thus, we evaluated substantially more HPO terms than merely the set of terms chosen by our annotation pipeline on the basis of frequency and specificity of the term). Biocuration was performed by N.V., G.B., D.V., A.Z., M.H., and P.N.R., and all annotations were validated by P.N.R., who is both a computer scientist and a medical doctor. This allowed us to assess the true-positive, false-positive, and false-negative rates as shown in Tables S1–S41.

### CDN

In order to validate and visualize the phenotype annotations obtained for common disease, we constructed a CDN by computing the pairwise similarity of a total of 1,678 diseases (i.e., annotated MeSH entries) belonging to 13 DO categories such as “nervous system disease” (DOID: 863) or “respiratory system disease” (DOID: 1579) (Figure S2). Note that some diseases belong to multiple DO classes (Figure S3).

For each disease, we obtained all the HPO annotations that our CR algorithm had associated with the disease. The annotation frequency of a term was defined as the proportion of diseases that were annotated by the term or any of its descendent terms. In order to calculate similarity between two terms $\left(t_{1},t_{2}\right)$, we used the IC of their most informative common ancestor (MICA),^3^ denoted as $\text{MICA}\left(t_{1},t_{2}\right).$

We used the above-mentioned term-similarity measures to calculate a semantic-similarity score for two diseases $\left(D_{1},D_{2}\right)$. In our case, for each of the terms of $D_{1}$, the “best match” among the terms annotated $D_{2}$ was found, and the average overall query terms was calculated. This was defined as the similarity:

$$\text{sim}\left(D_{1}\to D_{2}\right)=\text{avg}\left[\sum_{s\in D_{1}}\max_{t\in D_{2}}\;\text{IC}\left(\text{MICA}\left(s,t\right)\right)\right],$$

where the average was taken over all terms *s* to which disease $D_{1}$ is annotated. Note that this score is asymmetric, i.e., it is not necessarily the case that $\text{sim}\left(D_{1}\to D_{2}\right)=\text{sim}\left(D_{2}\to D_{1}\right)$. Therefore, for the analysis described here, we used a symmetric similarity score:

$$\text{sim}\left(D_{1},D_{2}\right)=\frac{1}{2}\text{sim}\left(D_{1}\to D_{2}\right)+\frac{1}{2}\text{sim}\left(D_{2}\to D_{1}\right).$$

The CDN consists of nodes that represent common diseases and edges that indicate that two diseases are phenotypically similar. In order to create the CDN, we calculated the symmetric similarity score for all pairs of diseases. The network was visualized with the force-directed layout algorithm of Cytoscape,^42^ whereby an edge between nodes was drawn if the similarity between two corresponding diseases exceeded 2.0 (simulation cutoff [simcut]). The final CDN (CDN-o) consisted of 1,148 diseases and 4,059 edges.

### Statistical Significance of the CDN

In order to test the statistical significance of the distribution of phenotypic similarity among diseases within the same disease category or between different categories, we introduced the concept of the gray-edge fraction (GEF). That is, we visualized edges between nodes (diseases) that do not belong to one of the same 13 general disease categories as gray edges. The GEF was defined as the proportion of gray edges among all edges in the CDN. The lower the GEF, the better the phenotypic clustering of diseases agrees with the classification of the diseases into the 13 categories. The original CDN (CDN-o) comprised 3,547 edges, 998 of which were gray edges, corresponding to a GEF of 0.246 (red arrow in Figure S4A). We tested two randomization procedures, edge randomization (er) and annotation randomization (ar).

The edge-permutation procedure retains the number of edges and the degree distribution of the network.^43^ Two edges, A-B and X-Y, are chosen at random and reshuffled to create the edges A-Y and X-B. Reshuffling is skipped if the edges A-Y and X-B already exist. Reshuffling is performed 10,000 times, resulting is an edge-randomized version of CDN-o, which we call CDN-er and for which we can again compute the GEF. We constructed 1,000 versions of CDN-er and plotted the distribution of the resulting GEF values in Figure S4A. As one can see, the p value of the CDN is less than 0.001 because none of the edge-randomized CDNs achieved the same or a smaller GEF than the original CDN.

We additionally performed a test in which we randomized the HPO terms associated with each disease (ar). For this, we randomly selected 50% of the terms associated with each disease and replaced them with randomly selected HPO terms. We computed the randomized CDN (called CDN-ar) by using the above procedures used to construct the CDN-o. We repeated this procedure 100 times and computed the GEF for each CDN-ar. Note that each CDN-ar might not have the same amount of nodes and edges as the CDN-o. When using the same simcut (2.0) used for constructing the CDN-o, we obtained much smaller networks (fewer than 100 nodes). The distribution of GEF values of CDN-ar with simcut 2.0 is shown in Figure S4B. No CDN-ar achieved a GEF less than or equal to the CDN-o GEF, which corresponds to a p value of less than 0.01. We modified the simcut to 1.4 because it leads to CDN-ar versions with approximately the same amount of nodes as CDN-o. The distribution of the resulting GEF values is shown in Figure S4C. Again, not a single CDN-ar constructed with a simcut of 1.4 achieved a GEF less than or equal to the CDN-o GEF, which corresponds to a p value of less than 0.01.

### GWAS Data

GWAS Central provides a comprehensive collection of summary-level genetic-association data and advanced visualization tools to allow comparison and discovery of datasets from the perspective of genes, genome regions, phenotypes, or traits.^33^ The project collates association data and study metadata from many disparate sources, including the National Human Genome Research Institute GWAS Catalog,^35^ and receives frequent data submissions from researchers who wish to make their research findings publicly available. All gathered and submitted data are extensively curated by a team of post-doctoral genetics researchers who manually evaluate each study for its range of phenotype content and apply appropriately chosen MeSH terms. As of December 2014, the resource contained 69 million p values for over 1,800 studies.

Data and metadata for up to 1,000 associations can be freely downloaded from the BioMart-based system (GWAS Mart), and larger custom data dumps (up to and including the complete database) are available via contacting the GWAS Central development team and agreeing with a data-sharing statement. Thus, to provide data for the present study, we generated a tab-separated file representing 1,574 studies and 34,252 unique SNPs (annotated to 675 unique MeSH terms) and containing the GWAS Central study identifier, PubMed identifier, dbSNP “rs” identifier, p value, and MeSH identifier for all associations with p < 1 × 10^−5^. We compiled the list of genes considered for our experiments by retrieving the “mapped genes” column from the database SCAN and identifying those genes corresponding to the GWAS Central SNPs. Where no mapped genes were reported, we used the upstream, as well as downstream, genes listed by SCAN.^44^

## Results

### Generation of Phenotype Annotations for Common Disease by CR

**Figure 2:**
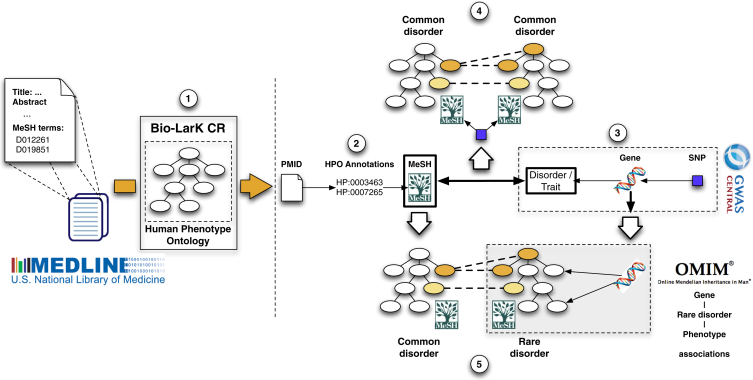
Overview of CR and Bioinformatic Analysis The analysis was performed in several major steps. (1) Bio-LarK was used to analyze the PubMed-MEDLINE 2014 corpus, which resulted in a total of 5,136,645 abstracts annotated with MeSH terms and phenotypic features. (2) For each of 3,145 resulting diseases, the frequency and specificity of HPO terms found in the abstract were used for inferring phenotypic annotations. (3) These annotations were used for producing disease models for each of the diseases. (4) Medical validation of the annotations was performed on the basis of disease, phenotype, and SNP annotations in GWAS Central for phenotype sharing in common disease. (5) Validation with OMIM, Orphanet, and DO was used for assessing phenotype sharing between rare and common diseases linked to the same locus.

We applied a phenotype-aware CR system (the Bio-LarK Concept Recognizer^40^) to all available abstracts in PubMed in order to extract phenotypic annotations for common diseases. We first retrieved the MeSH terms associated with PubMed abstracts and used them to retain only those abstracts focused on diseases. 5,136,645 of 22,376,811 articles listed in PubMed had an abstract and could be assigned to such a MeSH disease term (see Material and Methods for a description of our inclusion criteria for MeSH disease entries; a total of 3,145 diseases were included). Second, we applied CR on the resulting set, after which a total of 930,805 HPO annotations were assigned to 3,145 common diseases. Finally, we filtered this initial set of HPO terms, by using a ranking-and-clustering method with the aim of maximizing the F-score computed on a manually curated gold-standard set of 41 common diseases (see Material and Methods). This approach aims to maximize the text-mining accuracy, defined as the harmonic mean of the precision and recall of the derived annotations. This final set comprised 132,006 HPO annotations covering 4,459 unique HPO terms. The mean number of annotations per disease was 41.97 (range, 1–271; median, 32) and consisted of terms belonging to all of the top-level HPO categories (Figure S5). Figure 2 provides an overview of the analysis procedures used to generate and validate the common-disease annotations.

As an example, Table S1 lists the annotations produced for “giant cell arteritis” (MeSH: D013700), which includes terms such as “vasculitis” (HP: 0002633), “granulomatosis” (HP: 0002955), and “amaurosis fugax” (HP: 0100576). The annotations are highly accurate, although some nuances are not detected by the CR process. For instance, “facial palsy” (HP: 0010628) and “renal amyloidosis” (HP: 0001917) are classic manifestations of giant cell arteritis. The list of phenotypic manifestations is by no means complete, given that it failed to identify manifestations such as “dysphagia” (HP: 0002015), “trismus” (HP: 0000211), and “encephalopathy” (HP: 0001298). Nonetheless, the CR process was able to capture a largely accurate subset of phenotypic abnormalities for giant cell arteritis, such that 64% of the annotations were true positives.

We estimated the overall quality of the HPO annotations by inspecting the automatically extracted annotations for a set of 41 common diseases randomly chosen from 13 upper-level DO^45^ categories that had a MeSH disease identifier and thus could be analyzed analogously to the common MeSH diseases. The process involved manually validating of all HPO annotations extracted by the CR process and comparing them to the results of detailed manual curation for the estimation of the true- and false-positive and the false-negative rates. We note that it is not informative to calculate a true-negative rate across the entire HPO because even if the CR process flags several hundred terms, the great majority of the over 10,000 HPO terms will be true negatives. We found that maximizing the overall F-score (i.e., the harmonic mean of precision and recall) led to a mean F-score of 45.1% (i.e., a mean precision of around 60% accompanied by a mean recall of around 40%). In separate experiments, we found that a CR run with parameters designed to maximize the precision in each of the 13 categories achieved a mean precision of 66.8% (data not shown). However, we chose to use the annotations derived from the F-score procedure for the remainder of the analysis. The complete set of annotations associated with the 41 common diseases, including flags for true positives, false positives, and false negatives, can be found in Tables S1–S41.

### A Common-Disease Phenotypic Network

**Figure 3:**
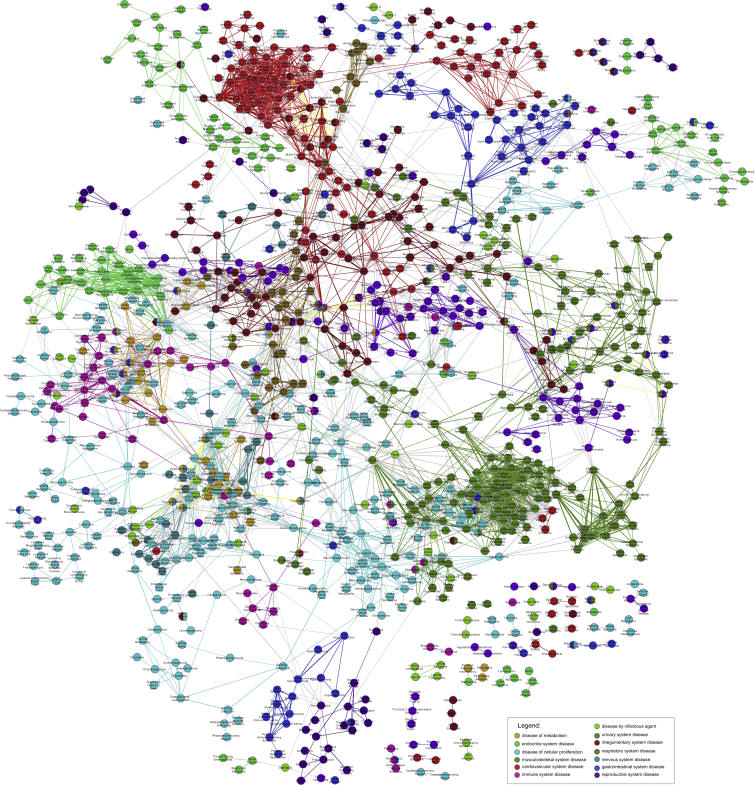
Phenotypic Network of Common Disease A total of 1,678 common diseases could be mapped to at least one of 13 top-level DO categories (Figures S5 and S6). 1,148 of these diseases displayed a connection to another disease with a phenotypic similarity score of at least 2.0. They are shown as a node in the graph and are colored according to membership in the upper-level disease categories. The thickness of the connections between the nodes reflects the degree of phenotypic similarity

As a first test of the medical validity of the HPO annotations for common-disease phenotypes, we visualized the network of phenotypic similarity of a subset of 1,678 diseases, such as “nervous system disease” (DOID: 863) or “respiratory system disease” (DOID: 1579), belonging to 13 DO categories. 1,148 of the 1,678 diseases showed at least one connection to another disease (phenotypic similarity score above a threshold of 2.0), and thus the final CDN comprised 1,148 diseases. Phenotypic relationships between these diseases are shown by the linking of all pairs of diseases exceeding the threshold similarity score (Figure 3). Although generated independently of the disorder classes, the resulting phenotypic network clearly displays clusters corresponding to the disease categories.

We then constructed randomized phenotypic networks as described in the Material and Methods and calculated the number of links between diseases from the same disease category. We found that the observed correlation between network connections and disease class is highly significant (Figure S4). Thus, the phenotypic network of common diseases, as defined by the HPO, is made up of dense clusters of shared phenotypic features that show characteristic patterns of interconnections between selected areas of the phenotypic continuum, just as we had previously observed for Mendelian diseases.^2^ The high correlation between the computationally created network clusters and the manually curated disease classifications provides further evidence that the automatically created annotations are clinically meaningful and provide a largely correct description of the disease in question.

### Phenotypic and Genetic Overlap across Complex Diseases

GWASs have been performed for a wide range of common diseases and traits, and over 6,000 strong SNP associations $\left(\text{p}<10^{-8}\right)$ have been identified.^35^ Variation at multiple genetic loci collectively influences the likelihood of developing many common and complex diseases; for instance, it is estimated that that about 8,300 independent and predominantly common SNPs contribute to risk for schizophrenia^46^ (MIM: 181500). Although the genetic architecture is likely to differ for different diseases, often the trait architecture consists of a few loci of relatively large effect and many additional loci that have a very small effect on phenotype.^47^ To understand the genetics of complex disease, it is important to consider the phenotypic and genetic overlap among diseases. For instance, susceptibility loci that are common to both multiple ulcerative colitis and Crohn disease have been identified by GWASs, and some of these loci are even shared with several other autoimmune disorders.^48^ Similarly, several psychiatric disorders share risk loci.^49^ The study of the distribution of overlapping loci within a group of diseases might suggest shared pathways and common pathogenetic features.^23^ On the other hand, the lack of overlap of other loci could help to identify pathogenic mechanisms that are unique to specific diseases and could help to explain phenotypic diversity across the spectrum of diseases in fields such as autoimmunity or psychiatry.^50^ The computational resources presented here offer a tool for comprehensively measuring the phenotypic overlap of a wide range of common diseases that share risk loci.

From the total of 16,152 unique SNPs, 863 were associated with more than one disorder, and the total number of unique disorders was 300. 673 SNPs were associated with two disorders, 130 were associated with three, and 60 were associated with more than four (Figure S6). 577 of these SNPs were associated with a total of 79 unique diseases in our corpus and were used for the following analysis.

**Figure 4:**
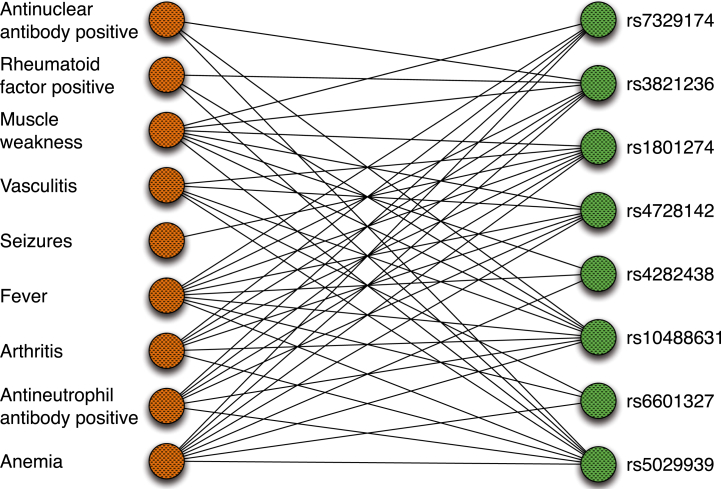
Phenotype-SNP Network For constructing this network, individual HPO terms were connected to SNPs if the SNP was significantly associated with a disease characterized by the HPO term in question. For instance, the SNP rs5029939 is significantly associated with both Sjögren syndrome^51^ and systemic lupus erythematosus.^52^ The diseases also share a number of phenotypic features, including “antinuclear antibody positivity” (HP: 0003493) and “xerostomia” (HP: 0000217). A small and particularly dense subset of the network was manually chosen. The network is centered on ten HPO terms representing clinical features that are common in autoimmune diseases.

The mean Jaccard index for the pairwise comparison on the 577 SNPs was 0.251±0.132. That is, for each pair of SNPs, the phenotypic annotations of the corresponding diseases were compared to each other with the extended Jaccard index (Figure S1). Randomly chosen disease comparisons from the existing pool of MeSH diseases displayed a significantly lower overlap of 0.130 ± 0.094  ( $\text{p}=2.29\times 10^{-57}$, paired t test). Our results show a pervasive phenotypic sharing among complex diseases that are also associated with the same SNP. As an example, we show an excerpt of the phenotype-SNP network centered on autoimmune phenotypes. Ten phenotypic abnormalities observed in persons with these diseases are shown together with SNPs associated with one or more diseases displaying these features, such as Sjögren syndrome (MIM: 270150) and systemic lupus erythematosus (MIM: 152700). It can be seen that there is a dense interconnected network of phenotypes and SNPs (Figure 4). These results extend recent findings concerning a human disease-symptom network based on 322 individual symptoms extracted from MeSH.^53^ We provide a CDN browser that allows users to navigate through the network of common diseases that are interconnected by phenotypic similarity (Figure S7).

### Phenotypic and Genetic Overlap across Complex and Mendelian Diseases

Numerous, highly penetrant mutations in individual genes have been identified in thousands of Mendelian diseases. Common variants associated with complex diseases are enriched in genes mutated in Mendelian diseases.^54^ For instance, certain mutations in presenilin 1 (*PSEN1*) cosegregate with early-onset familial Alzheimer disease^55^ (MIM: 607822), whereas variants in the *PSEN1* promoter are associated with increased risk for complex (non-Mendelian) Alzheimer disease.^56^ Similarly, common polymorphisms associated with blood lipoprotein concentrations are often located in the genomic vicinity of genes associated with Mendelian disorders of lipoprotein metabolism, such as *ABCG8*, *LCAT*, *APOB*, *LDLR*, *PCSK9*, *CETP*, *LPL*, *LIPC*, and *ABCA1*.^57,58^ We therefore reasoned that the phenotypic-genetic overlap might be a general tendency for rare and common diseases located at the same genetic locus. As per the method described above, we examined 485 genes shared between the complex- (GWAS) and rare-disease datasets. GWAS SNPs were previously mapped to genes with SCAN.^44^ In a manner similar to that used in the common-disease-phenotype experiment, we then measured the phenotypic overlap between the complex diseases from GWAS Central^33^ and rare, Mendelian diseases associated with the genes in question. The overlap measure used in the experiments was the Jaccard index and was computed in the same manner as in the case of the complex-disease overlap. This resulted in a mean value of 0.027±0.032, which was higher than the corresponding value for randomized pairs of common and rare disease (same procedure as above), 0.021 ± 0.023 ( $\text{p}=1.6\times 10^{-7}$, paired t test). Table 1 shows some examples of GWAS hits that are linked to genes in which mutations cause Mendelian diseases with phenotypic overlap.

## Discussion

Translational research in Mendelian diseases has benefited enormously from databases of the phenotypic features associated with individual diseases, such as OMIM,^65^ Orphanet,^66^ and more recently the HPO.^1,2^ Analysis of such data has led to the idea that diseases that display similar phenotypic features are caused by mutations in functionally related genes. For instance, genetically heterogeneous diseases such as Fanconi anemia, Bardet-Biedl syndrome, or Usher syndrome are related to mutations in genes of a single biological module. Such modules can be a multiprotein complex, a pathway, or a single cellular or subcellular organelle.^67–70^ To date, however, it has been difficult to perform analogous research on complex-disease phenotypes because resources to carry out comparable analyses have been lacking.

GWASs emerged in the first decade of the new millennium as a powerful tool for elucidating the genetic architecture of common disease.^33,35^ The advent of clinical whole-genome sequencing^71^ (WGS) is promising to lead to personalized genomic medicine. It is becoming apparent that precise phenotype analysis can substantially improve the ability to interpret the results of NGS. In rare diseases, for instance, diagnostic NGS yields plausible candidate variants in several genes, and making diagnoses will require that the consequences of these variants be analyzed and integrated with clinical findings.^72^ In fact, using the HPO to analyze phenotypic data has been shown by multiple groups to improve the ability of NGS-based methods to identify candidate disease-associated genes and make clinical diagnoses.^5–11,21^ These methods have been tested on exomes and large NGS gene panels. In contrast, WGS provides a nearly comprehensive view on non-coding variations, a class of variation that makes up the majority of known risk factors for common disease.^35^ WGS currently cannot be used reliably for the prediction of common disease in a clinical diagnostic setting.^73^ However, this is increasingly becoming a topic of bioinformatics research^37,74,75^ and is likely to increase in importance as large-scale efforts such as the UK’s 100,000 Genomes Project begin to produce and interpret data. We speculate that phenotype analysis will be just as beneficial to WGS-based diagnostics of common disease as it has been shown to be for rare disease.^5–11,76,77^ One area of particular interest stems from the observation that genes harboring common variants associated with a common disease might also carry large-effect mutations in a subset of individuals at the extremes of the trait. For instance, the polymorphism rs6817105, which is located about 167,000 nt upstream of *PITX2*, was found to be associated with atrial fibrillation.^78^ More recently, a de novo nucleotide substitution in the promoter region of *PITX2* (319 nucleotides upstream of the transcription start site) was identified in an individual with severe atrial fibrillation.^79^ Observations such as this and those summarized in Table 1 suggest that rare-disease phenotypes will be extremely useful in evaluating the findings of WGS performed on individuals with common, complex diseases and underline the utility of annotating rare and common diseases with a common phenotype ontology.

To generate the resource, we developed a statistical framework to evaluate the pattern of co-occurrences of HPO terms (phenotypic features) and diseases in PubMed abstracts. Previous efforts in the field of clinical text mining have shown the enormous promise of data extraction from articles or electronic health records (EHRs) for translational research; one of the keys to tapping this resource lies in the ability to reliably extract clinical information from the EHRs by text mining and other methods.^80^ For instance, phenome-wide association scans (PheWASs) search EHRs for disease-gene associations by using the International Classification of Disease (ICD9) billing codes, which are available in most EHR systems, and have been shown to be able to replicate findings of traditional GWASs and identify novel associations.^81,82^ Other groups have used EHR data to detect adverse medication interactions.^83^ The project presented here had different goals, in that we developed a statistical model to infer the spectrum of phenotypic abnormalities that characterize diseases rather than to classify individuals’ records according to whether a certain disease was present or not (as has been the case for the majority of the PheWASs and similar studies published to date; we note that many of these studies utilized the word “phenotype” to refer to a disease entity, whereas our study has examined the individual phenotypic features of diseases).

The algorithms we developed to derive disease models from the annotation patterns of PubMed abstracts combined a number of components, including (1) semantic CR (Bio-LarK^40^); (2) an adaptation of the TFIDF method, whereby diseases take the place of documents, and the “document frequency” of individual HPO terms is calculated from the number of abstracts containing the term; (3) an evaluation of the IC of individual HPO terms for calculating the semantic similarity^84,85^ between terms; and (4) a heuristic graph clustering method that attempts to extend seed terms with particularly high TFIDF values to create a dense phenotypic network. This allowed us to develop annotations for over 3,000 common, complex diseases, and we demonstrated the potential utility of the resource by an analysis of phenotypic overlap between common and rare disease, as well as between complex diseases that share one or more genetic associations. The platform we have made available, together with the data, is in itself a valuable resource for the community. In addition to providing a way to download the data in a tab-separated form, or to access it programmatically via application programming interfaces, the website also enables a phenotype- and disorder-centric browsing of MEDLINE abstracts and browsing within the CDN (Figure S7). This resource could be useful for physicians who are caring for persons with a given disease and who present with a particular manifestation or complication of that disease (denoted by an HPO term). The browser will present all PubMed abstracts that were identified in our study and that describe both the disease and the phenotypic manifestation, which might provide information that could be helpful in clinical management.

There are several limitations of the common-disease annotations that we have presented here. First and foremost, the annotations were derived by a computational CR (text-mining) process and contain both false-positive and false-negative annotations. The HPO project, which is being developed as a part of the Monarch Initiative, will be actively revising and expanding the annotations and developing new areas of the ontology itself as needed for the analysis of common disease, much as it has been doing in the field of rare diseases since 2007.^1,2^ Several characteristics of particular importance to common diseases, such as the past medical history and the time course of disease, are not currently well captured by the computational data structures and algorithms that have been developed for rare disease and will need to be established in future work. The results of the analysis of phenotypic overlaps are highly statistically significant but do not provide proof of a common pathophysiological basis of the diseases involved. However, we contend that the results we have presented in this manuscript demonstrate that the common-disease HPO annotations can be used for the computational analysis of phenotypic abnormalities across a previously unheard-of range of rare and common diseases, including over 7,000 rare diseases and 3,145 common diseases. To the best of our knowledge, there is no comparable computational resource that provides both an extensive phenotype ontology and annotations to over 10,000 diseases, as well as an algorithmic basis for calculating the similarity between arbitrary sets of phenotypic abnormalities and specific diseases^3^ and a foundation for translational research on topics such as cross-species phenotype mapping.^6,23^

The HPO project has been under development since 2007 and has mainly focused on rare and primarily Mendelian diseases.^1,2^ The work presented here provides users with over 132,000 phenotypic annotations for 3,145 common diseases derived via text mining. It is hoped that these annotations, as well as the underlying HPO terms, will be useful for both clinicians and researchers. Future work will include biocuration efforts to validate and extend the current set of annotations, to add metadata such as the age of onset, severity, clinical course, and response to treatments, and to extend the HPO to provide an even broader range of terms for the manifestations of complex disease, with the intention of providing a comprehensive resource for translational bioinformatics across the entire spectrum of human disease.

## Figures and Tables

**Table 1:** Phenotypic Overlap between Rare and Complex Disorders

**Gene: Associated Rare Disease**	**Reference SNP: Complex Disease**	**Common HPO Terms**
*CD247*: immunodeficiency due to defect in CD3-ζ (MIM: 610163)	rs840016: rheumatoid arthritis^59^	edema (HP: 0000969), arthralgia (HP: 0002829), arthritis (HP: 0001369), autoimmunity (HP: 0002960)
*FSHR*: ovarian hyperstimulation syndrome (MIM: 608115) and ovarian dysgenesis 1 (MIM: 233300)	rs2268361: polycystic ovary syndrome^60^	abnormality of the ovary (HP: 0000137), decreased fertility (HP: 0000144), primary amenorrhea (HP: 0000786)
*PPARG*: lipodystrophy, familial partial, type 3 (MIM: 604367)	rs13081389: type 2 diabetes mellitus^61^	hyperglycemia (HP: 0003074), hyperinsulinemia (HP: 0000842), hypertension (HP: 0000822)
*LPL*: type I hyperlipoproteinemia (MIM: 238600)	rs295: metabolic syndrome X^62^	hypercholesterolemia (HP: 0003124), hyperlipoproteinemia (HP: 0010980), coronary artery disease (HP: 0001677), pancreatitis (HP: 0001733)
*LRRK2*: Parkinson disease 8 (MIM: 607060)	rs34778348: Parkinson disease^63^	rigidity (HP: 0002063), bradykinesia (HP: 0002067), dementia (HP: 0000726), resting tremor (HP: 0002322)
*HCN4*: sick sinus syndrome 2 (MIM: 163800)	rs7164883: atrial fibrillation	arrhythmia (HP: 0011675), tachycardia (HP: 0001649), sinus brachycardia (HP: 0001688)
*HYDIN*: ciliary dyskinesia, primary, 5 (MIM: 608647)	rs12149070: COPD^64^	respiratory tract infection (HP: 0011947), respiratory insufficiency (HP: 0002093), bronchiectasis (HP: 0011947)

GWAS hits localized in the vicinity of Mendelian-disease-associated genes could be associated with common diseases that have phenotypic overlaps with the corresponding Mendelian diseases. Seven examples in which common and rare diseases linked to neighboring loci and showed substantial phenotypic overlap were manually chosen. The protein-coding gene associated with the rare disease, as well as the accession number of the polymorphism located in non-coding sequence near the gene, is shown. The following abbreviation is used: COPD, chronic obstructive pulmonary disease.

## Web Resources

The URLs for data presented herein are as follows:

Bio-LarK, http://bio-lark.org/
Common Disease Phenotype Browser, http://pubmed-browser.human-phenotype-ontology.org/
GWAS Central, http://help.gwascentral.org/info/data/data-sharing-statement/
Human Phenotype Ontology, http://www.human-phenotype-ontology.org/
Monarch Initiative, http://monarchinitiative.org
OMIM, http://omim.org/

## Supplemental Data

Document S1. Figures S1–S7 and Tables S1–S41

Document S2. Article plus Supplemental Data
