# Supplementary material for: The Human Phenotype Ontology: Semantic Unification of Common and Rare Disease
Source: Am J Hum Genet. 2015 Jul 2;97(1):111–24. doi: 10.1016/j.ajhg.2015.05.020 (PMC4572507; doi:10.1016/j.ajhg.2015.05.020)
Supplement: Document S2. Article plus Supplemental Data [file mmc2.pdf]

The Human Phenotype Ontology: Semantic Unification of Common and Rare Disease

Tudor Groza,[1,2,25] Sebastian Köhler,[3,25] Dawid Moldenhauer,[3,4] Nicole Vasilevsky,[5] Gareth Baynam,[6,7,8,9,10] Tomasz Zemojtel,[3,11] Lynn Marie Schriml,[12,13] Warren Alden Kibbe,[14] Paul N. Schofield,[15,16] Tim Beck,[17] Drashtti Vasant,[18] Anthony J. Brookes,[17] Andreas Zankl,[2,19,20] Nicole L. Washington,[21] Christopher J. Mungall,[21] Suzanna E. Lewis,[21] Melissa A. Haendel,[5] Helen Parkinson,[18] and Peter N. Robinson[3,22,23,24,*]

The Human Phenotype Ontology (HPO) is widely used in the rare disease community for differential diagnostics, phenotype-driven analysis of next-generation sequence-variation data, and translational research, but a comparable resource has not been available for common disease. Here, we have developed a concept-recognition procedure that analyzes the frequencies of HPO disease annotations as identified in over five million PubMed abstracts by employing an iterative procedure to optimize precision and recall of the identified terms. We derived disease models for 3,145 common human diseases comprising a total of 132,006 HPO annotations. The HPO now comprises over 250,000 phenotypic annotations for over 10,000 rare and common diseases and can be used for examining the phenotypic overlap among common diseases that share risk alleles, as well as between Mendelian diseases and common diseases linked by genomic location. The annotations, as well as the HPO itself, are freely available.

Introduction

The Human Phenotype Ontology (HPO) provides a structured, comprehensive, and well-defined set of over 11,000 classes (terms) that describe phenotypic abnormalities seen in human disease.[1,2] The HPO has been used for developing algorithms and computational tools for clinical differential diagnostics,[3–5] for the prioritization of candidate disease-associated genes,[6–11] in exome sequencing studies,[6–10] and for diagnostics in clinical exome sequencing.[11] In addition, the HPO has been used for translational research, including inferring novel drug indications,[12] characterizing the proteome of the human postsynaptic density,[13] analyzing Neandertal exomes,[14] and other topics.[15–22]

The HPO project provides not only a standard phenotype terminology but also a collection of disease-phenotype annotations, i.e., computational assertions that a disease is associated with a given phenotypic abnormality. The HPO currently provides over 116,000 annotations to over 7,000 rare diseases; for instance, the disease Marfan syndrome (MIM: 154700) is annotated with the HPO terms "arachnodactyly" (HP: 0001166), "ectopia lentis" (HP: 0001083), and 46 others. The patterns and specificity of the annotations allow the information content (IC) of each term to be calculated; the IC reflects the clinical specificity of the term and represents a key component of most of the aforementioned algorithms.[23] Additionally, computational logical definitions are provided for HPO terms. For instance, the HPO term "hypoglycemia" is defined on the basis of "decreased concentration" (PATO: 0001163) in "blood" (UBERON: 0000178) with respect to "glucose" (CHEBI: 17234); this definition uses terms from the ontologies PATO[24] for describing qualities, UBERON for describing anatomy,[25,26] and ChEBI for describing small biological molecules.[27] These definitions are useful for a number of applications, including cross-species phenotype comparisons[6,28,29] and computational quality control.[30]

[1]School of Information Technology and Electrical Engineering, University of Queensland, St. Lucia, QLD 4072, Australia; [2]Garvan Institute of Medical Research, Darlinghurst, Sydney, NSW 2010, Australia; [3]Institute for Medical and Human Genetics, Charité-Universitätsmedizin Berlin, Augustenburger Platz 1, 13353 Berlin, Germany; [4]University of Applied Sciences, Wiesenstrasse 14, 35390 Giessen, Germany; [5]Library, Oregon Health & Science University, Portland, OR 97239, USA; [6]School of Paediatrics and Child Health, University of Western Australia, Perth, WA 6840, Australia; [7]Institute for Immunology and Infectious Diseases, Murdoch University, Perth, WA 6150, Australia; [8]Office of Population Health Genomics, Public Health and Clinical Services Division, Department of Health, Perth, WA 6004, Australia; [9]Genetic Services of Western Australia, King Edward Memorial Hospital, Perth, WA 6008, Australia; [10]Telethon Kids Institute, Perth, WA 6008, Australia; [11]Institute of Bioorganic Chemistry, Polish Academy of Sciences, 61-704 Poznań, Poland; [12]Department of Epidemiology and Public Health, School of Medicine, University of Maryland, Baltimore, MD 21201, USA; [13]Institute for Genome Sciences, School of Medicine, University of Maryland, Baltimore, MD 21201, USA; [14]Center for Biomedical Informatics and Information Technology, National Cancer Institute, 9609 Medical Center Drive, Rockville, MD 20850, USA; [15]Department of Physiology, Development and Neuroscience, University of Cambridge, Downing Street, Cambridge CB2 3EG, UK; [16]The Jackson Laboratory, Bar Harbor, ME 04609, USA; [17]Department of Genetics, University of Leicester, Leicester LE1 7RH, UK; [18]European Bioinformatics Institute, European Molecular Biology Laboratory, Wellcome Trust Genome Campus, Hinxton, Cambridge CB10 1SD UK; [19]Academic Department of Medical Genetics, The Children's Hospital at Westmead, Sydney, NSW 2145, Australia; [20]Discipline of Genetic Medicine, Sydney Medical School, University of Sydney, Sydney, NSW 2145, Australia; [21]Genomics Division, Lawrence Berkeley National Laboratory, 1 Cyclotron Road, Berkeley, CA 94720, USA; [22]Max Planck Institute for Molecular Genetics, Ihnestrasse 63–73, 14195 Berlin, Germany; [23]Berlin Brandenburg Center for Regenerative Therapies, Charité-Universitätsmedizin Berlin, Augustenburger Platz 1, 13353 Berlin, Germany; [24]Institute of Bioinformatics, Department of Mathematics and Computer Science, Freie Universität Berlin, Takustrasse 9, 14195 Berlin, Germany
[25]These authors contributed equally to this work
*Correspondence: peter.robinson@charite.de
http://dx.doi.org/10.1016/j.ajhg.2015.05.020. ©2015 The Authors
This is an open access article under the CC BY license (http://creativecommons.org/licenses/by/4.0/).

The focus of the HPO has, to date, been on rare disease, and correspondingly, it has primarily been adopted by groups from various fields in human genetics, including the Sanger Institute's Deciphering Developmental Disorders database[22] and DECIPHER,[31] the European Cytogeneticists Association Register of Unbalanced Chromosome Aberrations,[32] the NIH Undiagnosed Diseases Program and Network, the rare-disease section of the UK's 100,000 Genomes Project, and Genome Canada's CARE for RARE program, but also by databases for genome-wide association studies (GWASs).[33–35] Along with rapid technological advances in the field of next-generation sequencing (NGS), personalized medicine is quickly becoming reality,[36] and initial attempts to use genome sequencing to predict phenotypic abnormalities in common, complex diseases are beginning to show promising results.[37] In this work, we have extended the range of the HPO from rare to common human disease in order to provide a computational foundation for phenotype-driven analysis of genomes and other translational research in the field of genetics of complex human disease. We have generated over 132,000 phenotypic annotations from the HPO for 3,145 common diseases by using a text-mining approach and have made them freely available to the community. Finally, we demonstrate the uses to which this resource can be put and set out a framework for the future development of the HPO as a community-driven resource for phenotypic analysis of rare and common disease.

Material and Methods

Extraction of HPO Terms By Automatic Concept Recognition

Concept recognition (CR) extracts ontology terms from text with the aim of leveraging structured knowledge from unstructured data. For example, CR might be able to identify the term "macrocephaly" (HP: 0000256) within an abstract that contains the phrase "large head" because the latter is listed as a synonym in the entry HP: 0000256. Published CR approaches rely on direct dictionary lookup combined with stemming and word-permutation algorithms[38] or use natural-language-processing pipelines with techniques such as sentence splitting, tokenization, and part-of-speech tagging.[39] In our experiments we used a CR tool specifically tailored to address the challenges of extracting phenotype concepts—the Bio-LarK Concept Recognizer.[40] Bio-LarK uses a two-step approach to index and retrieve ontology terms in combination with a series of language techniques to enable term normalization. In addition to providing standard CR, the system is able to decompose and align conjunctive terms (e.g., "short and broad fingers" aligns to "short finger" [HP: 0009381] and "broad finger" [HP: 0001500]), as well as recognize and process non-canonical phenotypes, such as "fingers are short and broad," which would be aligned to the same terms as in the previous example. Our current CR approach does not attempt to detect negation, which might represent a cause of false-positive results. However, because of the post-processing steps used to generate the final annotations on the basis of threshold values for annotation frequency and IC (see below), our procedure will not, in general, be sensitive to isolated negative assertions.

PubMed-MEDLINE 2014 Corpus

The CR process was performed on the 2014 release of the PubMed-MEDLINE corpus. The corpus contains 22,376,811 articles, of which 13,262,617 have a valid title and abstract (most of the missing entries represent articles in languages other than English and only their titles are listed). MEDLINE abstracts are associated with a series of medical subject headings (MeSHs); the main headings (descriptors) provide a schematic description of the topic of the article. The descriptors are divided into 16 categories, including category C, "diseases." Category C contains 4,620 unique entries, and we refer to it here as "MeSH diseases."

We note that although MeSH category C is described as comprising diseases, many of the terms in the complete tree C (4,620 entries) do not refer to specific diseases. For instance, many of the terms describe general categories, such as "brain diseases" (MeSH: D001927), veterinary diseases (e.g., "brucellosis, bovine" [MeSH: D002007]), and various other entities, such as "cadaver" (MeSH: D002102). Others represent phenotypic features of diseases rather than actual disease entities; one example is "Cheyne-Stokes respiration" (MeSH: D002639), which is an abnormal breathing pattern that can be observed in diseases such as central sleep apnea syndrome. We excluded such MeSH entries by careful manual curation, leaving a total of 3,145 MeSH category C descriptors that we judged to actually represent specific disease entries. Only these entries were used for the analysis described in this manuscript.

We filtered the 13,262,617 abstracts on the basis of the MeSH terms to retain only those abstracts that included at least one of the 3,145 disease entries from the MeSH disease list and then processed them with the Bio-LarK Concept Recognizer. In some cases, a single abstract was annotated with multiple MeSH disease terms, some of which were also featured as major topics for the article under scrutiny. For the purpose of this analysis, we included all abstracts independently of the number of associated MeSH terms or their major topic feature.

Filtering HPO Annotations

Many abstracts that describe a given disease also mention a certain HPO term. Consequently, that disease is more likely to be characterized by the corresponding phenotypic abnormality. For instance, the PubMed abstract with the PubMed identifier PMID: 23886833 is indexed with the MeSH term "encephalitis, herpes simplex," and parsing the record with Bio-LarK reveals a number of HPO terms, including "headache" (HP: 0002315). Therefore, one might be tempted to conclude that this type of encephalitis can be characterized by headaches, but from this single observation it cannot be guaranteed that the abstract is indeed making this assertion. The abstract could, for instance, be describing an adverse effect of a medication, a differential diagnosis, or one of a number of other things. We reasoned that if an HPO term were identified in multiple abstracts associated with a given disease from the MeSH disease list, then it would be more likely to represent a genuine phenotypic abnormality associated with the disease.

However, frequency alone is not a strong enough indicator of a correct association between a phenotype and a disease. Ideally, the phenotype should also be specific to (i.e., present only in a limited number of) certain diseases. Given this required balance, we developed a procedure that aims to distinguish the true annotations on the basis of three metrics: (1) the balance between frequency and specificity; (2) the IC of the term—i.e., the overall degree of

Input: S_{HPO} – Initial set of HPO terms associated with a disease (resulting from the concept recognition process)
Output: $FinalSet$ – ranked and clustered HPO terms
Parameters: n, m, e
1 Rank $t \in S_{HPO}$ using $TFIDF(t, D)$
2 $Seeds_{HPO} \leftarrow \{t \mid TFIDF(t, D) \geq \mathtt{Mean}_{TFIDF} + n \times \mathtt{SD}_{TFIDF}\}$
3 $Rest_{HPO} \leftarrow S_{HPO} - Seeds_{HPO}$
4 Group terms in according to their associated top-level HPO ancestor A – i.e., the most generic type of abnormality
5 **foreach** $A \in Toplevel_{HPO}$ **do**
6   find $O_A = \{t_1, t_2, \ldots, t_k\}, O_A \neq \varnothing, t \in Seeds_{HPO}, A \in Ancestor(t)$, such that O_A is the subset that minimizes $density(A)$
    // $density(A) = \mathtt{SD}\left[Path^{n}_{i=1,j=1,i\neq j}(t_i, t_j)\right]$
7   retain $\min(density(A))$
end
8 $FinalSet = \{O_A, O_A \neq \varnothing \mid A \in Toplevel_{HPO}\}$
9 Rank $t \in Rest_{HPO}$ using $TFIDF^{IC}(t, D)$
10 $Candidates_{HPO} \leftarrow \{t \mid TFIDF^{IC}(t, D) \geq \mathtt{Mean}_{TFIDF^{IC}} + m \times \mathtt{SD}_{TFIDF^{IC}}\}$
11 Group terms in $Candidates_{HPO}$ according to their associated top-level HPO ancestor A
12 **foreach** $A \in Toplevel_{HPO}$ **do**
13   **foreach** $t \in Candidates_{HPO}$ such that $A \in Ancestor(t)$ **do**
14         $O^t_A \leftarrow O_A + \{t\}$
15   **if** $\min(density(O^t_A)) \leq \min(density(O_A)) + e$ **then**
            $FinalSet \leftarrow FinalSet + \{t\}$
        end
    end
end
16 Remove duplicates from $FinalSet$
17 **Return:** $FinalSet$

Figure 1. Algorithm 1
Summary of the algorithm used to identify a set of HPO term annotated to diseases. See Material and Methods for explanations.

specificity of the term in our corpus of diseases; and (3) the disease-category-driven density of a subset of terms, based on the shortest path between them in the HPO. The balance between frequency and specificity is measured with a standard information-retrieval technique: term frequency, inverse document frequency (TFIDF). The TFIDF weighs HPO terms highly if they occur with high frequency among abstracts annotated to a disease but down-weighs terms that are common within the entire corpus (see the following section).

Figure 1 summarizes the algorithm we have developed. It takes as input the initial set of HPO terms and, using three tuning parameters, produces a final set of candidates. The three tuning parameters control term cutoffs at different stages: (1) *n*, which defines the initial TFIDF threshold used for creating the clustering seeds; (2) *m*, which defines a second specificity threshold (over TFIDF^{IC}; see following section) used for pruning terms left over from the first threshold; and (3) *e*, which defines the density margin that dictates the inclusion or exclusion of a term in a cluster.

The algorithm consists of three steps. First, the initial set of terms is filtered with TFIDF for the creation of clustering seeds (lines 1–3). Second, these clustering seeds are grouped according to their common top-level HPO ancestor —i.e., the top-level HPO abnormality (e.g., blood or skeletal system; line 4). The intuition here is that most diseases affect, in principle, a very limited number of major organs, and hence, most true positives will be grouped according to these major organs (corresponding to the top-level HPO phenotypic abnormality terms). Once the clustering seeds are grouped, we look for the group-based subset of terms that form the single shortest ontological path among them (i.e., the sub-group with the minimum density; lines 5–7). This can be seen as an inverse analogy to the traveling salesman problem, where the shortest path between two terms (i.e., the number of edges required to connect them in the HPO) denotes the cost, and the goal is to minimize the SD of the array of shortest paths. We adapted the Hungarian algorithm to solve this problem. The resulting subset is added to the final list of candidates (line 8).

Finally, the list of terms initially filtered out with TFIDF is pruned with TFIDF^{IC} (lines 9 and 10), and the terms are grouped according to the top-level HPO abnormalities in the same manner as the clustering seeds (line 11). Incrementally, using the group-based density and set of seeds computed in the previous step, we append each leftover term to the seed subset and compute an aggregated density. If the new density is within the limits established by the density margin error parameter (*e*) with respect to the seed density, then the term is added to the final candidates (lines 12–15).

Given a gold-standard corpus, one of the main advantages of this algorithm is the opportunity for learning diverse values for the three parameters, subject to a particular goal. For example, the above-mentioned assumption (i.e., diseases affect a very limited set of major organs) can be transformed into a learning task based on disease categories. We experimented with the 41 manually curated diseases, split into 13 categories dictated by the top-level terms (e.g., cardiovascular diseases, integumentary system diseases, etc.) in the Disease Ontology (DO), and aimed to maximize the category-based true-positive rate. This can be realized by learning sets of parameters corresponding to each disease category. The experimental results showed an overall resulting precision of 66.8%, including highlights such as over 70% precision for diseases by infectious agents (73.0%), diseases of the nervous system (77.8%), or immune system diseases (82.8%). Similarly, we experimented with targeting a maximized overall F-score (i.e., the harmonic mean of precision and recall—a balance between coverage and true-positive rate) and achieved a value of 45.1%. This value is equivalent to an average precision of around 60% associated with a recall of around 40%.

Information Theoretic Measures for HPO Annotations

The algorithm in Figure 1 uses several information theoretic measures, discussed below.

TFIDF is a standard information-retrieval metric for ranking terms on the basis of their co-occurrence and specificity in the context of a given set of documents. In our case, the goal is to rank HPO terms according to their frequency and specificity in the context of a particular disorder. TFIDF is adapted below (to take into account the disorder-specific context), where t denotes an HPO term, D denotes the disease under scrutiny, and T_D represents the total number of disorders (i.e., 3,145).

$$\text{TFIDF}(t, D) = \text{TF}(t, D) \times \text{IDF}(t, D)$$

$\text{TF}(t, D)$, the term frequency of HPO term t for disease D, is defined as the number of D-associated abstracts in which a term t appears at least once (regardless of the number of mentions in a particular abstract), and the inverse document frequency,

IDF(t, D), is defined as the logarithm of the quotient of the total number of diseases (T_D) divided by the number of diseases for which the HPO term in question is mentioned in at least one abstract.

$$\mathrm{IDF}(t,D) = \log\frac{T_D}{|\{d\in D\ :\ t\in d\}|}$$

The IC of an individual HPO term within the MEDLINE corpus can be estimated with its frequency among annotations of the entire corpus. Intuitively, the IC of a term such as "fever" (HP: 0001945) is less than that of a term such as "aortic arch calcification" (HP: 0005303) because fewer diseases are characterized by the latter abnormality, and so knowing that an individual has aortic arch calcification narrows down the differential diagnosis much more than knowing that an individual has fever. For each term t of the HPO, the IC is quantified as the negative logarithm of its frequency: $\mathrm{IC}(t) = -\log p(t)$. If a disease is annotated with any term t in the HPO, it must also be annotated with all the ancestors of t. Therefore, the IC of terms is calculated on the basis of annotations with the term or any of its descendants in the HPO.[41] For instance, if seven of 1,000 abstracts are annotated with a certain HPO term t', and three more abstracts are annotated with descendants of t', then the frequency of the term would be calculated as $p(t') = 10\ /\ 1{,}000$, and the IC of the term would be calculated as $\mathrm{IC}(t)^{'} = -\log p(0.01)$. The higher (i.e., closer to the root) in the ontology a term is located, the lower its IC. We use this as an additional term to define TFIDF^{IC} for HPO term t and disease D as

$$\mathrm{TFIDF}^{\mathrm{IC}}(t,D) = \mathrm{TFIDF}(t,\ D) \times \mathrm{IC}(t).$$

Calculation of Phenotypic Overlap with an Extended Jaccard Index

The Jaccard index is a standard measure of similarity between two sample sets, A and B, and is defined as the size of the intersection divided by the size of the union of the sample sets:

$$J(A,B) = \frac{|A\cap B|}{|A\cup B|}.$$

The value of the Jaccard index ranges from 0 for complete dissimilarity to 1 for identity. In a typical set-based context, the Jaccard index is computed on the strict intersection and union of the elements. However, in our context these elements represent ontology terms, structured in a logical hierarchy. And, as such, we can rely on the subsumption relation between terms when computing intersection and union. We exploited this aspect in the computation of the Jaccard index. A match between two terms was recorded not only when the two terms matched exactly (i.e., "cranial hyperostosis" is the same as "cranial hyperostosis") but also when the subsumption relation was present (i.e., "cranial hyperostosis" is a parent of "calvarial hyperostosis" and an ancestor of "mandibular hyperostosis"; Figure S1).

Validation of HPO Annotations for Common Disorders

We chose three to five common diseases from each of the 13 DO upper-level categories used in our common-disease network (CDN; see below) for a total of 41 diseases. We used a Perl script to choose diseases at random from among all diseases in the categories. We examined the diseases manually by assessing each HPO term mentioned at least once in any abstract describing the disease in question (thus, we evaluated substantially more HPO terms than merely the set of terms chosen by our annotation pipeline on the basis of frequency and specificity of the term). Biocuration was performed by N.V., G.B., D.V., A.Z., M.H., and P.N.R., and all annotations were validated by P.N.R., who is both a computer scientist and a medical doctor. This allowed us to assess the true-positive, false-positive, and false-negative rates as shown in Tables S1–S41.

CDN

In order to validate and visualize the phenotype annotations obtained for common disease, we constructed a CDN by computing the pairwise similarity of a total of 1,678 diseases (i.e., annotated MeSH entries) belonging to 13 DO categories such as "nervous system disease" (DOID: 863) or "respiratory system disease" (DOID: 1579) (Figure S2). Note that some diseases belong to multiple DO classes (Figure S3).

For each disease, we obtained all the HPO annotations that our CR algorithm had associated with the disease. The annotation frequency of a term was defined as the proportion of diseases that were annotated by the term or any of its descendent terms. In order to calculate similarity between two terms (t_1, t_2), we used the IC of their most informative common ancestor (MICA),[3] denoted as $\mathrm{MICA}(t_1, t_2)$.

We used the above-mentioned term-similarity measures to calculate a semantic-similarity score for two diseases (D_1, D_2). In our case, for each of the terms of D_1, the "best match" among the terms annotated D_2 was found, and the average overall query terms was calculated. This was defined as the similarity:

$$\mathrm{sim}(D_1 \rightarrow D_2) = \mathrm{avg}\Big[\sum_{s\in D_1} \max_{t\in D_2}\ \mathrm{IC}(\mathrm{MICA}(s,t))\Big],$$

where the average was taken over all terms s to which disease D_1 is annotated. Note that this score is asymmetric, i.e., it is not necessarily the case that $\mathrm{sim}(D_1 \rightarrow D_2) = \mathrm{sim}(D_2 \rightarrow D_1)$. Therefore, for the analysis described here, we used a symmetric similarity score:

$$\mathrm{sim}(D_1, D_2) = \frac{1}{2}\mathrm{sim}(D_1 \rightarrow D_2) + \frac{1}{2}\mathrm{sim}(D_2 \rightarrow D_1).$$

The CDN consists of nodes that represent common diseases and edges that indicate that two diseases are phenotypically similar. In order to create the CDN, we calculated the symmetric similarity score for all pairs of diseases. The network was visualized with the force-directed layout algorithm of Cytoscape,[42] whereby an edge between nodes was drawn if the similarity between two corresponding diseases exceeded 2.0 (simulation cutoff [simcut]). The final CDN (CDN-o) consisted of 1,148 diseases and 4,059 edges.

Statistical Significance of the CDN

In order to test the statistical significance of the distribution of phenotypic similarity among diseases within the same disease category or between different categories, we introduced the concept of the gray-edge fraction (GEF). That is, we visualized edges between nodes (diseases) that do not belong to one of the same 13 general disease categories as gray edges. The GEF was defined as the proportion of gray edges among all edges in the CDN. The lower the GEF, the better the phenotypic clustering of diseases agrees with the classification of the diseases into the 13 categories. The original CDN (CDN-o) comprised 3,547 edges, 998 of which were gray edges, corresponding to a GEF of 0.246 (red arrow in Figure S4A). We tested two randomization procedures, edge randomization (er) and annotation randomization (ar).

The edge-permutation procedure retains the number of edges and the degree distribution of the network.[43] Two edges, A-B and X-Y, are chosen at random and reshuffled to create the edges A-Y and X-B. Reshuffling is skipped if the edges A-Y and X-B already exist. Reshuffling is performed 10,000 times, resulting is an edge-randomized version of CDN-o, which we call CDN-er and for which we can again compute the GEF. We constructed 1,000 versions of CDN-er and plotted the distribution of the resulting GEF values in Figure S4A. As one can see, the p value of the CDN is less than 0.001 because none of the edge-randomized CDNs achieved the same or a smaller GEF than the original CDN.

We additionally performed a test in which we randomized the HPO terms associated with each disease (ar). For this, we randomly selected 50% of the terms associated with each disease and replaced them with randomly selected HPO terms. We computed the randomized CDN (called CDN-ar) by using the above procedures used to construct the CDN-o. We repeated this procedure 100 times and computed the GEF for each CDN-ar. Note that each CDN-ar might not have the same amount of nodes and edges as the CDN-o. When using the same simcut (2.0) used for constructing the CDN-o, we obtained much smaller networks (fewer than 100 nodes). The distribution of GEF values of CDN-ar with simcut 2.0 is shown in Figure S4B. No CDN-ar achieved a GEF less than or equal to the CDN-o GEF, which corresponds to a p value of less than 0.01. We modified the simcut to 1.4 because it leads to CDN-ar versions with approximately the same amount of nodes as CDN-o. The distribution of the resulting GEF values is shown in Figure S4C. Again, not a single CDN-ar constructed with a simcut of 1.4 achieved a GEF less than or equal to the CDN-o GEF, which corresponds to a p value of less than 0.01.

GWAS Data

GWAS Central provides a comprehensive collection of summary-level genetic-association data and advanced visualization tools to allow comparison and discovery of datasets from the perspective of genes, genome regions, phenotypes, or traits.[33] The project collates association data and study metadata from many disparate sources, including the National Human Genome Research Institute GWAS Catalog,[35] and receives frequent data submissions from researchers who wish to make their research findings publicly available. All gathered and submitted data are extensively curated by a team of post-doctoral genetics researchers who manually evaluate each study for its range of phenotype content and apply appropriately chosen MeSH terms. As of December 2014, the resource contained 69 million p values for over 1,800 studies.

Data and metadata for up to 1,000 associations can be freely downloaded from the BioMart-based system (GWAS Mart), and larger custom data dumps (up to and including the complete database) are available via contacting the GWAS Central development team and agreeing with a data-sharing statement. Thus, to provide data for the present study, we generated a tab-separated file representing 1,574 studies and 34,252 unique SNPs (annotated to 675 unique MeSH terms) and containing the GWAS Central study identifier, PubMed identifier, dbSNP "rs" identifier, p value, and MeSH identifier for all associations with $p < 1 \times 10^{-5}$. We compiled the list of genes considered for our experiments by retrieving the "mapped genes" column from the database SCAN and identifying those genes corresponding to the GWAS Central SNPs. Where no mapped genes were reported, we used the upstream, as well as downstream, genes listed by SCAN.[44]

Results

Generation of Phenotype Annotations for Common Disease by CR

We applied a phenotype-aware CR system (the Bio-LarK Concept Recognizer[40]) to all available abstracts in PubMed in order to extract phenotypic annotations for common diseases. We first retrieved the MeSH terms associated with PubMed abstracts and used them to retain only those abstracts focused on diseases. 5,136,645 of 22,376,811 articles listed in PubMed had an abstract and could be assigned to such a MeSH disease term (see Material and Methods for a description of our inclusion criteria for MeSH disease entries; a total of 3,145 diseases were included). Second, we applied CR on the resulting set, after which a total of 930,805 HPO annotations were assigned to 3,145 common diseases. Finally, we filtered this initial set of HPO terms, by using a ranking-and-clustering method with the aim of maximizing the F-score computed on a manually curated gold-standard set of 41 common diseases (see Material and Methods). This approach aims to maximize the text-mining accuracy, defined as the harmonic mean of the precision and recall of the derived annotations. This final set comprised 132,006 HPO annotations covering 4,459 unique HPO terms. The mean number of annotations per disease was 41.97 (range, 1–271; median, 32) and consisted of terms belonging to all of the top-level HPO categories (Figure S5). Figure 2 provides an overview of the analysis procedures used to generate and validate the common-disease annotations.

As an example, Table S1 lists the annotations produced for "giant cell arteritis" (MeSH: D013700), which includes terms such as "vasculitis" (HP: 0002633), "granulomatosis" (HP: 0002955), and "amaurosis fugax" (HP: 0100576). The annotations are highly accurate, although some nuances are not detected by the CR process. For instance, "facial palsy" (HP: 0010628) and "renal amyloidosis" (HP: 0001917) are classic manifestations of giant cell arteritis. The list of phenotypic manifestations is by no means complete, given that it failed to identify manifestations such as "dysphagia" (HP: 0002015), "trismus" (HP: 0000211), and "encephalopathy" (HP: 0001298). Nonetheless, the CR process was able to capture a largely accurate subset of phenotypic abnormalities for giant cell arteritis, such that 64% of the annotations were true positives.

We estimated the overall quality of the HPO annotations by inspecting the automatically extracted annotations for a set of 41 common diseases randomly chosen from 13 upper-level DO[45] categories that had a MeSH disease identifier and thus could be analyzed analogously to the common MeSH diseases. The process involved manually validating of all HPO annotations extracted by the CR process and comparing them to the results of detailed manual curation

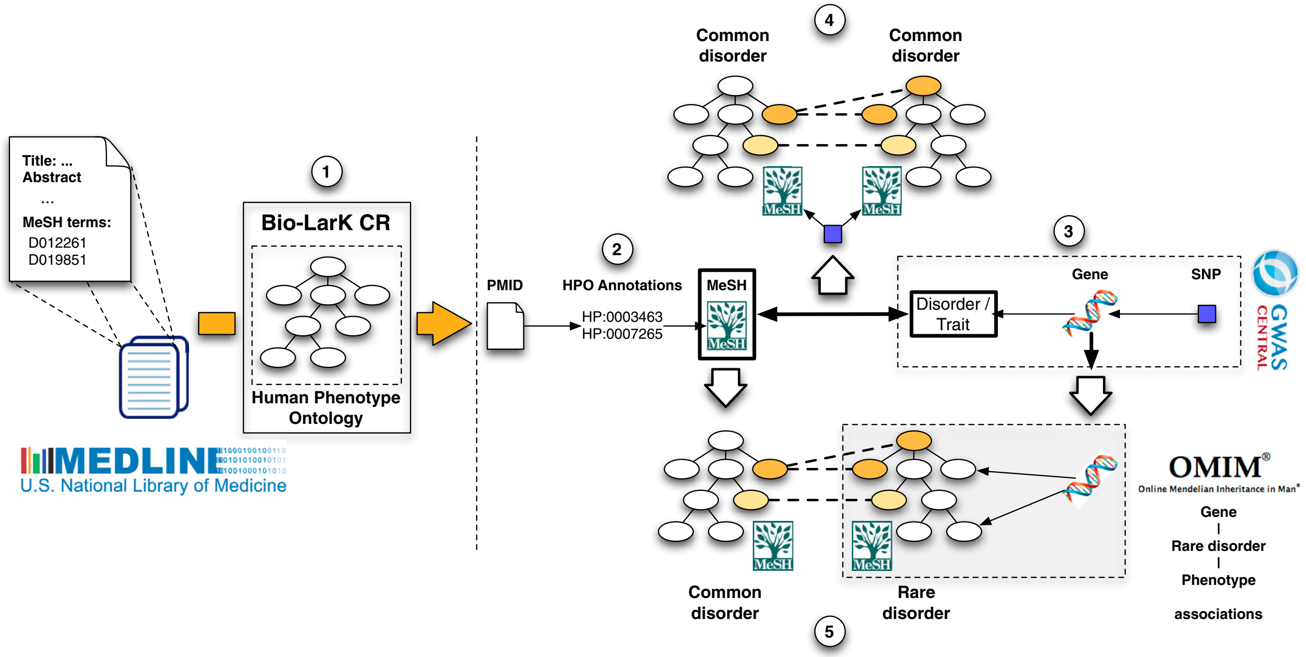

Figure 2. Overview of CR and Bioinformatic Analysis

The analysis was performed in several major steps. (1) Bio-LarK was used to analyze the PubMed-MEDLINE 2014 corpus, which resulted in a total of 5,136,645 abstracts annotated with MeSH terms and phenotypic features. (2) For each of 3,145 resulting diseases, the frequency and specificity of HPO terms found in the abstract were used for inferring phenotypic annotations. (3) These annotations were used for producing disease models for each of the diseases. (4) Medical validation of the annotations was performed on the basis of disease, phenotype, and SNP annotations in GWAS Central for phenotype sharing in common disease. (5) Validation with OMIM, Orphanet, and DO was used for assessing phenotype sharing between rare and common diseases linked to the same locus.

for the estimation of the true- and false-positive and the false-negative rates. We note that it is not informative to calculate a true-negative rate across the entire HPO because even if the CR process flags several hundred terms, the great majority of the over 10,000 HPO terms will be true negatives. We found that maximizing the overall F-score (i.e., the harmonic mean of precision and recall) led to a mean F-score of 45.1% (i.e., a mean precision of around 60% accompanied by a mean recall of around 40%). In separate experiments, we found that a CR run with parameters designed to maximize the precision in each of the 13 categories achieved a mean precision of 66.8% (data not shown). However, we chose to use the annotations derived from the F-score procedure for the remainder of the analysis. The complete set of annotations associated with the 41 common diseases, including flags for true positives, false positives, and false negatives, can be found in Tables S1–S41.

A Common-Disease Phenotypic Network

As a first test of the medical validity of the HPO annotations for common-disease phenotypes, we visualized the network of phenotypic similarity of a subset of 1,678 diseases, such as “nervous system disease” (DOID: 863) or “respiratory system disease” (DOID: 1579), belonging to 13 DO categories. 1,148 of the 1,678 diseases showed at least one connection to another disease (phenotypic similarity score above a threshold of 2.0), and thus the final CDN comprised 1,148 diseases. Phenotypic relationships between these diseases are shown by the linking of all pairs of diseases exceeding the threshold similarity score (Figure 3). Although generated independently of the disorder classes, the resulting phenotypic network clearly displays clusters corresponding to the disease categories.

We then constructed randomized phenotypic networks as described in the Material and Methods and calculated the number of links between diseases from the same disease category. We found that the observed correlation between network connections and disease class is highly significant (Figure S4). Thus, the phenotypic network of common diseases, as defined by the HPO, is made up of dense clusters of shared phenotypic features that show characteristic patterns of interconnections between selected areas of the phenotypic continuum, just as we had previously observed for Mendelian diseases.[2] The high correlation between the computationally created network clusters and the manually curated disease classifications provides further evidence that the automatically created annotations are clinically meaningful and provide a largely correct description of the disease in question.

Phenotypic and Genetic Overlap across Complex Diseases

GWASs have been performed for a wide range of common diseases and traits, and over 6,000 strong SNP associations ($p < 10^{-8}$) have been identified.[35] Variation at multiple genetic loci collectively influences the likelihood of

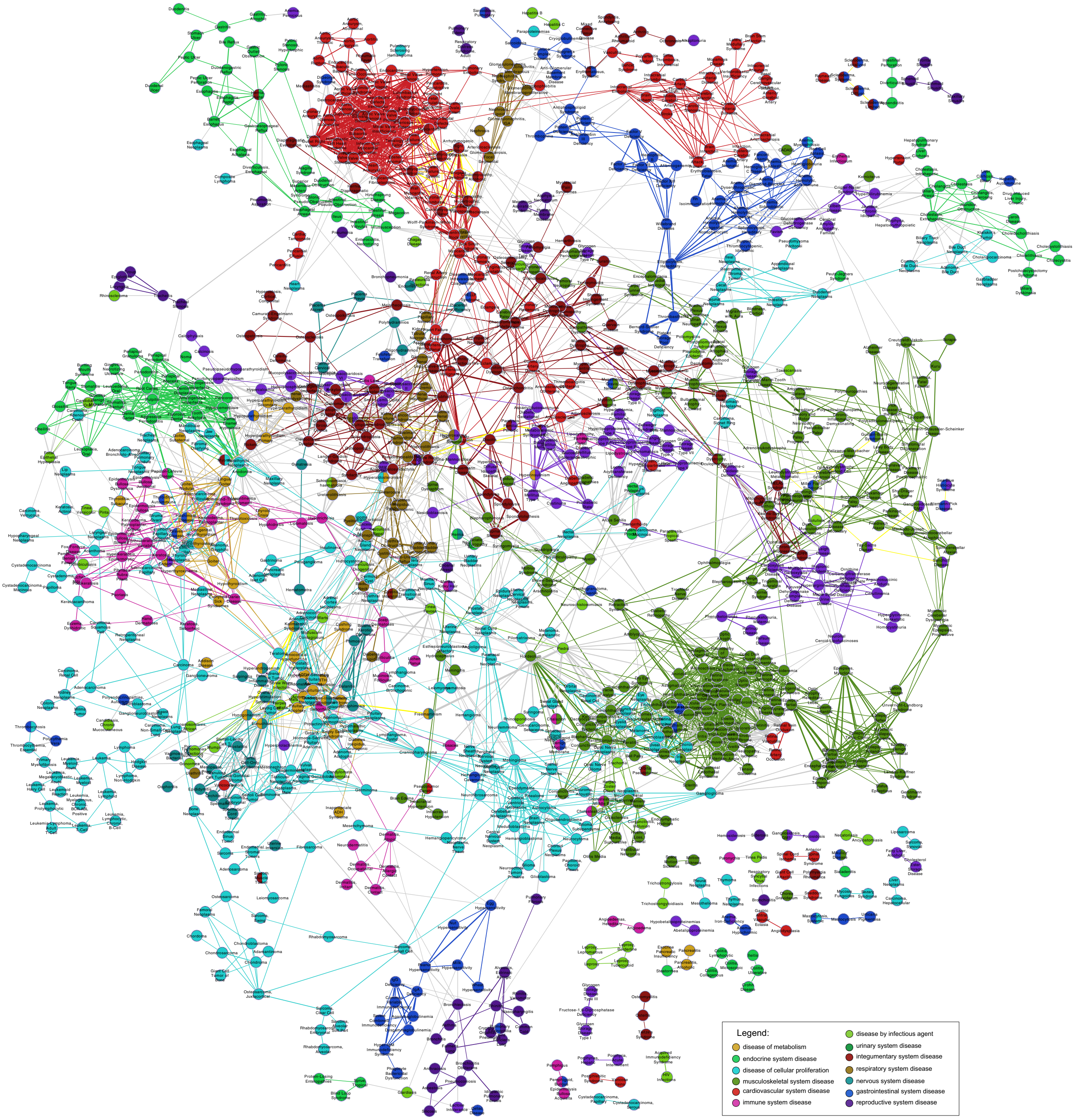

Figure 3. Phenotypic Network of Common Disease
A total of 1,678 common diseases could be mapped to at least one of 13 top-level DO categories (Figures S5 and S6). 1,148 of these diseases displayed a connection to another disease with a phenotypic similarity score of at least 2.0. They are shown as a node in the graph and are colored according to membership in the upper-level disease categories. The thickness of the connections between the nodes reflects the degree of phenotypic similarity

developing many common and complex diseases; for instance, it is estimated that that about 8,300 independent and predominantly common SNPs contribute to risk for schizophrenia[46] (MIM: 181500). Although the genetic architecture is likely to differ for different diseases, often the trait architecture consists of a few loci of relatively large effect and many additional loci that have a very small effect on phenotype.[47] To understand the genetics of complex disease, it is important to consider the phenotypic and genetic overlap among diseases. For instance, susceptibility loci that are common to both multiple ulcerative colitis and Crohn disease have been identified by GWASs, and some of these loci are even shared with several other autoimmune disorders.[48] Similarly, several psychiatric disorders share risk loci.[49] The study of the distribution of overlapping loci within a group of diseases might suggest shared pathways

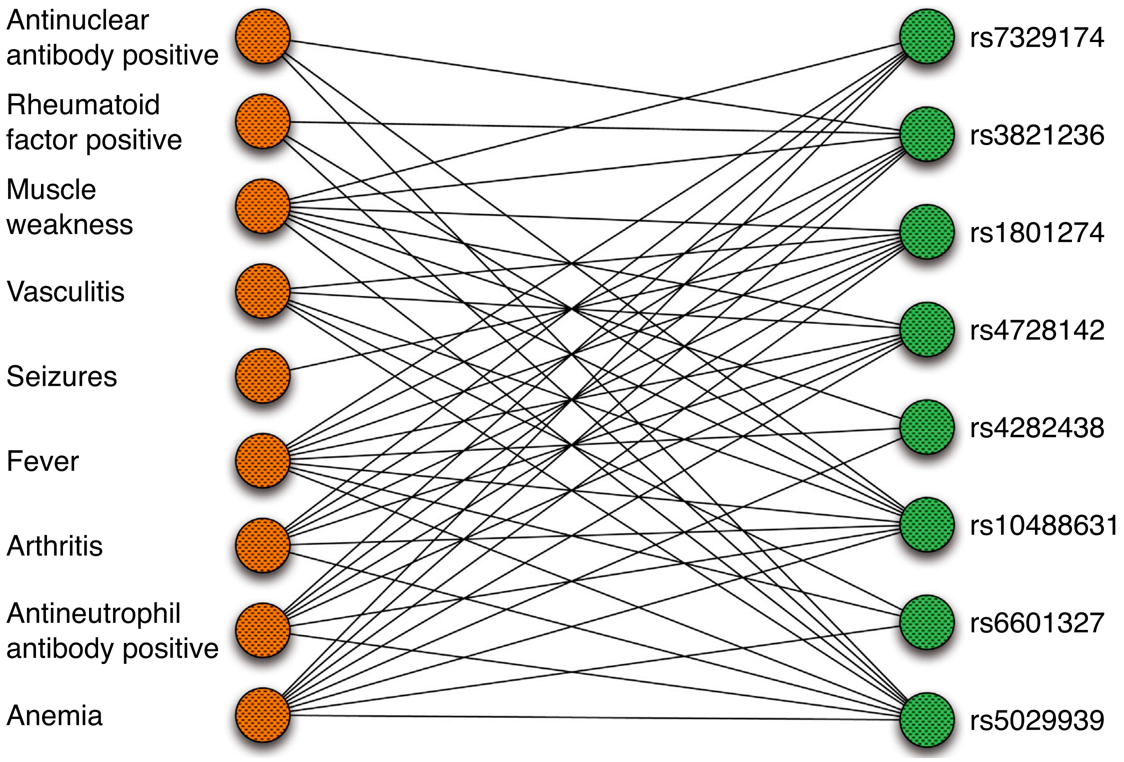

Figure 4. Phenotype-SNP Network
For constructing this network, individual HPO terms were connected to SNPs if the SNP was significantly associated with a disease characterized by the HPO term in question. For instance, the SNP rs5029939 is significantly associated with both Sjögren syndrome[51] and systemic lupus erythematosus.[52] The diseases also share a number of phenotypic features, including "antinuclear antibody positivity" (HP: 0003493) and "xerostomia" (HP: 0000217). A small and particularly dense subset of the network was manually chosen. The network is centered on ten HPO terms representing clinical features that are common in autoimmune diseases.

and common pathogenetic features.[23] On the other hand, the lack of overlap of other loci could help to identify pathogenic mechanisms that are unique to specific diseases and could help to explain phenotypic diversity across the spectrum of diseases in fields such as autoimmunity or psychiatry.[50] The computational resources presented here offer a tool for comprehensively measuring the phenotypic overlap of a wide range of common diseases that share risk loci.

From the total of 16,152 unique SNPs, 863 were associated with more than one disorder, and the total number of unique disorders was 300. 673 SNPs were associated with two disorders, 130 were associated with three, and 60 were associated with more than four (Figure S6). 577 of these SNPs were associated with a total of 79 unique diseases in our corpus and were used for the following analysis.

The mean Jaccard index for the pairwise comparison on the 577 SNPs was 0.251 ± 0.132. That is, for each pair of SNPs, the phenotypic annotations of the corresponding diseases were compared to each other with the extended Jaccard index (Figure S1). Randomly chosen disease comparisons from the existing pool of MeSH diseases displayed a significantly lower overlap of 0.130 ± 0.094 ($p = 2.29 \times 10^{-57}$, paired t test). Our results show a pervasive phenotypic sharing among complex diseases that are also associated with the same SNP. As an example, we show an excerpt of the phenotype-SNP network centered on autoimmune phenotypes. Ten phenotypic abnormalities observed in persons with these diseases are shown together with SNPs associated with one or more diseases displaying these features, such as Sjögren syndrome (MIM: 270150) and systemic lupus erythematosus (MIM: 152700). It can be seen that there is a dense interconnected network of phenotypes and SNPs (Figure 4). These results extend recent findings concerning a human disease-symptom network based on 322 individual symptoms extracted from MeSH.[53] We provide a CDN browser that allows users to navigate through the network of common diseases that are interconnected by phenotypic similarity (Figure S7).

Phenotypic and Genetic Overlap across Complex and Mendelian Diseases

Numerous, highly penetrant mutations in individual genes have been identified in thousands of Mendelian diseases. Common variants associated with complex diseases are enriched in genes mutated in Mendelian diseases.[54] For instance, certain mutations in presenilin 1 (*PSEN1*) cosegregate with early-onset familial Alzheimer disease[55] (MIM: 607822), whereas variants in the *PSEN1* promoter are associated with increased risk for complex (non-Mendelian) Alzheimer disease.[56] Similarly, common polymorphisms associated with blood lipoprotein concentrations are often located in the genomic vicinity of genes

Table 1. Phenotypic Overlap between Rare and Complex Disorders

Gene: Associated Rare Disease	Reference SNP: Complex Disease	Common HPO Terms
CD247: immunodeficiency due to defect in CD3-ζ (MIM: 610163)	rs840016: rheumatoid arthritis[59]	edema (HP: 0000969), arthralgia (HP: 0002829), arthritis (HP: 0001369), autoimmunity (HP: 0002960)
FSHR: ovarian hyperstimulation syndrome (MIM: 608115) and ovarian dysgenesis 1 (MIM: 233300)	rs2268361: polycystic ovary syndrome[60]	abnormality of the ovary (HP: 0000137), decreased fertility (HP: 0000144), primary amenorrhea (HP: 0000786)
PPARG: lipodystrophy, familial partial, type 3 (MIM: 604367)	rs13081389: type 2 diabetes mellitus[61]	hyperglycemia (HP: 0003074), hyperinsulinemia (HP: 0000842), hypertension (HP: 0000822)
LPL: type I hyperlipoproteinemia (MIM: 238600)	rs295: metabolic syndrome X[62]	hypercholesterolemia (HP: 0003124), hyperlipoproteinemia (HP: 0010980), coronary artery disease (HP: 0001677), pancreatitis (HP: 0001733)
LRRK2: Parkinson disease 8 (MIM: 607060)	rs34778348: Parkinson disease[63]	rigidity (HP: 0002063), bradykinesia (HP: 0002067), dementia (HP: 0000726), resting tremor (HP: 0002322)
HCN4: sick sinus syndrome 2 (MIM: 163800)	rs7164883: atrial fibrillation	arrhythmia (HP: 0011675), tachycardia (HP: 0001649), sinus brachycardia (HP: 0001688)
HYDIN: ciliary dyskinesia, primary, 5 (MIM: 608647)	rs12149070: COPD[64]	respiratory tract infection (HP: 0011947), respiratory insufficiency (HP: 0002093), bronchiectasis (HP: 0011947)

GWAS hits localized in the vicinity of Mendelian-disease-associated genes could be associated with common diseases that have phenotypic overlaps with the corresponding Mendelian diseases. Seven examples in which common and rare diseases linked to neighboring loci and showed substantial phenotypic overlap were manually chosen. The protein-coding gene associated with the rare disease, as well as the accession number of the polymorphism located in non-coding sequence near the gene, is shown. The following abbreviation is used: COPD, chronic obstructive pulmonary disease.

associated with Mendelian disorders of lipoprotein metabolism, such as *ABCG8*, *LCAT*, *APOB*, *LDLR*, *PCSK9*, *CETP*, *LPL*, *LIPC*, and *ABCA1*.[57,58] We therefore reasoned that the phenotypic-genetic overlap might be a general tendency for rare and common diseases located at the same genetic locus. As per the method described above, we examined 485 genes shared between the complex- (GWAS) and rare-disease datasets. GWAS SNPs were previously mapped to genes with SCAN.[44] In a manner similar to that used in the common-disease-phenotype experiment, we then measured the phenotypic overlap between the complex diseases from GWAS Central[33] and rare, Mendelian diseases associated with the genes in question. The overlap measure used in the experiments was the Jaccard index and was computed in the same manner as in the case of the complex-disease overlap. This resulted in a mean value of 0.027 ± 0.032, which was higher than the corresponding value for randomized pairs of common and rare disease (same procedure as above), 0.021 ± 0.023 ($p = 1.6 \times 10^{-7}$, paired t test). Table 1 shows some examples of GWAS hits that are linked to genes in which mutations cause Mendelian diseases with phenotypic overlap.

Discussion

Translational research in Mendelian diseases has benefited enormously from databases of the phenotypic features associated with individual diseases, such as OMIM,[65] Orphanet,[66] and more recently the HPO.[1,2] Analysis of such data has led to the idea that diseases that display similar phenotypic features are caused by mutations in functionally related genes. For instance, genetically heterogeneous diseases such as Fanconi anemia, Bardet-Biedl syndrome, or Usher syndrome are related to mutations in genes of a single biological module. Such modules can be a multiprotein complex, a pathway, or a single cellular or subcellular organelle.[67–70] To date, however, it has been difficult to perform analogous research on complex-disease phenotypes because resources to carry out comparable analyses have been lacking.

GWASs emerged in the first decade of the new millennium as a powerful tool for elucidating the genetic architecture of common disease.[33,35] The advent of clinical whole-genome sequencing[71] (WGS) is promising to lead to personalized genomic medicine. It is becoming apparent that precise phenotype analysis can substantially improve the ability to interpret the results of NGS. In rare diseases, for instance, diagnostic NGS yields plausible candidate variants in several genes, and making diagnoses will require that the consequences of these variants be analyzed and integrated with clinical findings.[72] In fact, using the HPO to analyze phenotypic data has been shown by multiple groups to improve the ability of NGS-based methods to identify candidate disease-associated genes and make clinical diagnoses.[5–11,21] These methods have been tested on

exomes and large NGS gene panels. In contrast, WGS provides a nearly comprehensive view on non-coding variations, a class of variation that makes up the majority of known risk factors for common disease.[35] WGS currently cannot be used reliably for the prediction of common disease in a clinical diagnostic setting.[73] However, this is increasingly becoming a topic of bioinformatics research[37,74,75] and is likely to increase in importance as large-scale efforts such as the UK's 100,000 Genomes Project begin to produce and interpret data. We speculate that phenotype analysis will be just as beneficial to WGS-based diagnostics of common disease as it has been shown to be for rare disease.[5–11,76,77] One area of particular interest stems from the observation that genes harboring common variants associated with a common disease might also carry large-effect mutations in a subset of individuals at the extremes of the trait. For instance, the polymorphism rs6817105, which is located about 167,000 nt upstream of *PITX2*, was found to be associated with atrial fibrillation.[78] More recently, a de novo nucleotide substitution in the promoter region of *PITX2* (319 nucleotides upstream of the transcription start site) was identified in an individual with severe atrial fibrillation.[79] Observations such as this and those summarized in Table 1 suggest that rare-disease phenotypes will be extremely useful in evaluating the findings of WGS performed on individuals with common, complex diseases and underline the utility of annotating rare and common diseases with a common phenotype ontology.

To generate the resource, we developed a statistical framework to evaluate the pattern of co-occurrences of HPO terms (phenotypic features) and diseases in PubMed abstracts. Previous efforts in the field of clinical text mining have shown the enormous promise of data extraction from articles or electronic health records (EHRs) for translational research; one of the keys to tapping this resource lies in the ability to reliably extract clinical information from the EHRs by text mining and other methods.[80] For instance, phenome-wide association scans (PheWASs) search EHRs for disease-gene associations by using the International Classification of Disease (ICD9) billing codes, which are available in most EHR systems, and have been shown to be able to replicate findings of traditional GWASs and identify novel associations.[81,82] Other groups have used EHR data to detect adverse medication interactions.[83] The project presented here had different goals, in that we developed a statistical model to infer the spectrum of phenotypic abnormalities that characterize diseases rather than to classify individuals' records according to whether a certain disease was present or not (as has been the case for the majority of the PheWASs and similar studies published to date; we note that many of these studies utilized the word "phenotype" to refer to a disease entity, whereas our study has examined the individual phenotypic features of diseases).

The algorithms we developed to derive disease models from the annotation patterns of PubMed abstracts combined a number of components, including (1) semantic CR (Bio-LarK[40]); (2) an adaptation of the TFIDF method, whereby diseases take the place of documents, and the "document frequency" of individual HPO terms is calculated from the number of abstracts containing the term; (3) an evaluation of the IC of individual HPO terms for calculating the semantic similarity[84,85] between terms; and (4) a heuristic graph clustering method that attempts to extend seed terms with particularly high TFIDF values to create a dense phenotypic network. This allowed us to develop annotations for over 3,000 common, complex diseases, and we demonstrated the potential utility of the resource by an analysis of phenotypic overlap between common and rare disease, as well as between complex diseases that share one or more genetic associations. The platform we have made available, together with the data, is in itself a valuable resource for the community. In addition to providing a way to download the data in a tab-separated form, or to access it programmatically via application programming interfaces, the website also enables a phenotype- and disorder-centric browsing of MEDLINE abstracts and browsing within the CDN (Figure S7). This resource could be useful for physicians who are caring for persons with a given disease and who present with a particular manifestation or complication of that disease (denoted by an HPO term). The browser will present all PubMed abstracts that were identified in our study and that describe both the disease and the phenotypic manifestation, which might provide information that could be helpful in clinical management.

There are several limitations of the common-disease annotations that we have presented here. First and foremost, the annotations were derived by a computational CR (text-mining) process and contain both false-positive and false-negative annotations. The HPO project, which is being developed as a part of the Monarch Initiative, will be actively revising and expanding the annotations and developing new areas of the ontology itself as needed for the analysis of common disease, much as it has been doing in the field of rare diseases since 2007.[1,2] Several characteristics of particular importance to common diseases, such as the past medical history and the time course of disease, are not currently well captured by the computational data structures and algorithms that have been developed for rare disease and will need to be established in future work. The results of the analysis of phenotypic overlaps are highly statistically significant but do not provide proof of a common pathophysiological basis of the diseases involved. However, we contend that the results we have presented in this manuscript demonstrate that the common-disease HPO annotations can be used for the computational analysis of phenotypic abnormalities across a previously unheard-of range of rare and common diseases, including over 7,000 rare diseases and 3,145 common diseases. To the best of our knowledge, there is no comparable computational resource that provides both an extensive phenotype ontology and annotations to over 10,000

diseases, as well as an algorithmic basis for calculating the similarity between arbitrary sets of phenotypic abnormalities and specific diseases[3] and a foundation for translational research on topics such as cross-species phenotype mapping.[6,23]

The HPO project has been under development since 2007 and has mainly focused on rare and primarily Mendelian diseases.[1,2] The work presented here provides users with over 132,000 phenotypic annotations for 3,145 common diseases derived via text mining. It is hoped that these annotations, as well as the underlying HPO terms, will be useful for both clinicians and researchers. Future work will include biocuration efforts to validate and extend the current set of annotations, to add metadata such as the age of onset, severity, clinical course, and response to treatments, and to extend the HPO to provide an even broader range of terms for the manifestations of complex disease, with the intention of providing a comprehensive resource for translational bioinformatics across the entire spectrum of human disease.

Supplemental Data

Supplemental Data include 7 figures and 41 tables and can be found with this article online at http://dx.doi.org/10.1016/j.ajhg.2015.05.020.

Acknowledgments

This work was supported by the Bundesministerium für Bildung und Forschung (project 0313911), the European Commission Seventh Framework Programme (FP7; grant 602300; SYBIL project), the Raine Clinician Research Fellowship (20140101), and the National Health and Medical Research Council of Australia (grant APP1055319, which is partnered with FP7 grant 305444). Oregon Health and Science University acknowledges the support of grant 1R24OD011883-01 from the NIH Office of the Director. T.G. was supported by an Australian Research Council Discovery Early Career Researcher Award (DE120100508). D.V. was supported in part by the BioMedBridges project funded by Research Infrastructures of the FP7 (grant 284209). H.P. was supported by European Molecular Biology Laboratory Core Funds. This work was supported by the director, Basic Energy Sciences, Office of Science, US Department of Energy under contract DE-AC02-05CH11231 and NIH contract 1R24OD011883-01. This document was prepared as an account of work sponsored by the US Government. While this document is believed to contain correct information, neither the US Government nor any agency thereof, nor the Regents of the University of California, nor any of their employees makes any warranty, express or implied, or assumes any legal responsibility for the accuracy, completeness, or usefulness of any information, apparatus, product, or process disclosed or represents that its use would not infringe privately owned rights. The views and opinions of authors expressed herein do not necessarily state or reflect those of the US Government or any agency thereof or the Regents of the University of California.

Received: March 20, 2015
Accepted: May 22, 2015
Published: June 25, 2015

Web Resources

The URLs for data presented herein are as follows:

Bio-LarK, http://bio-lark.org/
Common Disease Phenotype Browser, http://pubmed-browser.human-phenotype-ontology.org/
GWAS Central, http://help.gwascentral.org/info/data/data-sharing-statement/
Human Phenotype Ontology, http://www.human-phenotype-ontology.org/
Monarch Initiative, http://monarchinitiative.org
OMIM, http://omim.org/

References

1. Köhler, S., Doelken, S.C., Mungall, C.J., Bauer, S., Firth, H.V., Bailleul-Forestier, I., Black, G.C., Brown, D.L., Brudno, M., Campbell, J., et al. (2014). The Human Phenotype Ontology project: linking molecular biology and disease through phenotype data. Nucleic Acids Res. *42*, D966–D974.
2. Robinson, P.N., Köhler, S., Bauer, S., Seelow, D., Horn, D., and Mundlos, S. (2008). The Human Phenotype Ontology: a tool for annotating and analyzing human hereditary disease. Am. J. Hum. Genet. *83*, 610–615.
3. Köhler, S., Schulz, M.H., Krawitz, P., Bauer, S., Dölken, S., Ott, C.E., Mundlos, C., Horn, D., Mundlos, S., and Robinson, P.N. (2009). Clinical diagnostics in human genetics with semantic similarity searches in ontologies. Am. J. Hum. Genet. *85*, 457–464.
4. Bauer, S., Köhler, S., Schulz, M.H., and Robinson, P.N. (2012). Bayesian ontology querying for accurate and noise-tolerant semantic searches. Bioinformatics *28*, 2502–2508.
5. Soden, S.E., Saunders, C.J., Willig, L.K., Farrow, E.G., Smith, L.D., Petrikin, J.E., LePichon, J.B., Miller, N.A., Thiffault, I., Dinwiddie, D.L., et al. (2014). Effectiveness of exome and genome sequencing guided by acuity of illness for diagnosis of neurodevelopmental disorders. Sci Transl Med. *6*, 265ra16.
6. Robinson, P.N., Köhler, S., Oellrich, A., Wang, K., Mungall, C.J., Lewis, S.E., Washington, N., Bauer, S., Seelow, D., Krawitz, P., et al.; Sanger Mouse Genetics Project (2014). Improved exome prioritization of disease genes through cross-species phenotype comparison. Genome Res. *24*, 340–348.
7. Masino, A.J., Dechene, E.T., Dulik, M.C., Wilkens, A., Spinner, N.B., Krantz, I.D., Pennington, J.W., Robinson, P.N., and White, P.S. (2014). Clinical phenotype-based gene prioritization: an initial study using semantic similarity and the human phenotype ontology. BMC Bioinformatics *15*, 248.
8. Sifrim, A., Popovic, D., Tranchevent, L.C., Ardeshirdavani, A., Sakai, R., Konings, P., Vermeesch, J.R., Aerts, J., De Moor, B., and Moreau, Y. (2013). eXtasy: variant prioritization by genomic data fusion. Nat. Methods *10*, 1083–1084.
9. Javed, A., Agrawal, S., and Ng, P.C. (2014). Phen-Gen: combining phenotype and genotype to analyze rare disorders. Nat. Methods *11*, 935–937.
10. Singleton, M.V., Guthery, S.L., Voelkerding, K.V., Chen, K., Kennedy, B., Margraf, R.L., Durtschi, J., Eilbeck, K., Reese, M.G., Jorde, L.B., et al. (2014). Phevor combines multiple biomedical ontologies for accurate identification of disease-causing alleles in single individuals and small nuclear families. Am. J. Hum. Genet. *94*, 599–610.
11. Zemojtel, T., Kohler, S., Mackenroth, L., Jager, M., Hecht, J., Krawitz, P., Graul-Neumann, L., Doelken, S., Ehmke, N.,

Spielmann, M., et al. (2014). Effective diagnosis of genetic disease by computational phenotype analysis of the disease-associated genome. Sci Transl Med. *6*, 252ra123.
12. Gottlieb, A., Stein, G.Y., Ruppin, E., and Sharan, R. (2011). PREDICT: a method for inferring novel drug indications with application to personalized medicine. Mol. Syst. Biol. *7*, 496.
13. Bayés, A., van de Lagemaat, L.N., Collins, M.O., Croning, M.D., Whittle, I.R., Choudhary, J.S., and Grant, S.G. (2011). Characterization of the proteome, diseases and evolution of the human postsynaptic density. Nat. Neurosci. *14*, 19–21.
14. Castellano, S., Parra, G., Sánchez-Quinto, F.A., Racimo, F., Kuhlwilm, M., Kircher, M., Sawyer, S., Fu, Q., Heinze, A., Nickel, B., et al. (2014). Patterns of coding variation in the complete exomes of three Neandertals. Proc. Natl. Acad. Sci. USA *111*, 6666–6671.
15. Pinto, D., Delaby, E., Merico, D., Barbosa, M., Merikangas, A., Klei, L., Thiruvahindrapuram, B., Xu, X., Ziman, R., Wang, Z., et al. (2014). Convergence of genes and cellular pathways dysregulated in autism spectrum disorders. Am. J. Hum. Genet. *94*, 677–694.
16. Liakath-Ali, K., Vancollie, V.E., Heath, E., Smedley, D.P., Estabel, J., Sunter, D., Ditommaso, T., White, J.K., Ramirez-Solis, R., Smyth, I., et al. (2014). Novel skin phenotypes revealed by a genome-wide mouse reverse genetic screen. Nat. Commun. *5*, 3540.
17. Renkema, K.Y., Stokman, M.F., Giles, R.H., and Knoers, N.V. (2014). Next-generation sequencing for research and diagnostics in kidney disease. Nat. Rev. Nephrol. *10*, 433–444.
18. Sana, M.E., Spitaleri, A., Spiliotopoulos, D., Pezzoli, L., Preda, L., Musco, G., Ferrazzi, P., and Iascone, M. (2014). Identification of a novel de novo deletion in RAF1 associated with biventricular hypertrophy in Noonan syndrome. Am. J. Med. Genet. A. *164A*, 2069–2073.
19. Amberger, J.S., Bocchini, C.A., Schiettecatte, F., Scott, A.F., and Hamosh, A. (2015). OMIM.org: Online Mendelian Inheritance in Man (OMIM®), an online catalog of human genes and genetic disorders. Nucleic Acids Res. *43*, 789–798.
20. Kibbe, W.A., Arze, C., Felix, V., Mitraka, E., Bolton, E., Fu, G., Mungall, C.J., Binder, J.X., Malone, J., Vasant, D., et al. (2015). Disease Ontology 2015 update: an expanded and updated database of human diseases for linking biomedical knowledge through disease data. Nucleic Acids Res. *43*, D1071–D1078.
21. Petrovski, S., and Goldstein, D.B. (2014). Phenomics and the interpretation of personal genomes. Sci Transl Med. *6*, 254fs35.
22. Wright, C.F., Fitzgerald, T.W., Jones, W.D., Clayton, S., McRae, J.F., van Kogelenberg, M., King, D.A., Ambridge, K., Barrett, D.M., Bayzetinova, T., et al.; DDD study (2015). Genetic diagnosis of developmental disorders in the DDD study: a scalable analysis of genome-wide research data. Lancet *385*, 1305–1314.
23. Robinson, P.N., and Webber, C. (2014). Phenotype ontologies and cross-species analysis for translational research. PLoS Genet. *10*, e1004268.
24. Washington, N.L., Haendel, M.A., Mungall, C.J., Ashburner, M., Westerfield, M., and Lewis, S.E. (2009). Linking human diseases to animal models using ontology-based phenotype annotation. PLoS Biol. *7*, e1000247.
25. Mungall, C.J., Gkoutos, G.V., Smith, C.L., Haendel, M.A., Lewis, S.E., and Ashburner, M. (2010). Integrating phenotype ontologies across multiple species. Genome Biol. *11*, R2.
26. Haendel, M.A., Balhoff, J.P., Bastian, F.B., Blackburn, D.C., Blake, J.A., Bradford, Y., Comte, A., Dahdul, W.M., Dececchi, T.A., Druzinsky, R.E., et al. (2014). Unification of multi-species vertebrate anatomy ontologies for comparative biology in Uberon. J Biomed Semantics *5*, 21.
27. Hastings, J., de Matos, P., Dekker, A., Ennis, M., Harsha, B., Kale, N., Muthukrishnan, V., Owen, G., Turner, S., Williams, M., and Steinbeck, C. (2013). The ChEBI reference database and ontology for biologically relevant chemistry: enhancements for 2013. Nucleic Acids Res. *41*, D456–D463.
28. Smedley, D., Oellrich, A., Köhler, S., Ruef, B., Westerfield, M., Robinson, P., Lewis, S., and Mungall, C.; Sanger Mouse Genetics Project (2013). PhenoDigm: analyzing curated annotations to associate animal models with human diseases. Database (Oxford) *2013*, bat025.
29. Köhler, S., Doelken, S.C., Ruef, B.J., Bauer, S., Washington, N., Westerfield, M., Gkoutos, G., Schofield, P., Smedley, D., Lewis, S.E., et al. (2013). Construction and accessibility of a cross-species phenotype ontology along with gene annotations for biomedical research. F1000Res. *2*, 30.
30. Köhler, S., Bauer, S., Mungall, C.J., Carletti, G., Smith, C.L., Schofield, P., Gkoutos, G.V., and Robinson, P.N. (2011). Improving ontologies by automatic reasoning and evaluation of logical definitions. BMC Bioinformatics *12*, 418.
31. Bragin, E., Chatzimichali, E.A., Wright, C.F., Hurles, M.E., Firth, H.V., Bevan, A.P., and Swaminathan, G.J. (2014). DECIPHER: database for the interpretation of phenotype-linked plausibly pathogenic sequence and copy-number variation. Nucleic Acids Res. *42*, D993–D1000.
32. Vulto-van Silfhout, A.T., van Ravenswaaij, C.M., Hehir-Kwa, J.Y., Verwiel, E.T., Dirks, R., van Vooren, S., Schinzel, A., de Vries, B.B., and de Leeuw, N. (2013). An update on ECARUCA, the European Cytogeneticists Association Register of Unbalanced Chromosome Aberrations. Eur. J. Med. Genet. *56*, 471–474.
33. Beck, T., Hastings, R.K., Gollapudi, S., Free, R.C., and Brookes, A.J. (2014). GWAS Central: a comprehensive resource for the comparison and interrogation of genome-wide association studies. Eur. J. Hum. Genet. *22*, 949–952.
34. Li, M.J., Wang, P., Liu, X., Lim, E.L., Wang, Z., Yeager, M., Wong, M.P., Sham, P.C., Chanock, S.J., and Wang, J. (2012). GWASdb: a database for human genetic variants identified by genome-wide association studies. Nucleic Acids Res. *40*, D1047–D1054.
35. Welter, D., MacArthur, J., Morales, J., Burdett, T., Hall, P., Junkins, H., Klemm, A., Flicek, P., Manolio, T., Hindorff, L., and Parkinson, H. (2014). The NHGRI GWAS Catalog, a curated resource of SNP-trait associations. Nucleic Acids Res. *42*, D1001–D1006.
36. Biesecker, L.G., and Green, R.C. (2014). Diagnostic clinical genome and exome sequencing. N. Engl. J. Med. *370*, 2418–2425.
37. Chen, Y.C., Douville, C., Wang, C., Niknafs, N., Yeo, G., Beleva-Guthrie, V., Carter, H., Stenson, P.D., Cooper, D.N., Li, B., et al. (2014). A probabilistic model to predict clinical phenotypic traits from genome sequencing. PLoS Comput. Biol. *10*, e1003825.
38. Jonquet, C., Shah, N.H., and Musen, M.A. (2009). The open biomedical annotator. Summit on Translat Bioinforma *2009*, 56–60.
39. Campos, D., Matos, S., and Oliveira, J.L. (2013). A modular framework for biomedical concept recognition. BMC Bioinformatics *14*, 281.

40. Groza, T., Köhler, S., Doelken, S., Collier, N., Oellrich, A., Smedley, D., Couto, F.M., Baynam, G., Zankl, A., and Robinson, P.N. (2015). Automatic concept recognition using the human phenotype ontology reference and test suite corpora. Database (Oxford), 2015.
41. Robinson, P.N., and Bauer, S. (2011). Introduction to Biol.-Ontologies (Baton Rouge: CRC Press Inc.).
42. Demchak, B., Hull, T., Reich, M., Liefeld, T., Smoot, M., Ideker, T., and Mesirov, J.P. (2014). Cytoscape: the network visualization tool for GenomeSpace workflows. F1000Res. *3*, 151.
43. Maslov, S., and Sneppen, K. (2002). Specificity and stability in topology of protein networks. Science *296*, 910–913.
44. Zhang, W., Gamazon, E.R., Zhang, X., Konkashbaev, A., Liu, C., Szilágyi, K.L., Dolan, M.E., and Cox, N.J. (2015). SCAN database: facilitating integrative analyses of cytosine modification and expression QTL. Database (Oxford), 2015.
45. Schriml, L.M., Arze, C., Nadendla, S., Chang, Y.W., Mazaitis, M., Felix, V., Feng, G., and Kibbe, W.A. (2012). Disease Ontology: a backbone for disease semantic integration. Nucleic Acids Res. *40*, D940–D946.
46. Ripke, S., O'Dushlaine, C., Chambert, K., Moran, J.L., Kähler, A.K., Akterin, S., Bergen, S.E., Collins, A.L., Crowley, J.J., Fromer, M., et al.; Multicenter Genetic Studies of Schizophrenia Consortium; Psychosis Endophenotypes International Consortium; Wellcome Trust Case Control Consortium 2 (2013). Genome-wide association analysis identifies 13 new risk loci for schizophrenia. Nat. Genet. *45*, 1150–1159.
47. Stranger, B.E., Stahl, E.A., and Raj, T. (2011). Progress and promise of genome-wide association studies for human complex trait genetics. Genetics *187*, 367–383.
48. Doecke, J.D., Simms, L.A., Zhao, Z.Z., Huang, N., Hanigan, K., Krishnaprasad, K., Roberts, R.L., Andrews, J.M., Mahy, G., Bampton, P., et al. (2013). Genetic susceptibility in IBD: overlap between ulcerative colitis and Crohn's disease. Inflamm. Bowel Dis. *19*, 240–245.
49. Cross-Disorder Group of the Psychiatric Genomics Consortium (2013). Identification of risk loci with shared effects on five major psychiatric disorders: a genome-wide analysis. Lancet *381*, 1371–1379.
50. Richard-Miceli, C., and Criswell, L.A. (2012). Emerging patterns of genetic overlap across autoimmune disorders. Genome Med. *4*, 6.
51. Li, Y., Zhang, K., Chen, H., Sun, F., Xu, J., Wu, Z., Li, P., Zhang, L., Du, Y., Luan, H., et al. (2013). A genome-wide association study in Han Chinese identifies a susceptibility locus for primary Sjögren's syndrome at 7q11.23. Nat. Genet. *45*, 1361–1365.
52. Graham, R.R., Cotsapas, C., Davies, L., Hackett, R., Lessard, C.J., Leon, J.M., Burtt, N.P., Guiducci, C., Parkin, M., Gates, C., et al. (2008). Genetic variants near TNFAIP3 on 6q23 are associated with systemic lupus erythematosus. Nat. Genet. *40*, 1059–1061.
53. Zhou, X., Menche, J., Barabási, A.L., and Sharma, A. (2014). Human symptoms-disease network. Nat. Commun. *5*, 4212.
54. Blair, D.R., Lyttle, C.S., Mortensen, J.M., Bearden, C.F., Jensen, A.B., Khiabanian, H., Melamed, R., Rabadan, R., Bernstam, E.V., Brunak, S., et al. (2013). A nondegenerate code of deleterious variants in Mendelian loci contributes to complex disease risk. Cell *155*, 70–80.
55. Alzheimer's Disease Collaborative Group (1995). The structure of the presenilin 1 (S182) gene and identification of six novel mutations in early onset AD families. Nat. Genet. *11*, 219–222.
56. Lambert, J.C., Mann, D.M., Harris, J.M., Chartier-Harlin, M.C., Cumming, A., Coates, J., Lemmon, H., StClair, D., Iwatsubo, T., and Lendon, C. (2001). The -48 C/T polymorphism in the presenilin 1 promoter is associated with an increased risk of developing Alzheimer's disease and an increased Abeta load in brain. J. Med. Genet. *38*, 353–355.
57. Kathiresan, S., Willer, C.J., Peloso, G.M., Demissie, S., Musunuru, K., Schadt, E.E., Kaplan, L., Bennett, D., Li, Y., Tanaka, T., et al. (2009). Common variants at 30 loci contribute to polygenic dyslipidemia. Nat. Genet. *41*, 56–65.
58. Lusis, A.J., and Pajukanta, P. (2008). A treasure trove for lipoprotein biology. Nat. Genet. *40*, 129–130.
59. Stahl, E.A., Raychaudhuri, S., Remmers, E.F., Xie, G., Eyre, S., Thomson, B.P., Li, Y., Kurreeman, F.A., Zhernakova, A., Hinks, A., et al.; BIRAC Consortium; YEAR Consortium (2010). Genome-wide association study meta-analysis identifies seven new rheumatoid arthritis risk loci. Nat. Genet. *42*, 508–514.
60. Shi, Y., Zhao, H., Shi, Y., Cao, Y., Yang, D., Li, Z., Zhang, B., Liang, X., Li, T., Chen, J., et al. (2012). Genome-wide association study identifies eight new risk loci for polycystic ovary syndrome. Nat. Genet. *44*, 1020–1025.
61. Voight, B.F., Scott, L.J., Steinthorsdottir, V., Morris, A.P., Dina, C., Welch, R.P., Zeggini, E., Huth, C., Aulchenko, Y.S., Thorleifsson, G., et al.; MAGIC investigators; GIANT Consortium (2010). Twelve type 2 diabetes susceptibility loci identified through large-scale association analysis. Nat. Genet. *42*, 579–589.
62. Kraja, A.T., Vaidya, D., Pankow, J.S., Goodarzi, M.O., Assimes, T.L., Kullo, I.J., Sovio, U., Mathias, R.A., Sun, Y.V., Franceschini, N., et al. (2011). A bivariate genome-wide approach to metabolic syndrome: STAMPEED consortium. Diabetes *60*, 1329–1339.
63. Lill, C.M., Roehr, J.T., McQueen, M.B., Kavvoura, F.K., Bagade, S., Schjeide, B.M., Schjeide, L.M., Meissner, E., Zauft, U., Allen, N.C., et al.; 23andMe Genetic Epidemiology of Parkinson's Disease Consortium; International Parkinson's Disease Genomics Consortium; Parkinson's Disease GWAS Consortium; Wellcome Trust Case Control Consortium 2) (2012). Comprehensive research synopsis and systematic meta-analyses in Parkinson's disease genetics: The PDGene database. PLoS Genet. *8*, e1002548.
64. Kim, D.K., Cho, M.H., Hersh, C.P., Lomas, D.A., Miller, B.E., Kong, X., Bakke, P., Gulsvik, A., Agustí, A., Wouters, E., et al.; ECLIPSE, ICGN, and COPDGene Investigators (2012). Genome-wide association analysis of blood biomarkers in chronic obstructive pulmonary disease. Am. J. Respir. Crit. Care Med. *186*, 1238–1247.
65. Amberger, J., Bocchini, C., and Hamosh, A. (2011). A new face and new challenges for Online Mendelian Inheritance in Man (OMIM®). Hum. Mutat. *32*, 564–567.
66. Rath, A., Olry, A., Dhombres, F., Brandt, M.M., Urbero, B., and Ayme, S. (2012). Representation of rare diseases in health information systems: the Orphanet approach to serve a wide range of end users. Hum. Mutat. *33*, 803–808.
67. Oti, M., and Brunner, H.G. (2007). The modular nature of genetic diseases. Clin. Genet. *71*, 1–11.
68. Barabási, A.L. (2007). Network medicine—from obesity to the "diseasome". N. Engl. J. Med. *357*, 404–407.
69. Feldman, I., Rzhetsky, A., and Vitkup, D. (2008). Network properties of genes harboring inherited disease mutations. Proc. Natl. Acad. Sci. USA *105*, 4323–4328.
70. Vidal, M., Cusick, M.E., and Barabási, A.L. (2011). Interactome networks and human disease. Cell *144*, 986–998.

71. Dewey, F.E., Grove, M.E., Pan, C., Goldstein, B.A., Bernstein, J.A., Chaib, H., Merker, J.D., Goldfeder, R.L., Enns, G.M., David, S.P., et al. (2014). Clinical interpretation and implications of whole-genome sequencing. JAMA *311*, 1035–1045.
72. Hennekam, R.C., and Biesecker, L.G. (2012). Next-generation sequencing demands next-generation phenotyping. Hum. Mutat. *33*, 884–886.
73. Esplin, E.D., Oei, L., and Snyder, M.P. (2014). Personalized sequencing and the future of medicine: discovery, diagnosis and defeat of disease. Pharmacogenomics *15*, 1771–1790.
74. Voros, S., Maurovich-Horvat, P., Marvasty, I.B., Bansal, A.T., Barnes, M.R., Vazquez, G., Murray, S.S., Voros, V., Merkely, B., Brown, B.O., and Warnick, G.R. (2014). Precision phenotyping, panomics, and system-level bioinformatics to delineate complex biologies of atherosclerosis: rationale and design of the "Genetic Loci and the Burden of Atherosclerotic Lesions" study. J. Cardiovasc. Comput. Tomogr. *8*, 442–451.
75. Ball, M.P., Thakuria, J.V., Zaranek, A.W., Clegg, T., Rosenbaum, A.M., Wu, X., Angrist, M., Bhak, J., Bobe, J., Callow, M.J., et al. (2012). A public resource facilitating clinical use of genomes. Proc. Natl. Acad. Sci. USA *109*, 11920–11927.
76. Saunders, C.J., Miller, N.A., Soden, S.E., Dinwiddie, D.L., Noll, A., Alnadi, N.A., Andraws, N., Patterson, M.L., Krivohlavek, L.A., Fellis, J., et al. (2012). Rapid whole-genome sequencing for genetic disease diagnosis in neonatal intensive care units. Sci Transl Med. *4*, 154ra135.
77. Bell, C.J., Dinwiddie, D.L., Miller, N.A., Hateley, S.L., Ganusova, E.E., Mudge, J., Langley, R.J., Zhang, L., Lee, C.C., Schilkey, F.D., et al. (2011). Carrier testing for severe childhood recessive diseases by next-generation sequencing. Sci. Transl. Med. *3*, ra4.
78. Ellinor, P.T., Lunetta, K.L., Albert, C.M., Glazer, N.L., Ritchie, M.D., Smith, A.V., Arking, D.E., Müller-Nurasyid, M., Krijthe, B.P., Lubitz, S.A., et al. (2012). Meta-analysis identifies six new susceptibility loci for atrial fibrillation. Nat. Genet. *44*, 670–675.
79. Tsai, C.T., Hsieh, C.S., Chang, S.N., Chuang, E.Y., Juang, J.M., Lin, L.Y., Lai, L.P., Hwang, J.J., Chiang, F.T., and Lin, J.L. (2015). Next-generation sequencing of nine atrial fibrillation candidate genes identified novel de novo mutations in patients with extreme trait of atrial fibrillation. J. Med. Genet. *52*, 28–36.
80. Kohane, I.S. (2011). Using electronic health records to drive discovery in disease genomics. Nat. Rev. Genet. *12*, 417–428.
81. Denny, J.C., Bastarache, L., Ritchie, M.D., Carroll, R.J., Zink, R., Mosley, J.D., Field, J.R., Pulley, J.M., Ramirez, A.H., Bowton, E., et al. (2013). Systematic comparison of phenome-wide association study of electronic medical record data and genome-wide association study data. Nat. Biotechnol. *31*, 1102–1110.
82. Denny, J.C., Ritchie, M.D., Basford, M.A., Pulley, J.M., Bastarache, L., Brown-Gentry, K., Wang, D., Masys, D.R., Roden, D.M., and Crawford, D.C. (2010). PheWAS: demonstrating the feasibility of a phenome-wide scan to discover gene-disease associations. Bioinformatics *26*, 1205–1210.
83. Tatonetti, N.P., Denny, J.C., Murphy, S.N., Fernald, G.H., Krishnan, G., Castro, V., Yue, P., Tsao, P.S., Kohane, I., Roden, D.M., and Altman, R.B. (2011). Detecting drug interactions from adverse-event reports: interaction between paroxetine and pravastatin increases blood glucose levels. Clin. Pharmacol. Ther. *90*, 133–142.
84. Batet, M., Sánchez, D., and Valls, A. (2011). An ontology-based measure to compute semantic similarity in biomedicine. J. Biomed. Inform. *44*, 118–125.
85. Pesquita, C., Faria, D., Falcão, A.O., Lord, P., and Couto, F.M. (2009). Semantic similarity in biomedical ontologies. PLoS Comput. Biol. *5*, e1000443.

The American Journal of Human Genetics

Supplemental Data

The Human Phenotype Ontology:

Semantic Unification of Common and Rare Disease

Tudor Groza, Sebastian Köhler, Dawid Moldenhauer, Nicole Vasilevsky, Gareth Baynam, Tomasz Zemojtel, Lynn Marie Schriml, Warren Alden Kibbe, Paul N. Schofield, Tim Beck, Drashtti Vasant, Anthony J. Brookes, Andreas Zankl, Nicole L. Washington, Christopher J. Mungall, Suzanna E. Lewis, Melissa A. Haendel, Helen Parkinson, and Peter N. Robinson

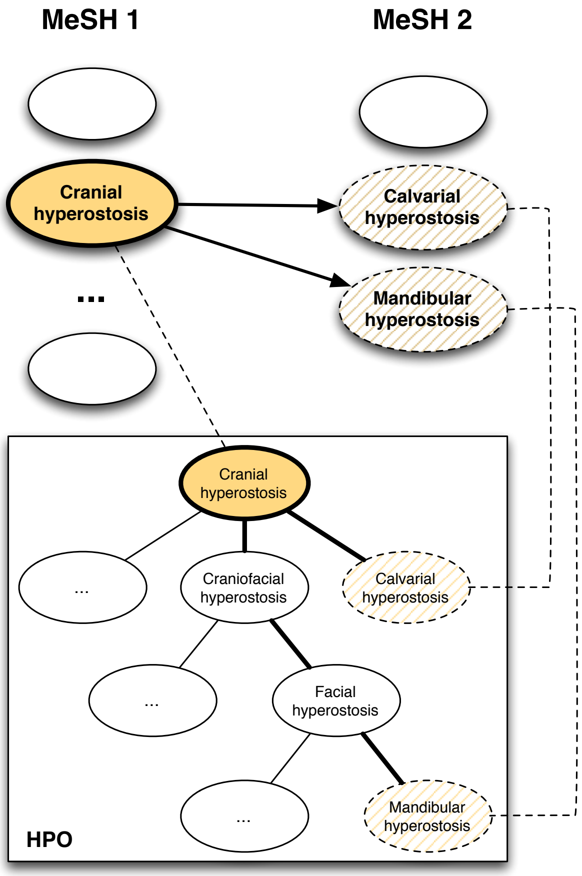

Figure S1. Adapted Jaccard measure. Example of using the HPO structure to compute the Jaccard index between the annotations of two different MeSH disorders. The standard Jaccard index is computed based on the assumption that the underlying data is represented as sets of symbolic elements. As a result, the index computes the ratio between the strict intersection and union of these elements. As opposed to symbolic elements, ontological concepts have the advantage of being structured in a logical hierarchy. This enables us to use the existing subsumption relation to quantify the degree of similarity between concepts, for example, by looking the path they share. This intrinsic similarity can also be exploited when computing the Jaccard index. Instead of performing the strict intersection and union of two sets of concepts, we considered a match also when two concepts share lineage – i.e., when one concept is an ancestor of the second. Such an example is presented in this figure, where *Cranial hyperostosis* is a parent of *Calvarial hyperostosis* and an ancestor of *Mandibular hyperostosis*, which leads to *Cranial hyperostosis* being the common ground for intersection between the two phenotype lists associated with MeSH 1 and MeSH 2.

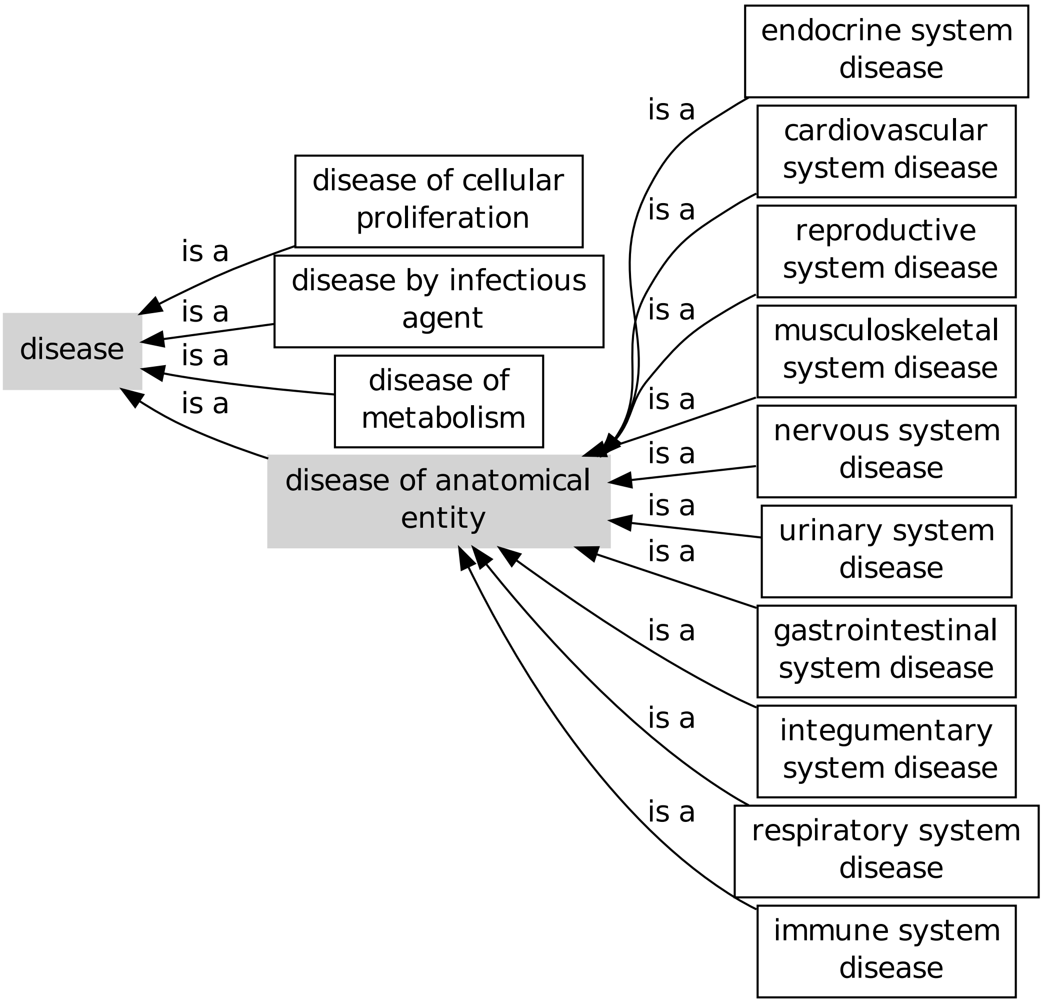

Figure S2. Disease Ontology Classes used for Clustering Common Disease. We chose 13 classes from the Disease Ontology [1] that represented standard categories used in internal medicine. Some class were excluded because they contained too few diseases (e.g., *thoracic disease*, n=13 diseases), or because they contained rare diseases (genetic disease or syndrome). The general classes "disease" and "disease of anatomical entity" (shown in light gray) were not included in the analysis.

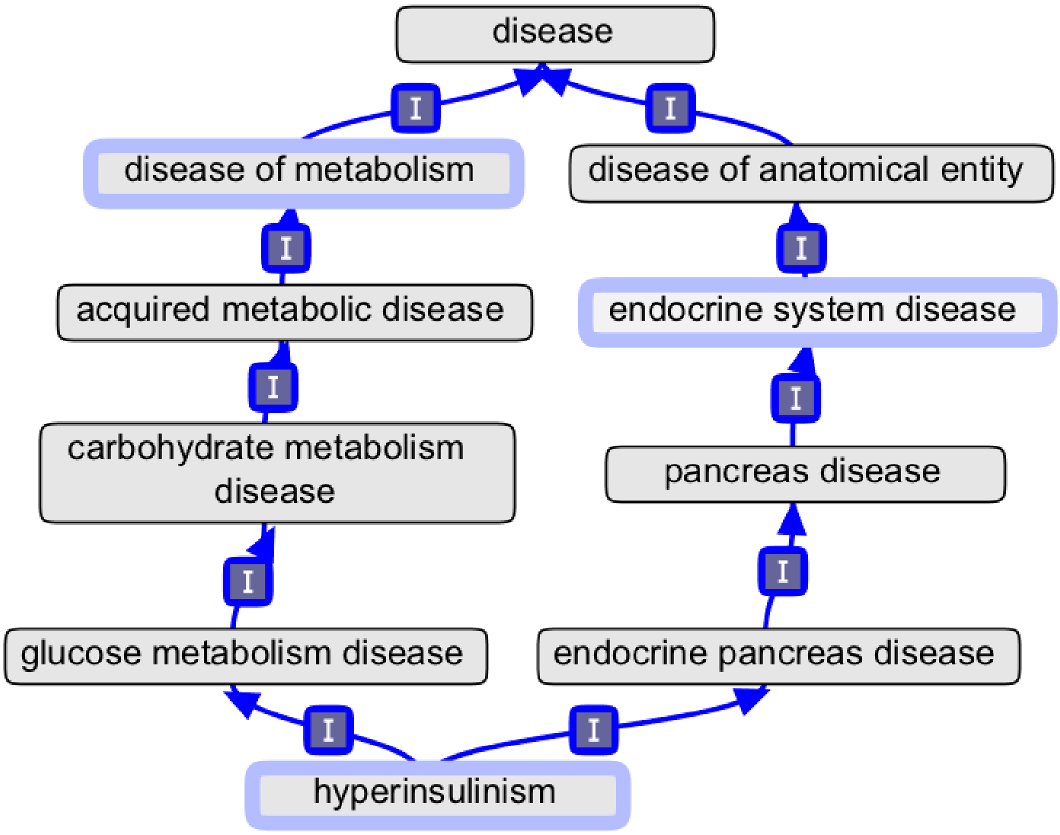

Figure S3. Diseases belonging to multiple Disease Ontology Classes. In some cases, individual diseases belong to more that one category. For instance, and as shown here, Hyperinsulinism is categorized as "disease of metabolism" and "endocrine system disease".

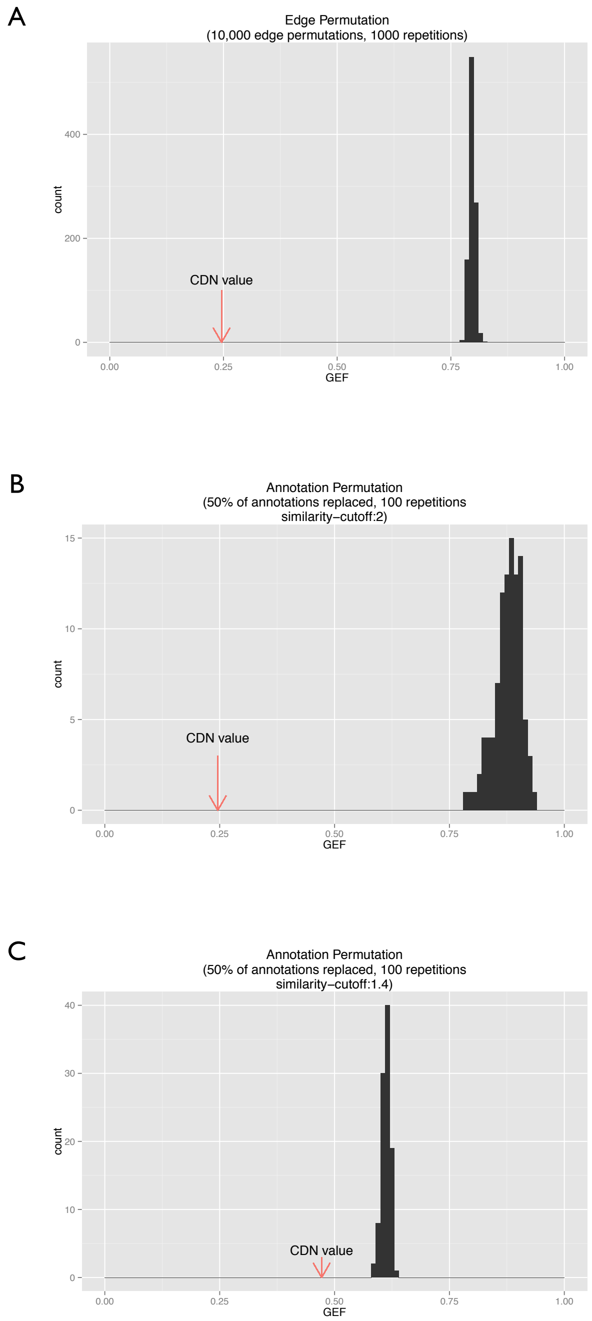

Figure S4. Grey edge fraction analysis of the CDN. Histograms of the *grey edge fraction* (GEF) values obtained by randomizations of the CDN. The red arrows show the GEF value of the original CDN. (**A**) Edge-randomized version of the original CDN (CDN-o) in which edges between diseases were randomly shuffled 10,000 times (CND-er). Not one of the randomized networks achieved as good a result as the value for CDN-o (empirical p value $< 10^{-4}$). (**B**) Annotation-randomized version of CDN-o in which 50% of the annotations were replaced by random annotations (threshold similarity value $simcut = 2.0$). (**C**) Similar to (B) but with threshold similarity value $simcut = 1.4$. In both simulations, not a single randomized network performed as well as the observed network, corresponding to an empirical p value of p <0.01.

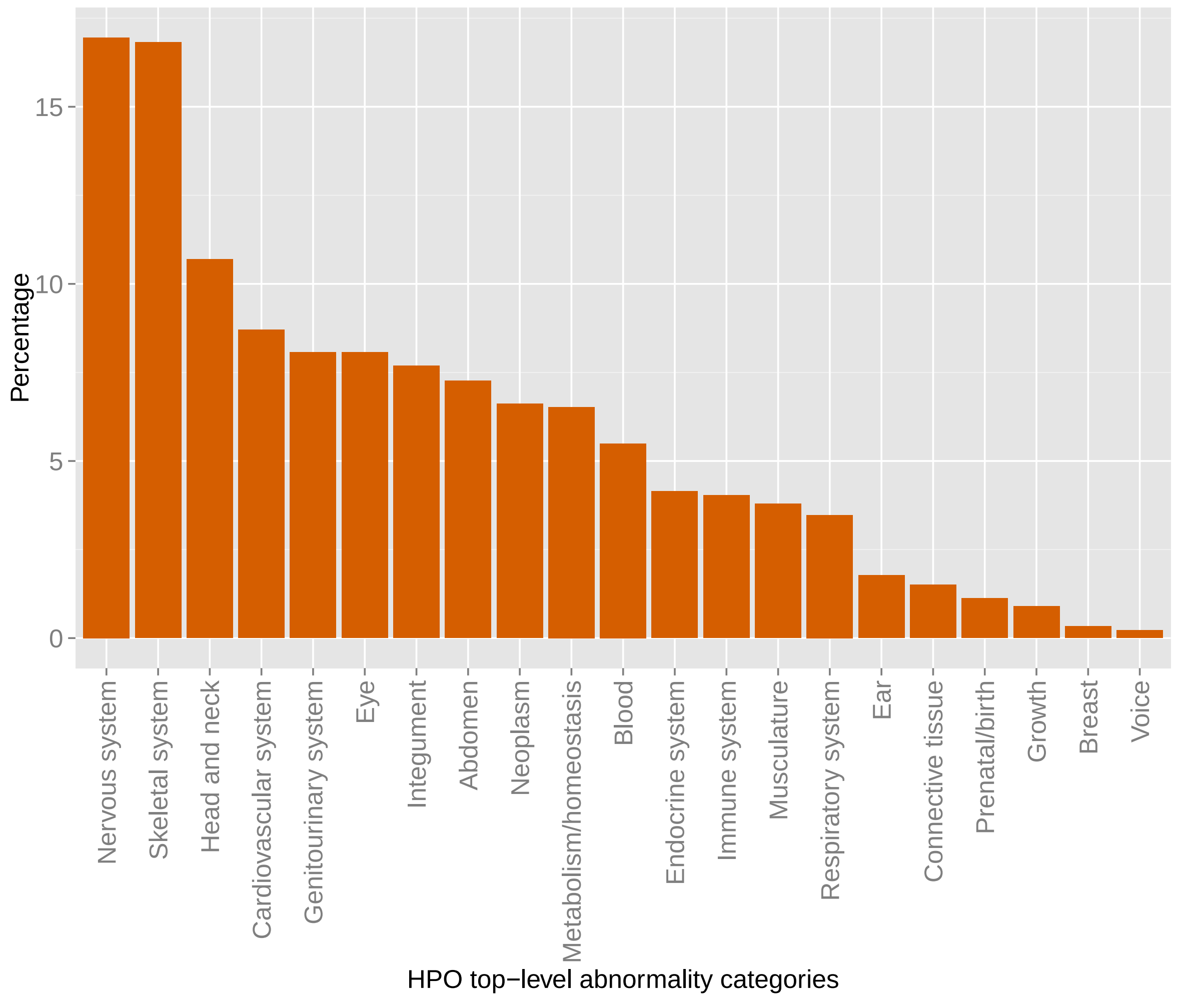

Figure S5. Distribution of top-level HPO Annotations among common diseases. The distribution of extracted HPO annotations according to the top level HPO concepts in the list of common MeSH disorders.

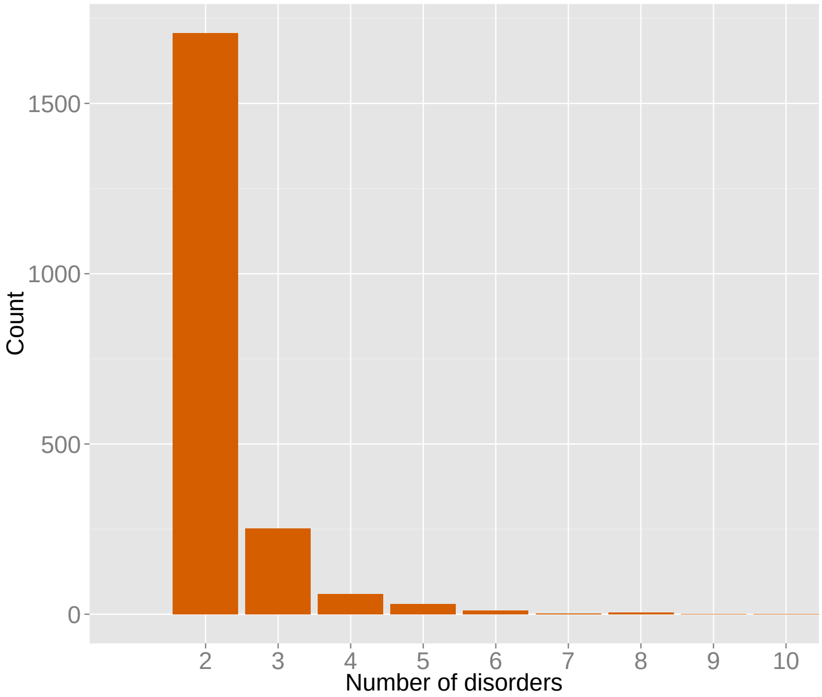

Figure S6. SNP Sharing. The figure shows the overall number of SNPs that are associated with multiple common diseases (among all 3145 common diseases examined in this project). Thus, the great majority of SNPs that are associated with multiple diseases are shared by a pair of diseases, but several hundred different SNPs are shared by three diseases, and so on. This analysis was based on a total of 16,152 SNPs listed in the GWAS Central database. Of these, 15,289 were associated with only a single disease. SNPs that were associated with two or more diseases are included in the bar chart shown in this Figure.

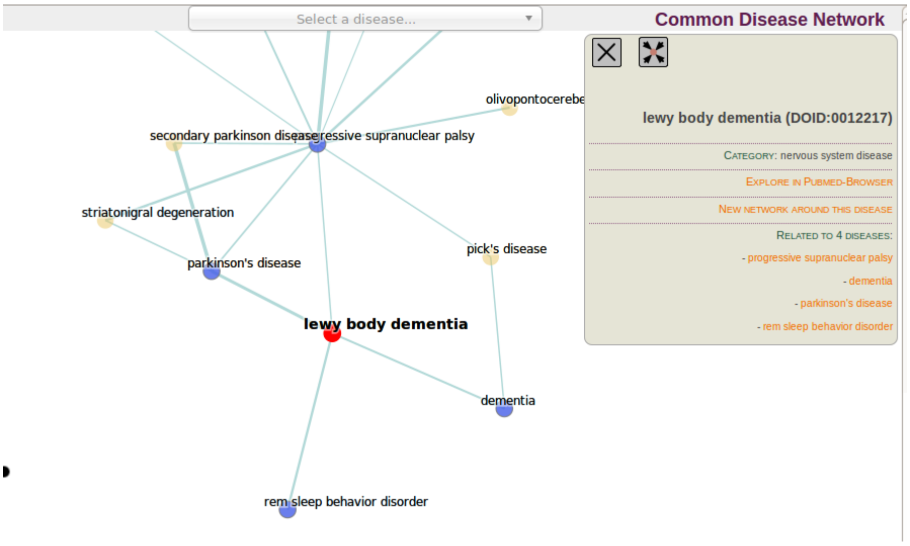

Figure S7. Common Disease Network (CDN) browser. Users can navigate throughout the network of common diseases that are interconnected by phenotypic similarity. To get to this page, enter the name of a disease (e.g., "Lewy body disease" into the search field at http://pubmed-browser.human-phenotype-ontology.org/, and choose the corresponding MeSH disease entry. The server will return a page entitled "Search Results" with a number of items. The disease page for "Lewy body disease" can now be reached via the link near the top of the Search Results page (the link next to the stethoscope symbol). This page, located at http://pubmed-browser.human-phenotype-ontology.org/#/mesh/D020961, contains a link "View Disease Network", that will open up the view shown in this Figure. Clicking on any of the nodes in the network will open up a window with additional information.

| | |

Table S1. Overview of HPO annotations for **Giant Cell Arteritis** that were derived by concept recognition in PubMed using BioLark. There were 68 true positives, 38 false positives, and 79 false negatives.

ID	Name	pmid	status	ID	Name	pmid	status
HP:0002633	Vasculitis	3160017	TP	HP:0002331	Headache (with pheochromocytoma)		FP
HP:0000572	Visual loss	10387327	TP	HP:0001138	Optic neuropathy	10387327	TP
HP:0003565	Elevated erythrocyte sedimentation rate	23795218	TP	HP:0001945	Fever	7242167	TP
HP:0005310	Large vessel vasculitis	7081559 15604899	TP	HP:0001824	Weight loss	21953306	TP
HP:0001297	Stroke	18640460	TP	HP:0002955	Granulomatosis		FP
HP:0001370	Rheumatoid arthritis		FP	HP:0000651	Diplopia	22119350	TP
HP:0001903	Anemia		FP	HP:0003326	Myalgia	6199905	TP
HP:0100576	Amaurosis fugax	9433866	TP	HP:0002617	Aneurysm	18634260	TP
HP:0004942	Aortic aneurysm	18021519	TP	HP:0002315	Headache	15384038	TP
HP:0003453	Antineutrophil antibody positivity		FP	HP:0100545	Arterial stenosis	22119350	TP
HP:0000969	Edema		FP	HP:0000822	Hypertension		FP
HP:0002621	Atherosclerosis		FP	HP:0001369	Arthritis	2652937 2068658	TP
HP:0002622	Dissecting aortic aneurysm	16766372	TP	HP:0009742	Stiff shoulders	23795218	TP
HP:0002725	Systemic lupus erythematosus		FP	HP:0002039	Anorexia	7242167 21953306	TP
HP:0001659	Aortic regurgitation	16829112	TP	HP:0100769	Synovitis	18640460 9448986	TP
HP:0001324	Muscle weakness	12861494	TP	HP:0000939	Osteoporosis		FP
HP:0002076	Migraine	955413 9747046	TP	HP:0011944	Small vessel vasculitis	21953306	TP
HP:0001894	Thrombocytosis	16543040 9222239	TP	HP:0011034	Amyloidosis	1128873	TP
HP:0009830	Peripheral neuropathy		FP	HP:0001658	Myocardial infarction	17546258	TP
HP:0100758	Gangrene	16344614	TP	HP:0004417	Intermittent claudication	11409140	TP
HP:0002634	Arteriosclerosis	1078327	TP	HP:0003365	Arthralgia of the hip	9458228	TP
HP:0001880	Eosinophilia		FP	HP:0000505	Visual impairment	9747046	TP
HP:0002647	Aortic dissection	16845847	TP	HP:0001289	Confusion		FP
HP:0100546	Carotid artery stenosis	10458090	TP	HP:0001123	Visual field defect	1243233	TP
HP:0009831	Mononeuropathy	2996347	TP	HP:0100661	Trigeminal neuralgia		FP
HP:0003155	Elevated alkaline phosphatase	1790639	TP	HP:0000718	Aggressive behavior		FP
HP:0002090	Pneumonia		FP	HP:0002326	Transient ischemic attack	3347337	TP
HP:0000622	Blurred vision	18052956	TP	HP:0004420	Arterial thrombosis	17546258	TP
HP:0000602	Ophthalmoplegia	1807820	TP	HP:0000819	Diabetes mellitus		FP
HP:0001287	Meningitis		FP	HP:0000648	Optic atrophy	7222706	TP
HP:0002631	Ascending aortic aneurysm	17310805	TP	HP:0003470	Paralysis		FP
HP:0000726	Dementia	1766283	TP	HP:0000501	Glaucoma		FP
HP:0003552	Muscle stiffness	1807819	TP	HP:0100653	Optic neuritis	8523347	TP
HP:0007686	Abnormal pupillary function	19733885 15590540	TP	HP:0000979	Purpura		FP
HP:0011134	Low-grade fever	17180298	TP	HP:0000518	Cataract		FP

continued on the next page

Table S1. Giant Cell Arteritis – continued

ID	Name	pmid	status	ID	Name	pmid	status
HP:0100324	Scleroderma		FP	HP:0007354	Amyotrophic lateral sclerosis		FP
HP:0005294	Arterial dissection		FP	HP:0000529	Progressive visual loss	17504884	TP
HP:0003881	Humeral sclerosis		FP	HP:0000100	Nephrotic syndrome	9058675	TP
HP:0011227	Elevated C-reactive protein level	23795218	TP	HP:0003207	Arterial calcification	9196863	TP
HP:0004950	Peripheral arterial disease	12835863 15710711	TP	HP:0000246	Sinusitis		FP
HP:0006824	Cranial nerve paralysis	22802376 6297074	TP	HP:0100749	Chest pain	16829112	TP
HP:0001269	Hemiparesis	12143952	TP	HP:0001271	Polyneuropathy	1402990	TP
HP:0100646	Thyroiditis		FP	HP:0000365	Hearing impairment	20233486	TP
HP:0002910	Elevated hepatic transaminases	7263904	TP	HP:0008653	Crescentic glomerulonephritis	12200821	TP
HP:0000820	Abnormality of the thyroid gland		FP	HP:0002829	Arthralgia	21627866	TP
HP:0001609	Hoarse voice	7863116	TP	HP:0002140	Ischemic stroke	20609853	TP
HP:0002758	Osteoarthritis		FP	HP:0001997	Gout		FP
HP:0000821	Hypothyroidism	1913003 8094625	TP	HP:0011510	Drusen	10150824	TP
HP:0000639	Nystagmus	2765284	TP	HP:0001145	Chorioretinopathy		FP
HP:0000836	Hyperthyroidism		FP	HP:0005113	Dilatation of the aortic arch		FP
HP:0100026	Arteriovenous malformation		FP	HP:0003095	Septic arthritis		FP
HP:0005111	Dilatation of the ascending aorta	-	TP	HP:0004933	Ascending aortic dissection	19049773 21521678	TP
HP:0005059	arthralgia/arthritis		FP	HP:0000495	Recurrent corneal erosions		FP
HP:0005318	Cerebral vasculitis	8033943	FN	HP:0002616	Aortic root dilatation	2209142 4030882	FN
HP:0001955	Unexplained fevers	218291	FN	HP:0005200	Retroperitoneal fibrosis	24885445	FN
HP:0002367	Visual hallucinations	11550973	FN	HP:0001085	Papilledema	131544	FN
HP:0002301	Hemiplegia	501373	FN	HP:0001260	Dysarthria	9745245	FN
HP:0002113	Pulmonary infiltrates	8777858 2052510	FN	HP:0009763	Limb pain	2655505	FN
HP:0000520	Proptosis	18052956	FN	HP:0003613	Antiphospholipid antibody positivity	11503135	FN
HP:0001701	Pericarditis	17335942	FN	HP:0001698	Pericardial effusion	17031245	FN
HP:0003401	Paresthesia	20609853	FN	HP:0000508	Ptosis	12143952	FN
HP:0004953	Abdominal aortic aneurysm	13679546	FN	HP:0000554	Uveitis	17020003	FN
HP:0004944	Cerebral aneurysm	17961913	FN	HP:0001681	Angina pectoris	2759121	FN
HP:0003198	Myopathy	9739500	FN	HP:0001279	Syncope	6380900	FN
HP:0002138	Subarachnoid hemorrhage	1990421	FN	HP:0002321	Vertigo	3230240	FN
HP:0100584	Endocarditis	16859594	FN	HP:0002202	Pleural effusion	20400261	FN
HP:0001724	Aortic dilatation	7361287	FN	HP:0001974	Leukocytosis	16148728	FN
HP:0001907	Thromboembolism	19811309	FN	HP:0003774	Stage 5 chronic kidney disease	15384038 10620555	FN
HP:0002637	Cerebral ischemia	20626748	FN	HP:0002527	Falls	16859597	FN
HP:0001635	Congestive heart failure	955413 17269602	FN	HP:0000083	Renal insufficiency	1489011	FN
HP:0002960	Autoimmunity	7581345	FN	HP:0000618	Blindness	21953306	FN
HP:0000716	Depression	1807819	FN	HP:0000543	Optic disc pallor	17020004	FN

continued on the next page

Table S1. Giant Cell Arteritis – continued

ID	Name	pmid	status	ID	Name	pmid	status
HP:0200029	Vasculitis in the skin	14528512	FN	HP:0100534	Episcleritis	1012996	FN
HP:0000597	Ophthalmoparesis	3057903	FN	HP:0001342	Cerebral hemorrhage	9385928	FN
HP:0006530	Interstitial pulmonary disease	8745756	FN	HP:0010783	Erythema	3675013	FN
HP:0000093	Proteinuria	2729757	FN	HP:0002027	Abdominal pain	18204370	FN
HP:0100543	Cognitive impairment	2330100	FN	HP:0100963	Hyperesthesia	19497602	FN
HP:0100704	Cortical visual impairment	3057903	FN	HP:0100532	Scleritis	17020003	FN
HP:0001965	Abnormality of the scalp	21953306 17476617	FN	HP:0000282	Facial edema	8689287	FN
HP:0002907	Microscopic hematuria	11103864	FN	HP:0002344	Progressive neurologic deterioration	10578411 19592058	FN
HP:0010628	Facial palsy	10962818 9621267	FN	HP:0000541	Retinal detachment	21563451	FN
HP:0002015	Dysphagia	17509668	FN	HP:0001291	Abnormality of the cranial nerves	9310116	FN
HP:0005291	Inflammatory arteriopathy	20609853	FN	HP:0011353	Arterial intimal fibrosis	1807817 11466252	FN
HP:0000206	Glossitis	3320647	FN	HP:0000603	Central scotoma	7800356	FN
HP:0001917	Renal amyloidosis	9058675 11758014	FN	HP:0010532	Paroxysmal vertigo	15280720	FN
HP:0000211	Trismus	16859591	FN	HP:0003281	Increased serum ferritin	16543040	FN
HP:0007863	Retinal lesions	-	FN	HP:0003324	Generalized muscle weakness	17340046	FN
HP:0001605	Vocal cord paralysis	16854506	FN	HP:0000573	Retinal hemorrhage	12692357 11130757	FN
HP:0002318	Cervical myelopathy	9367971	FN	HP:0002102	Pleuritis	20400261	FN
HP:0000615	Abnormality of the pupil	14552190	FN	HP:0000790	Hematuria	2729757	FN
HP:0002625	Deep venous thrombosis	11503135	FN	HP:0001679	Abnormality of the aorta	-	FN
HP:0005794	Arterial disease of legs	-	FN	HP:0011477	Upbeat nystagmus	6703989	FN
HP:0003547	Shoulder girdle muscle weakness	-	FN				

Table S2. Overview of HPO annotations for **Cholesterol Embolism** that were derived by concept recognition in PubMed using BioLark. There were 18 true positives, 27 false positives, and 34 false negatives.

ID	Name	pmid	status	ID	Name	pmid	status
HP:0000965	Cutis marmorata	15240205 22588660	TP	HP:0001919	Acute kidney injury	21841332	TP
HP:0002621	Atherosclerosis		FP	HP:0000083	Renal insufficiency	11358047	TP
HP:0001880	Eosinophilia	17726656	TP	HP:0000822	Hypertension	8541013	TP
HP:0001658	Myocardial infarction		FP	HP:0003774	Stage 5 chronic kidney disease	15705188	TP
HP:0001297	Stroke		FP	HP:0002633	Vasculitis	9217601	TP
HP:0003259	Elevated serum creatinine	17634712	TP	HP:0001920	Renal artery stenosis		FP
HP:0000961	Cyanosis		FP	HP:0004953	Abdominal aortic aneurysm		FP
HP:0000093	Proteinuria	9404775	TP	HP:0004950	Peripheral arterial disease		FP
HP:0003326	Myalgia	16940713	TP	HP:0002617	Aneurysm		FP
HP:0002140	Ischemic stroke		FP	HP:0100546	Carotid artery stenosis		FP
HP:0000112	Nephropathy		FP	HP:0000819	Diabetes mellitus		FP
HP:0002027	Abdominal pain	12873565	TP	HP:0002239	Gastrointestinal hemorrhage	9404775	TP
HP:0001677	Coronary artery disease		FP	HP:0004942	Aortic aneurysm		FP
HP:0001907	Thromboembolism		FP	HP:0000979	Purpura	17695780	TP
HP:0002586	Peritonitis		FP	HP:0001635	Congestive heart failure		FP
HP:0003077	Hyperlipidemia		FP	HP:0009763	Limb pain	17695780	TP
HP:0005110	Atrial fibrillation		FP	HP:0002326	Transient ischemic attack	11419038	TP
HP:0009741	Nephrosclerosis		FP	HP:0004417	Intermittent claudication		FP
HP:0001945	Fever	15705188	TP	HP:0100598	Pulmonary edema	12238276	TP
HP:0003124	Hypercholesterolemia		FP	HP:0002635	Atheromatosis		FP
HP:0002583	Colitis	8669792	TP	HP:0002634	Arteriosclerosis		FP
HP:0001888	Lymphopenia		FP	HP:0004406	Spontaneous recurrent epistaxis		FP
HP:0001899	Increased hematocrit		FP	HP:0100758	Gangrene	21841332	FN
HP:0001082	Cholecystitis	10429867	FN	HP:0002014	Diarrhea	12873565	FN
HP:0001733	Pancreatitis	9445132	FN	HP:0000790	Hematuria	16430035 11171470	FN
HP:0001735	Acute pancreatitis	11100174	FN	HP:0001289	Confusion	18072326	FN
HP:0001824	Weight loss	21993354	FN	HP:0100576	Amaurosis fugax	21993354	FN
HP:0002573	Hematochezia	17277861 15494680	FN	HP:0000100	Nephrotic syndrome	9041208	FN
HP:0002157	Azotemia	20453403	FN	HP:0000099	Glomerulonephritis	18651554 12324923 9041208	FN
HP:0001063	Acrocyanosis	7727880 9235104	FN	HP:0006846	Acute encephalopathy	17229746	FN
HP:0011227	Elevated C-reactive protein level	12875753	FN	HP:0008682	Acute tubular necrosis	12649545	FN
HP:0200042	Skin ulcer	21946763	FN	HP:0100614	Myositis	11394629	FN
HP:0000096	Glomerulosclerosis	16773802	FN	HP:0001269	Hemiparesis	12040986	FN
HP:0010550	Paraplegia	15515703	FN	HP:0001324	Muscle weakness	8121874	FN
HP:0004325	Decreased body weight	21993354	FN	HP:0003138	Increased blood urea nitrogen (BUN)	18840042	FN
HP:0004713	Reversible renal failure	12187114	FN	HP:0007123	Subcortical dementia	15465102	FN
HP:0002913	Myoglobinuria	18480661	FN	HP:0003323	Progressive muscle weakness	19774498	FN
HP:0100732	Pancreatic fibrosis	9445132	FN	HP:0002907	Microscopic hematuria	22991843	FN

continued on the next page

Table S2. Cholesterol Embolism – continued

ID	Name	pmid	status	ID	Name	pmid	status
HP:0003565	Elevated erythrocyte sedimentation rate	21993354	FN	HP:0100282	Acute colitis	7806835	FN
HP:0001974	Leukocytosis	21993354	FN				

| | |

Table S3. Overview of HPO annotations for **Postphlebitic Syndrome** that were derived by concept recognition in PubMed using BioLark. There were 14 true positives, 3 false positives, and 11 false negatives.

ID	Name	pmid	status	ID	Name	pmid	status
HP:0002625	Deep venous thrombosis	25246013	TP	HP:0005293	Venous insufficiency	25246013	TP
HP:0002204	Pulmonary embolism	19741190	TP	HP:0004936	Venous thrombosis	19741190	TP
HP:0000969	Edema	19741190	TP	HP:0002619	Varicose veins	10886478	TP
HP:0004831	Recurrent thromboembolism	16634738	TP	HP:0001907	Thromboembolism	19741190	TP
HP:0200042	Skin ulcer	3209615	TP	HP:0001004	Lymphedema		FP
HP:0004325	Decreased body weight		FP	HP:0004418	Thrombophlebitis	9377251	TP
HP:0004419	Recurrent thrombophlebitis	20870815	TP	HP:0002624	Venous abnormality	3073400	TP
HP:0010834	Trophic changes related to pain	10378331	TP	HP:0010741	Edema of the lower limbs	3275807	TP
HP:0100695	Lipedema		FP	HP:0003394	Muscle cramps	8059211	FN
HP:0001000	Abnormality of skin pigmentation	19741190	FN	HP:0004947	Arteriovenous fistula	1799229 1285578	FN
HP:0004417	Intermittent claudication	1496032	FN	HP:0009763	Limb pain	14693168	FN
HP:0000989	Pruritus	10886478	FN	HP:0001785	Ankle swelling	2662673	FN
HP:0004850	Recurrent deep vein thrombosis	10886478	FN	HP:0003401	Paresthesia	2130425	FN
HP:0010783	Erythema	2695441	FN	HP:0001977	Abnormal thrombosis	-	FN

Table S4. Overview of HPO annotations for **Pernicious Anemia** that were derived by concept recognition in PubMed using BioLark. There were 17 true positives, 40 false positives, and 7 false negatives.

ID	Name	pmid	status	ID	Name	pmid	status
HP:0001903	Anemia		FP	HP:0005263	Gastritis		FP
HP:0002960	Autoimmunity	4890425	TP	HP:0002024	Malabsorption		FP
HP:0001889	Megaloblastic anemia	11005035	TP	HP:0100570	Carcinoid		FP
HP:0002582	Chronic atrophic gastritis		FP	HP:0001980	Megaloblastic bone marrow	3332113	TP
HP:0001045	Vitiligo		FP	HP:0002592	Gastric ulcer		FP
HP:0000820	Abnormality of the thyroid gland		FP	HP:0002588	Duodenal ulcer		FP
HP:0001972	Macrocytic anemia	11005035	TP	HP:0008207	Primary adrenal insufficiency		FP
HP:0100646	Thyroiditis		FP	HP:0001891	Iron deficiency anemia		FP
HP:0009830	Peripheral neuropathy	19689867	TP	HP:0002044	Zollinger-Ellison syndrome		FP
HP:0005231	Chronic gastritis		FP	HP:0005219	Absence of intrinsic factor	3332113	TP
HP:0000819	Diabetes mellitus		FP	HP:0000872	Hashimoto thyroiditis		FP
HP:0000821	Hypothyroidism		FP	HP:0001876	Pancytopenia	18622120	TP
HP:0004313	Hypogammaglobulinemia	3544232	TP	HP:0004395	Malnutrition		FP
HP:0001890	Autoimmune hemolytic anemia		FP	HP:0100651	Type I diabetes mellitus		FP
HP:0005202	Helicobacter pylori infection		FP	HP:0100647	Graves disease		FP
HP:0002725	Systemic lupus erythematosus		FP	HP:0002196	Myelopathy	6166087 435137	TP
HP:0002527	Falls		FP	HP:0001878	Hemolytic anemia		FP
HP:0002608	Celiac disease		FP	HP:0001370	Rheumatoid arthritis		FP
HP:0002835	Aspiration		FP	HP:0001324	Muscle weakness		FP
HP:0000726	Dementia	10367704	TP	HP:0000206	Glossitis	18125798	TP
HP:0005518	Erythrocyte macrocytosis	3332113	TP	HP:0001973	Autoimmune thrombocytopenia		FP
HP:0010972	Anemia of inadequate production	857850	TP	HP:0003881	Humeral sclerosis		FP
HP:0003473	Fatigable weakness		FP	HP:0006753	Neoplasm of the stomach	23216458	TP
HP:0001251	Ataxia	1648656	TP	HP:0000836	Hyperthyroidism		FP
HP:0002721	Immunodeficiency		FP	HP:0002863	Myelodysplasia		FP
HP:0001508	Failure to thrive	20404749 1432418 18454811	TP	HP:0001733	Pancreatitis		FP
HP:0001873	Thrombocytopenia		FP	HP:0003401	Paresthesia	18153465	TP
HP:0011273	Anisocytosis		FP	HP:0100751	Esophageal neoplasm		FP
HP:0002571	Achalasia		FP	HP:0001138	Optic neuropathy	15587778	FN
HP:0001271	Polyneuropathy	12975298	FN	HP:0000709	Psychosis	6849439 20807971	FN
HP:0002403	Positive Romberg sign	9658486	FN	HP:0010871	Sensory ataxia	11275463	FN
HP:0003487	Babinski sign	11503492	FN	HP:0004340	Abnormality of vitamin B metabolism	265681	FN

Table S5. Overview of HPO annotations for **Diabetic Ketoacidosis** that were derived by concept recognition in PubMed using BioLark. There were 29 true positives, 40 false positives, and 62 false negatives.

ID	Name	pmid	status	ID	Name	pmid	status
HP:0001953	Diabetic ketoacidosis	6281619	TP	HP:0001993	Ketoacidosis	-	TP
HP:0003074	Hyperglycemia	15460517	TP	HP:0001946	Ketosis	-	TP
HP:0001943	Hypoglycemia		FP	HP:0001941	Acidosis	-	TP
HP:0005974	Episodic ketoacidosis		FP	HP:0100651	Type I diabetes mellitus		FP
HP:0001942	Metabolic acidosis	-	TP	HP:0001259	Coma	1788182	TP
HP:0002181	Cerebral edema	20420811	TP	HP:0002919	Ketonuria	23357396	TP
HP:0001944	Dehydration	17632987	TP	HP:0005979	Metabolic ketoacidosis	15095958	TP
HP:0000819	Diabetes mellitus		FP	HP:0003128	Lactic acidosis		FP
HP:0001733	Pancreatitis		FP	HP:0000855	Insulin resistance		FP
HP:0002900	Hypokalemia	16191494	TP	HP:0002527	Falls		FP
HP:0001513	Obesity		FP	HP:0001959	Polydipsia	23075084	TP
HP:0003076	Glycosuria	6331271	TP	HP:0001824	Weight loss		FP
HP:0002013	Vomiting	22267622	TP	HP:0002027	Abdominal pain	22267622	TP
HP:0002148	Hypophosphatemia		FP	HP:0001735	Acute pancreatitis		FP
HP:0000488	Retinopathy		FP	HP:0000718	Aggressive behavior		FP
HP:0002960	Autoimmunity		FP	HP:0009830	Peripheral neuropathy		FP
HP:0005978	Type II diabetes mellitus		FP	HP:0000112	Nephropathy		FP
HP:0001988	Recurrent hypoglycemia		FP	HP:0009800	Maternal diabetes		FP
HP:0100806	Sepsis	9822196	TP	HP:0100753	Schizophrenia		FP
HP:0002017	Nausea and vomiting	-	TP	HP:0002153	Hyperkalemia	20420664	TP
HP:0001658	Myocardial infarction	822609	TP	HP:0001254	Lethargy	22267622	TP
HP:0100598	Pulmonary edema	6767583	TP	HP:0001325	Hypoglycemic coma		FP
HP:0001950	Respiratory alkalosis		FP	HP:0000083	Renal insufficiency		FP
HP:0003201	Rhabdomyolysis	20397738	TP	HP:0002098	Respiratory distress		FP
HP:0001250	Seizures	15960181	TP	HP:0001673	Tachycardia (with pheochromocytoma)		FP
HP:0011106	Hypovolemia	23283273	TP	HP:0002615	Hypotension	23283273	TP
HP:0001397	Hepatic steatosis		FP	HP:0002093	Respiratory insufficiency		FP
HP:0000822	Hypertension	23283273	TP	HP:0001297	Stroke		FP
HP:0002344	Progressive neurologic deterioration		FP	HP:0001289	Confusion	22267622	TP
HP:0001324	Muscle weakness		FP	HP:0000246	Sinusitis		FP
HP:0004395	Malnutrition		FP	HP:0000831	Insulin-resistant diabetes mellitus		FP
HP:0002039	Anorexia		FP	HP:0011947	Respiratory tract infection		FP
HP:0002719	Recurrent infections		FP	HP:0004904	Maturity-onset diabetes of the young		FP
HP:0002018	Nausea	18520103	TP	HP:0006543	Cardiorespiratory arrest		FP
HP:0002574	Episodic abdominal pain		FP	HP:0004918	hyperchloremic metabolic acidosis	10030094	FN
HP:0001986	Hypertonic dehydration	822694	FN	HP:0004900	Severe lactic acidosis	16791396	FN
HP:0001995	Hyperchloremic acidosis	1826776	FN	HP:0002151	Increased serum lactate	7885271	FN
HP:0008942	Acute rhabdomyolysis	14655521	FN	HP:0006279	Beta-cell dysfunction	17599861	FN
HP:0003228	Hypernatremia	8696061	FN	HP:0002917	Hypomagnesemia	10224681	FN
HP:0002789	Tachypnea	19106720	FN	HP:0005305	Cerebral venous thrombosis	21244475	FN
HP:0000103	Polyuria	22104427	FN	HP:0002516	Increased intracranial pressure	3150280	FN
HP:0002329	Drowsiness	16489969	FN	HP:0002072	Chorea	21632136	FN
HP:0007185	Loss of consciousness	17185803	FN	HP:0100537	Fasciitis	6418495	FN

continued on the next page

Table S5. Diabetic Ketoacidosis – continued

ID	Name	pmid	status	ID	Name	pmid	status
HP:0001278	Orthostatic hypotension	6798666	FN	HP:0002902	Hyponatremia	814023	FN
HP:0002883	Hyperventilation	15982426	FN	HP:0005521	Disseminated intravascular coagulation	825399	FN
HP:0003259	Elevated serum creatinine	6441297	FN	HP:0100724	Hypercoagulability	17380929	FN
HP:0002155	Hypertriglyceridemia	19667310	FN	HP:0000975	Hyperhidrosis	22356444	FN
HP:0002625	Deep venous thrombosis	22356837	FN	HP:0001919	Acute kidney injury	12708572	FN
HP:0001974	Leukocytosis	821284	FN	HP:0003077	Hyperlipidemia	22233951	FN
HP:0000093	Proteinuria	6420364	FN	HP:0001695	Cardiac arrest	12748130	FN
HP:0001298	Encephalopathy	20420811	FN	HP:0001907	Thromboembolism	19542020	FN
HP:0002014	Diarrhea	21551959	FN	HP:0002637	Cerebral ischemia	23515102	FN
HP:0011675	Arrhythmia	21316179	FN	HP:0001939	Abnormality of metabolism/homeostasis	-	FN
HP:0000017	Nocturia	21381577	FN	HP:0001342	Cerebral hemorrhage	18039811	FN
HP:0002157	Azotemia	22391852	FN	HP:0003256	Abnormality of the coagulation cascade	-	FN
HP:0002239	Gastrointestinal hemorrhage	8565740	FN	HP:0004936	Venous thrombosis	-	FN
HP:0003111	Abnormality of ion homeostasis	-	FN	HP:0011458	Abdominal symptom	-	FN
HP:0004420	Arterial thrombosis	16570569	FN	HP:0000737	Irritability	8685764	FN
HP:0002170	Intracranial hemorrhage	1698585	FN	HP:0004372	Reduced consciousness/confusion	-	FN
HP:0006846	Acute encephalopathy	403389	FN	HP:0000217	Xerostomia	14575617	FN
HP:0002315	Headache	23772471	FN	HP:0008279	Transient hyperlipidemia	11051350	FN
HP:0003113	Hypochloremia	19606251	FN	HP:0002641	Peripheral thrombosis	17315523	FN
HP:0100812	Halitosis	-	FN	HP:0002905	Hyperphosphatemia	3933341	FN
HP:0000805	Enuresis	22145453	FN	HP:0001262	Somnolence	8844491	FN
HP:0000713	Agitation	16489969	FN	HP:0002149	Hyperuricemia	14483098	FN
HP:0004360	Abnormality of acid-base homeostasis	-	FN				

Table S6. Overview of HPO annotations for **Hemochromatosis** that were derived by concept recognition in PubMed using BioLark. There were 31 true positives, 68 false positives, and 14 false negatives.

ID	Name	pmid	status	ID	Name	pmid	status
HP:0001394	Cirrhosis	9867745	TP	HP:0003281	Increased serum ferritin	9422115 11531973	TP
HP:0001903	Anemia		FP	HP:0003040	Arthropathy	1788814	TP
HP:0001402	Hepatocellular carcinoma	6282722 12828961	TP	HP:0005560	Imbalanced hemoglobin synthesis		FP
HP:0001395	Hepatic fibrosis	11832443	TP	HP:0000135	Hypogonadism		FP
HP:0000819	Diabetes mellitus		FP	HP:0001399	Hepatic failure		FP
HP:0001638	Cardiomyopathy	9867745	TP	HP:0001397	Hepatic steatosis		FP
HP:0003452	Increased serum iron	19477142	TP	HP:0001635	Congestive heart failure		FP
HP:0001000	Abnormality of skin pigmentation		FP	HP:0001369	Arthritis		FP
HP:0002910	Elevated hepatic transaminases	8094554	TP	HP:0001891	Iron deficiency anemia		FP
HP:0000934	Chondrocalcinosis	12117686	TP	HP:0006562	Viral hepatitis		FP
HP:0009824	Upper limb undergrowth		FP	HP:0000802	Impotence	19477142	TP
HP:0002896	Neoplasm of the liver		FP	HP:0003365	Arthralgia of the hip		FP
HP:0002240	Hepatomegaly	24343468	TP	HP:0000855	Insulin resistance		FP
HP:0001410	Decreased liver function	17606206	TP	HP:0010972	Anemia of inadequate production		FP
HP:0001733	Pancreatitis		FP	HP:0001924	Sideroblastic anemia	4017031	TP
HP:0007354	Amyotrophic lateral sclerosis		FP	HP:0002613	Biliary cirrhosis		FP
HP:0100646	Thyroiditis		FP	HP:0000044	Hypogonadotrophic hypogonadism	9867745	TP
HP:0001644	Dilated cardiomyopathy	6418103	TP	HP:0002829	Arthralgia	17471841	TP
HP:0011675	Arrhythmia		FP	HP:0002758	Osteoarthritis		FP
HP:0000939	Osteoporosis		FP	HP:0001878	Hemolytic anemia		FP
HP:0006554	Acute hepatic failure		FP	HP:0000833	Glucose intolerance		FP
HP:0002960	Autoimmunity		FP	HP:0002608	Celiac disease		FP
HP:0001409	Portal hypertension	7557861	TP	HP:0004810	Congenital hypoplastic anemia		FP
HP:0001541	Ascites	8867884	TP	HP:0001324	Muscle weakness		FP
HP:0005505	Refractory anemia		FP	HP:0001513	Obesity		FP
HP:0001915	Aplastic anemia		FP	HP:0004444	Spherocytosis		FP
HP:0006580	Portal fibrosis	18160317	TP	HP:0000718	Aggressive behavior		FP
HP:0002863	Myelodysplasia		FP	HP:0000952	Jaundice	19477142	TP
HP:0000821	Hypothyroidism		FP	HP:0100806	Sepsis		FP
HP:0002027	Abdominal pain	6418636	TP	HP:0002621	Atherosclerosis		FP
HP:0003256	Abnormality of the coagulation cascade		FP	HP:0001370	Rheumatoid arthritis		FP
HP:0000953	Hyperpigmentation of the skin	2986052	TP	HP:0100544	Neoplasm of the heart		FP
HP:0001900	Increased hemoglobin		FP	HP:0004870	Chronic hemolytic anemia		FP
HP:0000083	Renal insufficiency		FP	HP:0001824	Weight loss		FP
HP:0002527	Falls		FP	HP:0003231	Hypertyrosinemia		FP
HP:0011031	Abnormality of iron homeostasis		FP	HP:0003881	Humeral sclerosis		FP
HP:0011034	Amyloidosis		FP	HP:0000938	Osteopenia		FP
HP:0002719	Recurrent infections		FP	HP:0004325	Decreased body weight		FP
HP:0000992	Cutaneous photosensitivity		FP	HP:0002480	Hepatic encephalopathy	1936813	TP
HP:0001723	Restrictive cardiomyopathy	6418103	TP	HP:0004377	Hematological neoplasm		FP
HP:0001943	Hypoglycemia		FP	HP:0002619	Varicose veins		FP
HP:0002511	Alzheimer disease		FP	HP:0001413	Micronodular cirrhosis	3909817	TP
HP:0001744	Splenomegaly	24343468	TP	HP:0001658	Myocardial infarction		FP
HP:0001405	Periportal fibrosis	474711	TP	HP:0001945	Fever		FP

continued on the next page

Table S6. Hemochromatosis – continued

ID	Name	pmid	status	ID	Name	pmid	status
HP:0100523	Liver abscess		FP	HP:0000740	Anxiety (with pheochromocytoma)		FP
HP:0000518	Cataract		FP	HP:0001254	Lethargy	16315132	TP
HP:0003073	Hypoalbuminemia	9543801 1312985	TP	HP:0000829	Hypoparathyroidism	7572161	TP
HP:0002611	Cholestatic liver disease	9285385	TP	HP:0000822	Hypertension		FP
HP:0000842	Hyperinsulinemia		FP	HP:0004787	Fulminant hepatitis		FP
HP:0012024	Hypergalactosemia		FP	HP:0000029	Testicular atrophy	21549511	FN
HP:0001404	Hepatocellular necrosis	20665379	FN	HP:0000823	Delayed puberty	8432779	FN
HP:0002749	Osteomalacia	2783312	FN	HP:0001433	Hepatosplenomegaly	24343468	FN
HP:0000141	Amenorrhea	8867884	FN	HP:0000789	Infertility	7263194 14991275	FN
HP:0009830	Peripheral neuropathy	20358215	FN	HP:0000771	Gynecomastia	1392425	FN
HP:0000869	Secondary amenorrhea	8867884	FN	HP:0001387	Joint stiffness	6652983 19018338	FN
HP:0003155	Elevated alkaline phosphatase	1914539	FN	HP:0100769	Synovitis	19933745	FN
HP:0010788	Testicular neoplasm	21549511	FN				

Table S7. Overview of HPO annotations for **Anti-Glomerular Basement Membrane Disease** that were derived by concept recognition in PubMed using BioLark. There were 28 true positives, 17 false positives, and 22 false negatives.

ID	Name	pmid	status	ID	Name	pmid	status
HP:0000099	Glomerulonephritis	7246141	TP	HP:0000093	Proteinuria	9453010	TP
HP:0003453	Antineutrophil antibody positivity	19695059	TP	HP:0002960	Autoimmunity	10896942	TP
HP:0000123	Nephritis	948570	TP	HP:0002633	Vasculitis	9469509	TP
HP:0002105	Hemoptysis	11523135	TP	HP:0000083	Renal insufficiency	7246141	TP
HP:0002955	Granulomatosis		FP	HP:0008653	Crescentic glomerulonephritis	6211894	TP
HP:0000790	Hematuria	8336406	TP	HP:0001919	Acute renal failure	12727586	TP
HP:0003774	End stage renal disease	4011844	TP	HP:0002725	Systemic lupus erythematosus		FP
HP:0000794	IgA nephropathy		FP	HP:0000112	Nephropathy	6211894	TP
HP:0002093	Respiratory insufficiency	22251235	TP	HP:0001903	Anemia	8431025	TP
HP:0002113	Pulmonary infiltrates	4023439	TP	HP:0003259	Increased creatinine	8084449	TP
HP:0000979	Purpura		FP	HP:0000793	Membranoproliferative glomerulonephritis		FP
HP:0000718	Aggressive behavior		FP	HP:0100520	Oliguria	19151145	TP
HP:0003881	Humeral sclerosis		FP	HP:0100519	Anuria	19151145	TP
HP:0000097	Focal segmental glomerulosclerosis		FP	HP:0000100	Nephrotic syndrome	794860	TP
HP:0000096	Glomerulosclerosis	15496153	TP	HP:0001970	Tubulointerstitial nephritis		FP
HP:0002907	Microhematuria	7246141	TP	HP:0100820	Glomerulopathy	8971896	TP
HP:0001945	Fever	8203372	TP	HP:0000822	Hypertension		FP
HP:0006530	Interstitial pulmonary disease	8431025	TP	HP:0011944	Small vessel vasculitis		FP
HP:0002157	Azotemia	16408434	TP	HP:0000969	Edema		FP
HP:0001370	Rheumatoid arthritis		FP	HP:0003493	Antinuclear antibody positivity	16767317	TP
HP:0002206	Pulmonary fibrosis		FP	HP:0003613	Antiphospholipid antibody positivity		FP
HP:0100598	Pulmonary edema		FP	HP:0006535	Recurrent intrapulmonary hemorrhage	3917391	TP
HP:0001973	Autoimmune thrombocytopenia		FP	HP:0002098	Respiratory distress	9361103	FN
HP:0002094	Dyspnea	2214405	FN	HP:0001891	Iron deficiency anemia	8532389	FN
HP:0001250	Seizures	22251235	FN	HP:0005576	Tubulointerstitial fibrosis	17516154	FN
HP:0002875	Exertional dyspnea	10496107	FN	HP:0001880	Eosinophilia	12955709	FN
HP:0001897	Normocytic anemia	16894954	FN	HP:0000622	Blurred vision	8409194	FN
HP:0005521	Disseminated intravascular coagulation	10087878	FN	HP:0003326	Myalgia	8203372	FN
HP:0001342	Cerebral hemorrhage	9355084	FN	HP:0000541	Retinal detachment	8409194	FN
HP:0002039	Anorexia	8820507	FN	HP:0000821	Hypothyroidism	10720217	FN
HP:0007898	Exudative retinopathy	8409194	FN	HP:0200029	Vasculitis in the skin	3184080	FN
HP:0001935	Microcytic anemia	10502944	FN	HP:0003075	Hypoproteinemia	10502944	FN
HP:0000121	Nephrocalcinosis	930188	FN	HP:0003324	Generalized muscle weakness	10750432	FN
HP:0001954	Episodic fever	23515881	FN				

Table S8. Overview of HPO annotations for **Common Variable Immunodeficiency** that were derived by concept recognition in PubMed using BioLark. There were 42 true positives, 27 false positives, and 32 false negatives.

ID	Name	pmid	status	ID	Name	pmid	status
HP:0002721	Immunodeficiency	-	TP	HP:0004313	Hypogammaglobulinemia	19671377	TP
HP:0002960	Autoimmunity		FP	HP:0002719	Recurrent infections	-	TP
HP:0004432	Agammaglobulinemia		FP	HP:0002718	Recurrent bacterial infections	11861266	TP
HP:0002720	IgA deficiency	22983507	TP	HP:0002110	Bronchiectasis	20635788	TP
HP:0004315	IgG deficiency	20434118	TP	HP:0001744	Splenomegaly	9822285	TP
HP:0002205	Recurrent respiratory infections	16794375	TP	HP:0011947	Respiratory tract infection		FP
HP:0002843	Abnormality of T cells	10993290	TP	HP:0002665	Lymphoma	20332369	TP
HP:0002958	Immune dysregulation	19671377	TP	HP:0005425	Recurrent sinopulmonary infections	20402074	TP
HP:0002090	Pneumonia	-	TP	HP:0002850	IgM deficiency	23379434	TP
HP:0001973	Autoimmune thrombocytopenia	19716342	TP	HP:0002028	Chronic diarrhea	17629033	TP
HP:0002024	Malabsorption	15248108	TP	HP:0001890	Autoimmune hemolytic anemia	19671377	TP
HP:0006532	Recurrent pneumonia	12709641	TP	HP:0005479	IgE deficiency		FP
HP:0005523	Lymphoproliferative disorder	17601274	TP	HP:0001888	Lymphopenia	12165093	TP
HP:0002242	Abnormality of the intestine		FP	HP:0005435	Impaired T cell function	8050170	TP
HP:0001903	Anemia	16789508	TP	HP:0002014	Diarrhea		FP
HP:0004430	Severe combined immunodeficiency		FP	HP:0006530	Interstitial pulmonary disease	22930256	TP
HP:0002037	Inflammation of the large intestine		FP	HP:0002608	Celiac disease		FP
HP:0000246	Sinusitis	18419489	TP	HP:0001873	Thrombocytopenia		FP
HP:0010702	Hypergammaglobulinemia		FP	HP:0011473	Villous atrophy	14550517	TP
HP:0003095	Septic arthritis	8945717	TP	HP:0005365	Severe B lymphocytopenia	8027379	TP
HP:0002846	Abnormality of B cells	17521034	TP	HP:0002099	Asthma		FP
HP:0004798	Recurrent infection of the gastrointestinal tract	18953945	TP	HP:0001875	Neutropenia	17165275	TP
HP:0006528	Chronic lung disease	22180439	TP	HP:0001945	Fever	17165275	TP
HP:0001370	Rheumatoid arthritis	19671377	TP	HP:0005357	Defective B cell differentiation	23714403	TP
HP:0003237	Increased IgG level		FP	HP:0002783	Recurrent lower respiratory tract infections	12164371	TP
HP:0000979	Purpura	19671377	TP	HP:0011108	Recurrent sinusitis	15005811	TP
HP:0001009	Telangiectasia		FP	HP:0000388	Otitis media	19230900	TP
HP:0001399	Hepatic failure		FP	HP:0006515	Interstitial pneumonitis		FP
HP:0001251	Ataxia		FP	HP:0100280	Crohn's disease		FP
HP:0006527	Lymphoid interstitial pneumonia	12709641	TP	HP:0002583	Colitis		FP
HP:0003139	Panhypogammaglobulinemia		FP	HP:0100827	Lymphocytosis	17194667	TP
HP:0005432	Transient hypogammaglobulinemia of infancy		FP	HP:0002961	Dysgammaglobulinemia		FP
HP:0003496	Increased IgM level		FP	HP:0011950	Bronchiolitis		FP
HP:0010701	Abnormal immunoglobulin level		FP	HP:0010977	Abnormality of phagocytes		FP
HP:0002209	Sparse scalp hair		FP	HP:0002729	Follicular hyperplasia	18054123	FN
HP:0011109	Chronic sinusitis	16252205	FN	HP:0001433	Hepatosplenomegaly	17601274	FN
HP:0200043	Verrucae	17902733	FN	HP:0005681	Juvenile rheumatoid arthritis	17671947	FN
HP:0002955	Granulomatosis	16413828	FN	HP:0005387	Combined immunodeficiency	11514920	FN
HP:0001876	Pancytopenia	22413915	FN	HP:0001878	Hemolytic anemia	19716342	FN

continued on the next page

Table S8. Common Variable Immunodeficiency – continued

ID	Name	pmid	status	ID	Name	pmid	status
HP:0000554	Uveitis	22506485	FN	HP:0002788	Recurrent upper respiratory tract infections	18419489	FN
HP:0001409	Portal hypertension	23420139	FN	HP:0001581	Recurrent skin infections	19419461	FN
HP:0005263	Gastritis	8228799	FN	HP:0002716	Lymphadenopathy	17556024	FN
HP:0100279	Ulcerative colitis	16329682	FN	HP:0002633	Vasculitis	10682991	FN
HP:0002725	Systemic lupus erythematosus	19671377	FN	HP:0001287	Meningitis	15513403	FN
HP:0001369	Arthritis	19326121 16909702 15875533 21776287 17671947	FN	HP:0005419	Decreased T cell activation	19671377	FN
HP:0005390	Recurrent opportunistic infections	-	FN	HP:0001904	Autoimmune neutropenia	16127007	FN
HP:0100721	Mediastinal lymphadenopathy	20635788	FN	HP:0001045	Vitiligo	21139556	FN
HP:0000010	Recurrent urinary tract infections	-	FN	HP:0100646	Thyroiditis	19671377	FN
HP:0010976	B lymphocytopenia	8027379	FN	HP:0100537	Fasciitis	11809601 23129076 22575775	FN
HP:0006946	Recurrent meningitis	15591667	FN	HP:0003613	Antiphospholipid antibody positivity	20635793 21776287	FN
HP:0011117	Abnormality of interleukin secretion	7586680	FN				

Table S9. Overview of HPO annotations for **Biliary Liver Cirrhosis** that were derived by concept recognition in PubMed using BioLark. There were 32 true positives, 40 false positives, and 34 false negatives.

ID	Name	pmid	status	ID	Name	pmid	status
HP:0002613	Biliary cirrhosis	6896227	TP	HP:0001394	Cirrhosis	-	TP
HP:0001396	Cholestasis	7082202	TP	HP:0002611	Cholestatic liver disease	15560038	TP
HP:0000989	Pruritus	22259000	TP	HP:0000952	Jaundice	4743495	TP
HP:0001409	Portal hypertension	7091126	TP	HP:0001399	Hepatic failure	15560038	TP
HP:0003493	Antinuclear antibody positivity	3894432	TP	HP:0006562	Viral hepatitis		FP
HP:0001541	Ascites	20606498	TP	HP:0003155	Elevated alkaline phosphatase	17918011	TP
HP:0002619	Varicose veins		FP	HP:0001402	Hepatocellular carcinoma	15560042	TP
HP:0001395	Hepatic fibrosis	22042492	TP	HP:0001406	Intrahepatic cholestasis		FP
HP:0005912	Biliary atresia		FP	HP:0002725	Systemic lupus erythematosus		FP
HP:0001397	Hepatic steatosis		FP	HP:0002040	Esophageal varices	1085267	TP
HP:0000939	Osteoporosis	14594136	TP	HP:0001370	Rheumatoid arthritis		FP
HP:0002910	Elevated hepatic transaminases	15560032	TP	HP:0100324	Scleroderma	17294883	TP
HP:0003881	Humeral sclerosis		FP	HP:0001410	Decreased liver function	-	TP
HP:0006580	Portal fibrosis		FP	HP:0001081	Cholelithiasis		FP
HP:0100279	Ulcerative colitis		FP	HP:0000938	Osteopenia	7659915	TP
HP:0001408	Bile duct proliferation	8778189	TP	HP:0002480	Hepatic encephalopathy	21641685	TP
HP:0002240	Hepatomegaly	21989789	TP	HP:0010702	Hypergammaglobulinemia		FP
HP:0002749	Osteomalacia		FP	HP:0001324	Muscle weakness		FP
HP:0002608	Celiac disease		FP	HP:0001369	Arthritis		FP
HP:0100646	Thyroiditis		FP	HP:0002527	Falls		FP
HP:0000820	Abnormality of the thyroid gland		FP	HP:0000872	Hashimoto thyroiditis		FP
HP:0002239	Gastrointestinal hemorrhage		FP	HP:0011838	Sclerodactyly		FP
HP:0000718	Aggressive behavior		FP	HP:0000969	Edema		FP
HP:0003124	Hypercholesterolemia	4030709	TP	HP:0003573	Increased total bilirubin		FP
HP:0000010	Recurrent urinary tract infections		FP	HP:0100512	Vitamin D deficiency	73950	TP
HP:0003453	Antineutrophil antibody positivity		FP	HP:0001000	Abnormality of skin pigmentation		FP
HP:0000991	Xanthomatosis	4346939	TP	HP:0001945	Fever		FP
HP:0000083	Renal insufficiency		FP	HP:0003365	Arthralgia of the hip		FP
HP:0001009	Telangiectasia	12356109	TP	HP:0001947	Renal tubular acidosis	5548562	TP
HP:0100513	Vitamin E deficiency	2910763	TP	HP:0003765	Psoriasis		FP
HP:0003077	Hyperlipidemia		FP	HP:0001045	Vitiligo	16481294	TP
HP:0004448	Fulminant hepatic failure		FP	HP:0000093	Proteinuria		FP
HP:0011892	Vitamin K deficiency	11569705	TP	HP:0000819	Diabetes mellitus		FP
HP:0000855	Insulin resistance		FP	HP:0003259	Increased creatinine		FP
HP:0001954	Episodic fever		FP	HP:0003073	Hypoalbuminemia	16181370	TP
HP:0000988	Skin rash	17060877	TP	HP:0003149	Hyperuricosuria		FP
HP:0011954	Nodular regenerative hyperplasia of liver	2583572	FN	HP:0001114	Xanthelasma	1420396	FN
HP:0003496	Increased IgM level	14987744	FN	HP:0008341	Distal renal tubular acidosis	15610460	FN
HP:0001404	Hepatocellular necrosis	1882800	FN	HP:0002570	Steatorrhea	2411648	FN
HP:0008151	Prolonged prothrombin time	20856137	FN	HP:0004315	IgG deficiency	21645440	FN
HP:0011473	Villous atrophy	9412913	FN	HP:0001097	Keratoconjunctivitis sicca	15539725	FN

continued on the next page

Table S9. Biliary Liver Cirrhosis – continued

ID	Name	pmid	status	ID	Name	pmid	status
HP:0003761	Calcinosis	12356109	FN	HP:0002958	Immune dysregulation	22135136	FN
HP:0001970	Tubulointerstitial nephritis	17294883 20466658	FN	HP:0001262	Somnolence	18237872	FN
HP:0002653	Bone pain	8878772	FN	HP:0001254	Lethargy	15456326	FN
HP:0001433	Hepatosplenomegaly	6324495	FN	HP:0002756	Pathologic fracture	20926953	FN
HP:0002459	Dysautonomia	19602135	FN	HP:0100614	Myositis	15287510 23553600	FN
HP:0001973	Autoimmune thrombocytopenia	4054707 8680553	FN	HP:0002904	Hyperbilirubinemia	11206871	FN
HP:0002757	Recurrent fractures	17087953	FN	HP:0006554	Acute hepatic failure	17657817	FN
HP:0002024	Malabsorption	7429337	FN	HP:0001880	Eosinophilia	8633501	FN
HP:0002039	Anorexia	4030709	FN	HP:0002027	Abdominal pain	7942679	FN
HP:0001824	Weight loss	9820402	FN	HP:0003262	Smooth muscle antibody positivity	7549131	FN
HP:0002630	Fat malabsorption	3335317	FN	HP:0006577	Macronodular cirrhosis	6217390	FN
HP:0200032	Kayser-Fleischer ring	842986 8458236 1150026	FN	HP:0100759	Clubbing of fingers	7227854	FN

Table S10. Overview of HPO annotations for **Dermatomyositis** that were derived by concept recognition in PubMed using BioLark. There were 39 true positives, 80 false positives, and 19 false negatives.

ID	Name	pmid	status	ID	Name	pmid	status
HP:0100614	Myositis	4753878	TP	HP:0009071	Inflammatory myopathy	9438396	TP
HP:0006530	Interstitial pulmonary disease	23117947	TP	HP:0002725	Systemic lupus erythematosus		FP
HP:0001324	Muscle weakness		FP	HP:0003761	Calcinosis	7017918	TP
HP:0100324	Scleroderma		FP	HP:0003701	Proximal muscle weakness	9438396	TP
HP:0003198	Myopathy		FP	HP:0003881	Humeral sclerosis		FP
HP:0010783	Erythema	23117947	TP	HP:0002633	Vasculitis	1845413	TP
HP:0003493	Antinuclear antibody positivity	6787993	TP	HP:0001370	Rheumatoid arthritis		FP
HP:0000988	Skin rash	1767082	TP	HP:0001369	Arthritis	23117947	TP
HP:0003326	Myalgia	11003943	TP	HP:0002015	Dysphagia		FP
HP:0002206	Pulmonary fibrosis		FP	HP:0005681	Juvenile rheumatoid arthritis		FP
HP:0001945	Fever		FP	HP:0000718	Aggressive behavior		FP
HP:0006515	Interstitial pneumonitis	16328018	TP	HP:0002093	Respiratory insufficiency		FP
HP:0003560	Muscular dystrophy		FP	HP:0003236	Elevated serum creatine phosphokinase		FP
HP:0000969	Edema		FP	HP:0000964	Eczema		FP
HP:0003202	Amyotrophy		FP	HP:0200042	Skin ulcer	17572631	TP
HP:0003473	Fatigable weakness		FP	HP:0001009	Telangiectasia	1845413	TP
HP:0003323	Progressive muscle weakness	11359403	TP	HP:0001371	Flexion contracture	23117947	TP
HP:0008978	Necrotizing myopathy		FP	HP:0002090	Pneumonia		FP
HP:0002665	Lymphoma		FP	HP:0100539	Periorbital edema	9557787	TP
HP:0011945	Bronchiolitis obliterans organizing pneumonia	1246203	TP	HP:0002094	Dyspnea		FP
HP:0003365	Arthralgia of the hip		FP	HP:0002861	Malignant melanoma		FP
HP:0000989	Pruritus	23112358	TP	HP:0100615	Ovarian neoplasm		FP
HP:0003765	Psoriasis		FP	HP:0100633	Esophagitis		FP
HP:0000956	Acanthosis nigricans		FP	HP:0003805	Rimmed vacuoles		FP
HP:0005059	arthralgia/arthritis	3977973	TP	HP:0000992	Cutaneous photosensitivity	15379871	TP
HP:0007430	Generalized edema	18984850	TP	HP:0001029	Poikiloderma	23112358	TP
HP:0002097	Emphysema		FP	HP:0002829	Arthralgia	23117947	TP
HP:0001888	Lymphopenia		FP	HP:0000979	Purpura		FP
HP:0003002	Breast carcinoma		FP	HP:0011951	Aspiration pneumonia		FP
HP:0001041	Facial erythema	17215624	TP	HP:0001973	Autoimmune thrombocytopenia		FP
HP:0100537	Fasciitis	15197005	TP	HP:0003457	EMG abnormality	15693592	TP
HP:0011123	Inflammatory abnormality of the skin		FP	HP:0002955	Granulomatosis		FP
HP:0007417	Discoid lupus erythematosus		FP	HP:0007354	Amyotrophic lateral sclerosis		FP
HP:0002721	Immunodeficiency		FP	HP:0009073	Progressive proximal muscle weakness	16132164	TP
HP:0009125	Lipodystrophy	22044089	TP	HP:0001482	Subcutaneous nodules		FP
HP:0003324	Generalized muscle weakness	16866067	TP	HP:0003713	Muscle fiber necrosis	1423335	TP
HP:0000951	Abnormality of the skin		FP	HP:0200029	Vasculitis in the skin	18981641	TP
HP:0002092	Pulmonary hypertension		FP	HP:0007618	Subcutaneous calcification	8687325	TP
HP:0100646	Thyroiditis		FP	HP:0001596	Alopecia	23112358	TP
HP:0002875	Exertional dyspnea		FP	HP:0002835	Aspiration		FP
HP:0001289	Confusion		FP	HP:0003750	Increased muscle fatiguability	17907213	TP
HP:0003259	Increased creatinine		FP	HP:0002027	Abdominal pain		FP
HP:0000998	Hypertrichosis		FP	HP:0011675	Arrhythmia		FP
HP:0002613	Biliary cirrhosis		FP	HP:0001271	Polyneuropathy		FP

continued on the next page

Table S10. Dermatomyositis – continued

ID	Name	pmid	status	ID	Name	pmid	status
HP:0003565	Elevated erythrocyte sedimentation rate	2334184	TP	HP:0002107	Pneumothorax		FP
HP:0000083	Renal insufficiency		FP	HP:0002747	Respiratory insufficiency due to muscle weakness	16386077	TP
HP:0000093	Proteinuria		FP	HP:0001824	Weight loss		FP
HP:0001618	Dysphonia	23042610 16467366	TP	HP:0001047	Atopic dermatitis		FP
HP:0000939	Osteoporosis		FP	HP:0001903	Anemia		FP
HP:0001880	Eosinophilia		FP	HP:0005523	Lymphoproliferative disorder		FP
HP:0003756	Skeletal myopathy	15196172	TP	HP:0001878	Hemolytic anemia		FP
HP:0010702	Hypergammaglobulinemia		FP	HP:0007269	Spinal muscular atrophy		FP
HP:0006532	Recurrent pneumonia		FP	HP:0002098	Respiratory distress		FP
HP:0003700	Generalized amyotrophy		FP	HP:0002863	Myelodysplasia		FP
HP:0006775	Multiple myeloma		FP	HP:0004432	Agammaglobulinemia		FP
HP:0004313	Hypogammaglobulinemia		FP	HP:0003458	EMG: myopathic abnormalities		FP
HP:0003715	Myofibrillar myopathy		FP	HP:0001597	Abnormality of the nail		FP
HP:0008942	Acute rhabdomyolysis		FP	HP:0000158	Macroglossia		FP
HP:0002249	Melena		FP	HP:0005781	Contractures of the large joints		FP
HP:0007126	Proximal amyotrophy		FP	HP:0100540	Palpebral edema	12325332	FN
HP:0100295	Muscle fiber atrophy	23112358	FN	HP:0002460	Distal muscle weakness	18203322	FN
HP:0002792	Reduced vital capacity	15692974	FN	HP:0100578	Lipoatrophy	8436656	FN
HP:0003546	Exercise intolerance	21106107	FN	HP:0002923	Rheumatoid factor positive	6965409	FN
HP:0001685	Myocardial fibrosis	4081664	FN	HP:0002102	Pleuritis	3813671	FN
HP:0000962	Hyperkeratosis	17215624	FN	HP:0003453	Antineutrophil antibody positivity	21812362	FN
HP:0008064	Ichthyosiform abnormality of the skin	22515579	FN	HP:0001701	Pericarditis	8444002	FN
HP:0010766	Ectopic calcification	18448482	FN	HP:0002960	Autoimmunity	23117947	FN
HP:0100249	Calcification of muscles	8814715	FN	HP:0200044	Porokeratosis	17173828	FN
HP:0000965	Cutis marmorata	9731966 1845403	FN	HP:0001019	Erythroderma	8814715	FN

Table S11. Overview of HPO annotations for **Osteoporosis** that were derived by concept recognition in PubMed using BioLark. There were 18 true positives, 109 false positives, and 14 false negatives.

ID	Name	pmid	status	ID	Name	pmid	status
HP:0000939	Osteoporosis	6918601	TP	HP:0000938	Osteopenia	10531790	TP
HP:0002757	Recurrent fractures	10100933 15560040 21394493 12810179 12815335	TP	HP:0005897	Severe osteoporosis	-	TP
HP:0002953	Vertebral compression fractures	7083689	TP	HP:0011001	Increased bone mineral density		FP
HP:0002527	Falls		FP	HP:0002797	Osteolysis		FP
HP:0002659	Increased susceptibility to fractures	11866149	TP	HP:0001370	Rheumatoid arthritis		FP
HP:0100512	Vitamin D deficiency		FP	HP:0002749	Osteomalacia	-	TP
HP:0000135	Hypogonadism		FP	HP:0010885	Aseptic necrosis		FP
HP:0003418	Back pain	10197021	TP	HP:0002756	Pathologic fracture		FP
HP:0002758	Osteoarthritis		FP	HP:0000867	Secondary hyperparathyroidism		FP
HP:0003002	Breast carcinoma		FP	HP:0003072	Hypercalcemia		FP
HP:0001324	Muscle weakness		FP	HP:0002808	Kyphosis	19640824	TP
HP:0100787	Prostate neoplasm		FP	HP:0001513	Obesity		FP
HP:0002150	Hypercalciuria		FP	HP:0004325	Decreased body weight		FP
HP:0008200	Primary hyperparathyroidism		FP	HP:0002037	Inflammation of the large intestine		FP
HP:0000819	Diabetes mellitus		FP	HP:0003978	Fractured radius	7083689	TP
HP:0000141	Amenorrhea		FP	HP:0000843	Hyperparathyroidism		FP
HP:0002653	Bone pain		FP	HP:0002024	Malabsorption		FP
HP:0002039	Anorexia		FP	HP:0004395	Malnutrition		FP
HP:0001824	Weight loss		FP	HP:0000822	Hypertension		FP
HP:0100646	Thyroiditis		FP	HP:0100280	Crohn's disease		FP
HP:0006775	Multiple myeloma		FP	HP:0000836	Hyperthyroidism		FP
HP:0007354	Amyotrophic lateral sclerosis		FP	HP:0000704	Periodontitis		FP
HP:0001297	Stroke		FP	HP:0003419	Low back pain	22338309	TP
HP:0001578	Hypercortisolism		FP	HP:0006510	Chronic obstructive pulmonary disease		FP
HP:0002901	Hypocalcemia		FP	HP:0002621	Atherosclerosis		FP
HP:0008443	Spinal deformities		FP	HP:0002608	Celiac disease		FP
HP:0005625	Osteoporosis of vertebrae	-	TP	HP:0002099	Asthma		FP
HP:0000969	Edema		FP	HP:0003155	Elevated alkaline phosphatase	7083689	TP
HP:0002960	Autoimmunity		FP	HP:0002063	Rigidity		FP
HP:0000083	Renal insufficiency		FP	HP:0002725	Systemic lupus erythematosus		FP
HP:0000787	Nephrolithiasis		FP	HP:0003774	End stage renal disease		FP
HP:0003869	Cortical thinning (humeral)	1281535	TP	HP:0100279	Ulcerative colitis		FP
HP:0001903	Anemia		FP	HP:0001510	Growth delay		FP
HP:0008422	Vertebral wedging	18395504	TP	HP:0004324	Increased body weight		FP
HP:0002613	Biliary cirrhosis		FP	HP:0004934	Vascular calcification		FP
HP:0003077	Hyperlipidemia		FP	HP:0004349	Reduced bone mineral density	16265206	TP
HP:0001289	Confusion		FP	HP:0001394	Cirrhosis		FP
HP:0004789	Lactose intolerance		FP	HP:0000726	Dementia		FP
HP:0100495	Mastocytosis		FP	HP:0001250	Seizures		FP
HP:0000870	Prolactin excess		FP	HP:0000855	Insulin resistance		FP
HP:0000024	Prostatitis		FP	HP:0002511	Alzheimer disease		FP
HP:0000718	Aggressive behavior		FP	HP:0001907	Thromboembolism		FP
HP:0000829	Hypoparathyroidism		FP	HP:0004322	Short stature		FP
HP:0000708	Behavioural/Psychiatric Abnormality		FP	HP:0003202	Amyotrophy		FP
HP:0000821	Hypothyroidism		FP	HP:0001635	Congestive heart failure		FP
HP:0000740	Anxiety (with pheochromocytoma)		FP	HP:0003470	Paralysis		FP
HP:0001300	Parkinsonism		FP	HP:0003259	Increased creatinine		FP
HP:0002611	Cholestatic liver disease		FP	HP:0100544	Neoplasm of the heart		FP
HP:0000737	Irritability		FP	HP:0000823	Delayed puberty		FP
HP:0100543	Cognitive impairment		FP	HP:0000026	Male hypogonadism		FP
HP:0100753	Schizophrenia		FP	HP:0000824	Growth hormone deficiency		FP

continued on the next page

Table S11. Osteoporosis – continued

ID	Name	pmid	status	ID	Name	pmid	status
HP:0002204	Pulmonary embolism		FP	HP:0001249	Intellectual disability		FP
HP:0010550	Paraplegia		FP	HP:0003003	Colon cancer		FP
HP:0000820	Abnormality of the thyroid gland		FP	HP:0000789	Infertility		FP
HP:0001658	Myocardial infarction		FP	HP:0003765	Psoriasis		FP
HP:0000786	Primary amenorrhea		FP	HP:0000845	Growth hormone excess		FP
HP:0100021	Cerebral palsy		FP	HP:0009830	Peripheral neuropathy		FP
HP:0006528	Chronic lung disease		FP	HP:0011986	Ectopic ossification		FP
HP:0002665	Lymphoma		FP	HP:0001909	Leukemia		FP
HP:0002097	Emphysema		FP	HP:0006536	Obstructive lung disease		FP
HP:0002206	Pulmonary fibrosis		FP	HP:0002092	Pulmonary hypertension		FP
HP:0006530	Interstitial pulmonary disease		FP	HP:0004936	Venous thrombosis		FP
HP:0100036	Pseudo-fractures	4036121	TP	HP:0002863	Myelodysplasia		FP
HP:0002752	Sparse bone trabeculae	18299223	TP	HP:0004586	Biconcave vertebral bodies	3659378	FN
HP:0003876	Osteoporotic humerus	7083689	FN	HP:0003080	Hydroxyprolinuria	1887826	FN
HP:0008428	Vertebral clefting	16091506	FN	HP:0004568	Beaking of vertebral bodies	25069705	FN
HP:0003282	Low alkaline phosphatase	9116389 8695849	FN	HP:0004591	Disc-like vertebral bodies	9548357	FN
HP:0003987	Fractured ulna	7083689	FN	HP:0002355	Difficulty walking	9458225	FN
HP:0003084	Fractures of the long bones	7083689	FN	HP:0003302	Spondylolisthesis	11458155	FN
HP:0006640	Multiple rib fractures	16582522	FN	HP:0004699	Osteoporotic metatarsal	20681355	FN
HP:0003964	Osteoporotic forearm bones	1527750	FN				

Table S12. Overview of HPO annotations for **Rickets** that were derived by concept recognition in PubMed using BioLark. There were 20 true positives, 46 false positives, and 13 false negatives.

ID	Name	pmid	status	ID	Name	pmid	status
HP:0002748	Rickets	23374621	TP	HP:0100512	Vitamin D deficiency		FP
HP:0002749	Osteomalacia	23374621	TP	HP:0004912	Hypophosphatemic rickets		FP
HP:0002901	Hypocalcemia	23374621	TP	HP:0002148	Hypophosphatemia	23374621	TP
HP:0003155	Elevated alkaline phosphatase	23374621	TP	HP:0000938	Osteopenia		FP
HP:0001510	Growth delay	23374621	TP	HP:0004395	Malnutrition		FP
HP:0000939	Osteoporosis		FP	HP:0002150	Hypercalciuria	23374621	TP
HP:0001250	Seizures		FP	HP:0000121	Nephrocalcinosis		FP
HP:0003072	Hypercalcemia		FP	HP:0001518	Small for gestational age		FP
HP:0002979	Bowing of the legs	23374621	TP	HP:0002970	Genu varum	23374621	TP
HP:0002024	Malabsorption		FP	HP:0001596	Alopecia		FP
HP:0004322	Short stature	20926527	TP	HP:0000843	Hyperparathyroidism		FP
HP:0002857	Genu valgum	23374621	TP	HP:0002199	Hypocalcemic seizures	12812706	TP
HP:0000829	Hypoparathyroidism		FP	HP:0002905	Hyperphosphatemia		FP
HP:0001324	Muscle weakness		FP	HP:0000117	Decreased renal tubular phosphate reabsorption	1755097	TP
HP:0011002	Osteopetrosis		FP	HP:0002653	Bone pain	23374621	TP
HP:0001281	Tetany	23374621	TP	HP:0000852	Pseudohypoparathyroidism		FP
HP:0001508	Failure to thrive		FP	HP:0003109	Hyperphosphaturia		FP
HP:0002756	Pathologic fracture		FP	HP:0000897	Rachitic rosary	23151726	TP
HP:0001903	Anemia		FP	HP:0001000	Abnormality of skin pigmentation		FP
HP:0003021	Metaphyseal cupping	23151726	TP	HP:0001947	Renal tubular acidosis		FP
HP:0003472	Hypocalcemic tetany	12812706	TP	HP:0100593	Calcification of cartilage		FP
HP:0003020	Enlargement of the wrists	21767417	TP	HP:0002757	Recurrent fractures	23374621	TP
HP:0002814	Abnormality of the lower limb		FP	HP:0011001	Increased bone mineral density		FP
HP:0001622	Premature birth		FP	HP:0005912	Biliary atresia		FP
HP:0100511	Abnormality of vitamin D metabolism		FP	HP:0000787	Nephrolithiasis		FP
HP:0003355	Aminoaciduria		FP	HP:0003076	Glycosuria		FP
HP:0003126	Low-molecular-weight proteinuria		FP	HP:0003282	Low alkaline phosphatase		FP
HP:0006463	Rickets of the lower limbs		FP	HP:0001942	Metabolic acidosis		FP
HP:0001840	Metatarsus adductus		FP	HP:0001225	Wrist swelling		FP
HP:0001949	Hypokalemic alkalosis		FP	HP:0003987	Fractured ulna		FP
HP:0003236	Elevated serum creatine phosphokinase		FP	HP:0006409	Progressive leg bowing		FP
HP:0000945	Flared irregular metaphyses		FP	HP:0003029	Enlargement of the ankles		FP
HP:0003215	Dicarboxylic aciduria		FP	HP:0002986	Radial bowing		FP
HP:0002007	Frontal bossing	19576150	FN	HP:0008208	Parathyroid hyperplasia	23374621	FN
HP:0003084	Fractures of the long bones	23374621	FN	HP:0009763	Limb pain	23374621	FN
HP:0002829	Arthralgia	20418553	FN	HP:0001288	Gait disturbance	8699350	FN
HP:0006487	Bowing of the long bones	7419691	FN	HP:0000920	Enlargement of the costochondral junction	23220549	FN
HP:0008732	Renal hypophosphatemia	2983252	FN	HP:0003016	Metaphyseal widening	21795457	FN
HP:0002355	Difficulty walking	19576150	FN	HP:0005897	Severe osteoporosis	8250499	FN
HP:0002659	Increased susceptibility to fractures	23374621	FN				

Table S13. Overview of HPO annotations for **Lepromatous Leprosy** that were derived by concept recognition in PubMed using BioLark. There were 46 true positives, 33 false positives, and 46 false negatives.

ID	Name	pmid	status	ID	Name	pmid	status
HP:0010783	Erythema	18627717	TP	HP:0009830	Peripheral neuropathy	1802936	TP
HP:0002633	Vasculitis	10347568	TP	HP:0001945	Fever	9522583	TP
HP:0200036	Skin nodule	18313706	TP	HP:0200042	Skin ulcer	16638386	TP
HP:0000656	Ectropion	12831146	TP	HP:0002835	Aspiration		FP
HP:0002527	Falls		FP	HP:0001324	Muscle weakness		FP
HP:0000969	Edema	24770495	TP	HP:0001482	Subcutaneous nodules	2119410	TP
HP:0002721	Immunodeficiency		FP	HP:0001128	Trichiasis	12831146	TP
HP:0100699	Scarring	24770495	TP	HP:0000246	Sinusitis	5169808	TP
HP:0000518	Cataract	2657299 12446359	TP	HP:0002960	Autoimmunity		FP
HP:0002716	Lymphadenopathy	15881039	TP	HP:0008066	Abnormal blistering of the skin	9747246	TP
HP:0003474	Sensory impairment	10700912	TP	HP:0002719	Recurrent infections		FP
HP:0100608	Metrorrhagia		FP	HP:0006775	Multiple myeloma		FP
HP:0003470	Paralysis		FP	HP:0001094	Iridocyclitis	1995040	TP
HP:0007354	Amyotrophic lateral sclerosis		FP	HP:0011096	Peripheral demyelination	2852213	TP
HP:0003613	Antiphospholipid antibody positivity	1669564	TP	HP:0011107	Recurrent aphthous stomatitis		FP
HP:0000975	Hyperhidrosis		FP	HP:0000988	Skin rash	17551381	TP
HP:0001903	Anemia	1402625	TP	HP:0007759	Opacification of the corneal stroma	2657299	TP
HP:0002840	Lymphadenitis	15881039	TP	HP:0001045	Vitiligo	11123444	TP
HP:0001019	Erythroderma		FP	HP:0001000	Abnormality of skin pigmentation	16044817	TP
HP:0001089	Iris atrophy	12831146	TP	HP:0000554	Uveitis	9524032	TP
HP:0000999	Pyoderma		FP	HP:0011859	Punctate keratitis	12831146	TP
HP:0001075	Atrophic scars	15282970	TP	HP:0000964	Eczema		FP
HP:0000491	Keratitis	9524032	TP	HP:0002019	Constipation		FP
HP:0000718	Aggressive behavior		FP	HP:0001271	Polyneuropathy	22270208 10432812	TP
HP:0001101	Iritis	8862265	TP	HP:0001369	Arthritis	17976874	TP
HP:0100532	Scleritis	11967738	TP	HP:0002665	Lymphoma		FP
HP:0002725	Systemic lupus erythematosus		FP	HP:0001067	Neurofibromas		FP
HP:0003447	Axonal loss	14506718	TP	HP:0100726	Kaposi's sarcoma		FP
HP:0002860	Squamous cell carcinoma	1787225 8089361 3198958 3198959	TP	HP:0000951	Abnormality of the skin		FP
HP:0000099	Glomerulonephritis	2496359	TP	HP:0011873	Abnormal platelet count	22607288 9782435	TP
HP:0002459	Dysautonomia	2358707	TP	HP:0001171	Ectrodactyly (hands)		FP
HP:0011120	Saddle nose	22170033	TP	HP:0004326	Cachexia		FP
HP:0002102	Pleuritis	18567421 9147904	TP	HP:0003401	Paresthesia	19603298	TP
HP:0003365	Arthralgia of the hip		FP	HP:0000621	Entropion		FP
HP:0002829	Arthralgia	16638426	TP	HP:0001581	Recurrent skin infections		FP
HP:0000798	Oligospermia		FP	HP:0000027	Azoospermia	7921941	TP

continued on the next page

Table S13. Lepromatous Leprosy – continued

ID	Name	pmid	status	ID	Name	pmid	status
HP:0001917	Renal amyloidosis	2496359	TP	HP:0001289	Confusion		FP
HP:0001262	Somnolence		FP	HP:0000421	Epistaxis	9133791 21938685	TP
HP:0009829	Phocomelia		FP	HP:0001059	Pterygia		FP
HP:0001055	Erysipelas		FP	HP:0000509	Conjunctivitis	3508760	FN
HP:0002625	Deep venous thrombosis	22607288	FN	HP:0001824	Weight loss	2707215	FN
HP:0100646	Thyroiditis	17120512	FN	HP:0000135	Hypogonadism	23235783	FN
HP:0010628	Facial palsy	9251588	FN	HP:0001882	Leukopenia	1947478	FN
HP:0001596	Alopecia	15677976 15022902 23174494	FN	HP:0011034	Amyloidosis	3223746	FN
HP:0000789	Infertility	12914132 7921941	FN	HP:0000221	Furrowed tongue	1431321 8425797	FN
HP:0009831	Mononeuropathy	14750581 2624076	FN	HP:0100495	Mastocytosis	3880309 15022902	FN
HP:0000771	Gynecomastia	2262715 7921941	FN	HP:0000495	Recurrent corneal erosions	2657299 10396193	FN
HP:0000389	Chronic otitis media	2086677	FN	HP:0003651	Foam cells	15056385	FN
HP:0001010	Hypopigmentation of the skin	8532702	FN	HP:0003453	Antineutrophil antibody positivity	10347568	FN
HP:0003493	Antinuclear antibody positivity	10347568	FN	HP:0001025	Urticaria	15835607 17511942	FN
HP:0001876	Pancytopenia	24171241	FN	HP:0000979	Purpura	3275072	FN
HP:0000802	Impotence	2358707 18075988	FN	HP:0003202	Amyotrophy	15581032	FN
HP:0001919	Acute renal failure	3268517	FN	HP:0001291	Abnormality of the cranial nerves	9251586	FN
HP:0000501	Glaucoma	22607288 9782435	FN	HP:0001873	Thrombocytopenia	24171241	FN
HP:0000365	Hearing impairment	7714350	FN	HP:0011123	Inflammatory abnormality of the skin	23133681	FN
HP:0000199	Tongue nodules	8425797	FN	HP:0011469	Nasal regurgitation	8942155	FN
HP:0001982	Sea-blue histiocytosis	16961654	FN	HP:0001063	Acrocyanosis	8745686	FN
HP:0200034	Skin papules	16650172	FN	HP:0200035	skin plaques	16008652	FN
HP:0000649	Abnormality of vision evoked potentials	9251586	FN	HP:0002293	Alopecia of scalp	9503871	FN
HP:0007178	Motor polyneuropathy	18075988	FN	HP:0000029	Testicular atrophy	7921941	FN
HP:0100686	Enthesitis	8782134	FN	HP:0006480	Premature loss of teeth	17072249	FN
HP:0100778	Cryoglobulinemia	11309832	FN	HP:0000093	Proteinuria	2496359	FN
HP:0011355	Localized skin lesion	1807258	FN				

Table S14. Overview of HPO annotations for **Dirofilariasis** that were derived by concept recognition in PubMed using BioLark. There were 11 true positives, 47 false positives, and 17 false negatives.

ID	Name	pmid	status	ID	Name	pmid	status
HP:0001482	Subcutaneous nodules	23776842	TP	HP:0001807	Ridged nail		FP
HP:0001880	Eosinophilia	9754010	TP	HP:0002092	Pulmonary hypertension		FP
HP:0002580	Volvulus		FP	HP:0006532	Recurrent pneumonia		FP
HP:0002204	Pulmonary embolism	9531965	TP	HP:0001324	Muscle weakness		FP
HP:0001907	Thromboembolism		FP	HP:0002719	Recurrent infections		FP
HP:0002094	Dyspnea		FP	HP:0000989	Pruritus	477322 18322771	TP
HP:0000969	Edema	17176413	TP	HP:0001635	Congestive heart failure		FP
HP:0100608	Metrorrhagia		FP	HP:0000964	Eczema		FP
HP:0001541	Ascites		FP	HP:0002090	Pneumonia		FP
HP:0000099	Glomerulonephritis		FP	HP:0004325	Decreased body weight		FP
HP:0002202	Pleural effusion	1926037	TP	HP:0004722	Thickening of the glomerular basement membrane		FP
HP:0003641	Hemoglobinuria		FP	HP:0001903	Anemia		FP
HP:0100526	Neoplasm of the lungs		FP	HP:0010783	Erythema		FP
HP:0002586	Peritonitis	8244870 1327358	TP	HP:0002527	Falls		FP
HP:0001596	Alopecia		FP	HP:0003712	Muscle hypertrophy		FP
HP:0100760	Clubbing of toes		FP	HP:0001289	Confusion		FP
HP:0010310	Chylothorax		FP	HP:0002108	Spontaneous pneumothorax		FP
HP:0000789	Infertility		FP	HP:0100845	Anaphylactic shock		FP
HP:0002013	Vomiting		FP	HP:0100749	Chest pain	12822426	TP
HP:0000505	Visual impairment		FP	HP:0008222	Female infertility		FP
HP:0200036	Skin nodule	19127968	TP	HP:0006530	Interstitial pulmonary disease		FP
HP:0100770	Hyperperistalsis		FP	HP:0002039	Anorexia		FP
HP:0010444	Pulmonary insufficiency		FP	HP:0001279	Syncope		FP
HP:0001945	Fever	9022330	TP	HP:0001909	Leukemia		FP
HP:0000793	Membranoproliferative glomerulonephritis		FP	HP:0001254	Lethargy		FP
HP:0100725	Lichenification		FP	HP:0002113	Pulmonary infiltrates	12693088	TP
HP:0001257	Spasticity		FP	HP:0000093	Proteinuria		FP
HP:0003256	Abnormality of the coagulation cascade		FP	HP:0004420	Arterial thrombosis		FP
HP:0003573	Increased total bilirubin		FP	HP:0005521	Disseminated intravascular coagulation		FP
HP:0002105	Hemoptysis	2643558	FN	HP:0000520	Proptosis	10094355	FN
HP:0002955	Granulomatosis	3616266	FN	HP:0002716	Lymphadenopathy	22915604	FN
HP:0100534	Episcleritis	7739879	FN	HP:0002840	Lymphadenitis	3626438	FN
HP:0003095	Septic arthritis	3435572 3626438	FN	HP:0100750	Atelectasis	12822426	FN
HP:0000651	Diplopia	998706	FN	HP:0007734	Enlarged lacrimal glands	17440285	FN
HP:0100540	Palpebral edema	20653124	FN	HP:0010605	Chalazion	11521439	FN
HP:0011921	Exudative pleural effusion	12822426	FN	HP:0007879	Allergic conjunctivitis	11055226	FN
HP:0003212	Increased IgE level	9022330	FN	HP:0002875	Exertional dyspnea	9022330	FN
HP:0000508	Ptosis	12213168	FN				

Table S15. Overview of HPO annotations for **Opisthorchiasis** that were derived by concept recognition in PubMed using BioLark. There were 19 true positives, 21 false positives, and 14 false negatives.

ID	Name	pmid	status	ID	Name	pmid	status
HP:0002240	Hepatomegaly	4095605	TP	HP:0001082	Cholecystitis	4095605	TP
HP:0003765	Psoriasis	15452616 12660845	TP	HP:0001081	Cholelithiasis	16768350	TP
HP:0001880	Eosinophilia	21938537	TP	HP:0001396	Cholestasis	21938537	TP
HP:0000952	Jaundice	4012543	TP	HP:0002719	Recurrent infections		FP
HP:0001394	Cirrhosis		FP	HP:0002896	Neoplasm of the liver		FP
HP:0002321	Vertigo		FP	HP:0003326	Myalgia		FP
HP:0006560	Biliary hyperplasia	2772709	TP	HP:0002027	Abdominal pain	21938537	TP
HP:0002018	Nausea		FP	HP:0001733	Pancreatitis	19329217	TP
HP:0001945	Fever	4012543	TP	HP:0006562	Viral hepatitis		FP
HP:0001402	Hepatocellular carcinoma	21603286	TP	HP:0001324	Muscle weakness		FP
HP:0002039	Anorexia	6542384	TP	HP:0003365	Arthralgia of the hip		FP
HP:0100523	Liver abscess	2558417	TP	HP:0002017	Nausea and vomiting		FP
HP:0002588	Duodenal ulcer	-	TP	HP:0001408	Bile duct proliferation	2772709	TP
HP:0003573	Increased total bilirubin		FP	HP:0002329	Drowsiness		FP
HP:0002527	Falls		FP	HP:0000099	Glomerulonephritis		FP
HP:0002592	Gastric ulcer		FP	HP:0005609	Gallbladder dysfunction	6542384	TP
HP:0000737	Irritability		FP	HP:0005231	Chronic gastritis	15484977	TP
HP:0002024	Malabsorption	2727922	TP	HP:0003075	Hypoproteinemia		FP
HP:0002375	Hypokinesia		FP	HP:0011227	Elevated C-reactive protein level		FP
HP:0001406	Intrahepatic cholestasis		FP	HP:0003394	Muscle cramps		FP
HP:0002613	Biliary cirrhosis	19329217	FN	HP:0001824	Weight loss	1544352 6542384	FN
HP:0005230	Biliary tract obstruction	6542384	FN	HP:0001407	Hepatic cysts	1803102 18725803	FN
HP:0003155	Elevated alkaline phosphatase	14574844 4095605	FN	HP:0002605	Hepatic necrosis	14574844	FN
HP:0100724	Hypercoagulability	19202620 21932543	FN	HP:0006559	Hepatic calcification	18725803	FN
HP:0011900	Hypofibrinogenemia	21932543	FN	HP:0006580	Portal fibrosis	6542384	FN
HP:0002630	Fat malabsorption	20873180	FN	HP:0003073	Hypoalbuminemia	4095605	FN
HP:0002904	Hyperbilirubinemia	4095605	FN	HP:0002910	Elevated hepatic transaminases	4095605	FN

| |

Table S16. Overview of HPO annotations for **Croup** that were derived by concept recognition in PubMed using BioLark. There were 13 true positives, 13 false positives, and 24 false negatives.

ID	Name	pmid	status	ID	Name	pmid	status
HP:0010307	Stridor	9451322	TP	HP:0002781	Upper airway obstruction	9445317	TP
HP:0011950	Bronchiolitis		FP	HP:0002099	Asthma		FP
HP:0005348	Inspiratory stridor	18646444	TP	HP:0002098	Respiratory distress	6386967	TP
HP:0002090	Pneumonia		FP	HP:0011947	Respiratory tract infection	21249651	TP
HP:0002783	Recurrent lower respiratory tract infections		FP	HP:0002835	Aspiration		FP
HP:0001609	Hoarse voice	18646444	TP	HP:0001607	Subglottic stenosis	21493242	TP
HP:0005945	Laryngeal obstruction	15523420	TP	HP:0001945	Fever	10910624	TP
HP:0000961	Cyanosis	6386967	TP	HP:0000969	Edema		FP
HP:0001601	Laryngomalacia		FP	HP:0002527	Falls		FP
HP:0002788	Recurrent upper respiratory tract infections		FP	HP:0011110	Tonsillitis		FP
HP:0002020	Gastroesophageal reflux		FP	HP:0002093	Respiratory insufficiency	15523420	TP
HP:0001602	Laryngeal stenosis	22995201	TP	HP:0002094	Dyspnea	8628614	TP
HP:0001613	Hoarse voice (caused by tumor impingement)		FP	HP:0001606	Vocal cord paralysis (caused by tumor impingement)		FP
HP:0004894	Laryngotracheal stenosis	3924864	FN	HP:0001618	Dysphonia	22433683	FN
HP:0100750	Atelectasis	19859734	FN	HP:0011948	Acute respiratory tract infection	18995152	FN
HP:0100598	Pulmonary edema	857236	FN	HP:0012027	Laryngeal edema	7800389	FN
HP:0002880	Respiratory difficulties	529358	FN	HP:0011134	Low-grade fever	8336098	FN
HP:0003212	Increased IgE level	6778038	FN	HP:0002777	Tracheal stenosis	22995201	FN
HP:0001944	Dehydration	8417425	FN	HP:0002013	Vomiting	9990833	FN
HP:0000737	Irritability	8114457	FN	HP:0100806	Sepsis	11510049	FN
HP:0005951	Progressive inspiratory stridor	6386967	FN	HP:0008755	Laryngotracheomalacia	16363272	FN
HP:0003237	Increased IgG level	6778038	FN	HP:0002791	Hypoventilation	16647977	FN
HP:0000713	Agitation	-	FN	HP:0004429	Recurrent viral infections	2117137	FN
HP:0002870	Obstructive sleep apnea	6379587	FN	HP:0010783	Erythema	14723257	FN
HP:0004890	Elevated pulmonary artery pressure	16647977	FN	HP:0002017	Nausea and vomiting	10065566	FN

Table S17. Overview of HPO annotations for **Ethmoid Sinusitis** that were derived by concept recognition in PubMed using BioLark. There were 5 true positives, 13 false positives, and 30 false negatives.

ID	Name	pmid	status	ID	Name	pmid	status
HP:0000246	Sinusitis	7588871	TP	HP:0011109	Chronic sinusitis		FP
HP:0000255	Acute sinusitis		FP	HP:0100582	Nasal polyposis	16216171	TP
HP:0100658	Cellulitis		FP	HP:0011108	Recurrent sinusitis		FP
HP:0001742	Nasal obstruction	16496109	TP	HP:0000520	Proptosis		FP
HP:0002331	Headache (with pheochromocytoma)		FP	HP:0000572	Visual loss		FP
HP:0002099	Asthma		FP	HP:0001287	Meningitis	9072242	TP
HP:0000718	Aggressive behavior		FP	HP:0100699	Scarring		FP
HP:0002090	Pneumonia		FP	HP:0100653	Optic neuritis		FP
HP:0000245	Abnormality of the sinuses		FP	HP:0000486	Strabismus	12792325	TP
HP:0002754	Osteomyelitis	20069309	FN	HP:0001945	Fever	18431903	FN
HP:0002719	Recurrent infections	9230316	FN	HP:0000651	Diplopia	16238043	FN
HP:0001880	Eosinophilia	8335853	FN	HP:0000622	Blurred vision	9037991	FN
HP:0000602	Ophthalmoplegia	1845269	FN	HP:0000421	Epistaxis	1391808	FN
HP:0002788	Recurrent upper respiratory tract infections	11385344	FN	HP:0005305	Cerebral venous thrombosis	8750066	FN
HP:0100806	Sepsis	10699248	FN	HP:0002315	Headache	19076651	FN
HP:0002257	Chronic rhinitis	19452706	FN	HP:0100539	Periorbital edema	8830571	FN
HP:0004409	Hyposmia	17685054	FN	HP:0000603	Central scotoma	20727299	FN
HP:0000579	Nasolacrimal duct obstruction	20639782	FN	HP:0000458	Anosmia	8758625	FN
HP:0011134	Low-grade fever	12652233	FN	HP:0009926	Increased lacrimation	20639782	FN
HP:0000575	Scotoma	9695165	FN	HP:0001085	Papilledema	11713715	FN
HP:0007686	Abnormal pupillary function	9037991	FN	HP:0001123	Visual field defect	9695165	FN
HP:0000508	Ptosis	19930782	FN	HP:0100660	Dyskinesia	19953662	FN
HP:0010783	Erythema	8944354	FN	HP:0002013	Vomiting	7772962	FN
HP:0000737	Irritability	11902076	FN	HP:0002360	Sleep disturbance	8515694	FN

| |

Table S18. Overview of HPO annotations for **Laryngeal Tuberculosis** that were derived by concept recognition in PubMed using BioLark. There were 10 true positives, 2 false positives, and 9 false negatives.

ID	Name	pmid	status	ID	Name	pmid	status
HP:0001609	Hoarse voice	19720251	TP	HP:0001618	Dysphonia	11715262	TP
HP:0002015	Dysphagia	9580143	TP	HP:0001613	Hoarse voice (caused by tumor impingement)		FP
HP:0011110	Tonsillitis	18634293	TP	HP:0010307	Stridor	8445701	TP
HP:0002716	Lymphadenopathy	16360822	TP	HP:0001945	Fever	15455624	TP
HP:0001824	Weight loss	22755382	TP	HP:0002840	Lymphadenitis	7681653 14567053	TP
HP:0002721	Immunodeficiency		FP	HP:0002955	Granulomatosis	18538743	TP
HP:0011850	Parotitis	19656502 16358915 9627234	FN	HP:0000975	Hyperhidrosis	22755382	FN
HP:0002094	Dyspnea	17633676	FN	HP:0011134	Low-grade fever	19621599	FN
HP:0002113	Pulmonary infiltrates	11347458	FN	HP:0006511	Laryngeal stridor	7803014	FN
HP:0012027	Laryngeal edema	19621599	FN	HP:0002781	Upper airway obstruction	8445701	FN
HP:0002039	Anorexia	15455624	FN				

| |

Table S19. Overview of HPO annotations for **Acromegaly** that were derived by concept recognition in PubMed using BioLark. There were 21 true positives, 71 false positives, and 23 false negatives.

ID	Name	pmid	status	ID	Name	pmid	status
HP:0000845	Growth hormone excess		FP	HP:0002893	Pituitary adenoma		FP
HP:0011750	Neoplasm of the anterior pituitary		FP	HP:0006767	Pituitary prolactin cell adenoma		FP
HP:0000870	Prolactin excess		FP	HP:0000822	Hypertension		FP
HP:0000819	Diabetes mellitus		FP	HP:0000855	Insulin resistance	18393170	TP
HP:0100646	Thyroiditis		FP	HP:0000833	Glucose intolerance	18578866	TP
HP:0002331	Headache (with pheochromocytoma)		FP	HP:0000824	Growth hormone deficiency		FP
HP:0002527	Falls		FP	HP:0001638	Cardiomyopathy		FP
HP:0001578	Hypercortisolism		FP	HP:0001733	Pancreatitis		FP
HP:0010735	Polyostotic fibrous dysplasia		FP	HP:0100570	Carcinoid		FP
HP:0001943	Hypoglycemia		FP	HP:0000135	Hypogonadism		FP
HP:0010535	Sleep apnea	18578866	TP	HP:0001712	Left ventricular hypertrophy		FP
HP:0001640	Cardiomegaly	18578866	TP	HP:0100568	Neoplasm of the endocrine system		FP
HP:0000836	Hyperthyroidism		FP	HP:0000975	Hyperhidrosis		FP
HP:0001635	Congestive heart failure		FP	HP:0000873	Diabetes insipidus		FP
HP:0001123	Visual field defect	23337021	TP	HP:0000821	Hypothyroidism		FP
HP:0000141	Amenorrhea	18578866	TP	HP:0001297	Stroke		FP
HP:0000303	Mandibular prognathia	10196815	TP	HP:0003040	Arthropathy		FP
HP:0001513	Obesity		FP	HP:0000718	Aggressive behavior		FP
HP:0007354	Amyotrophic lateral sclerosis		FP	HP:0000871	Panhypopituitarism		FP
HP:0000853	Goiter	18578866	TP	HP:0000839	Pituitary dwarfism		FP
HP:0003074	Hyperglycemia		FP	HP:0002870	Obstructive sleep apnea	18578866	TP
HP:0011761	Pituitary null cell adenoma		FP	HP:0000939	Osteoporosis		FP
HP:0003365	Arthralgia of the hip	18578866	TP	HP:0003003	Colon cancer		FP
HP:0000842	Hyperinsulinemia	1806481	TP	HP:0002829	Arthralgia		FP
HP:0001324	Muscle weakness		FP	HP:0004322	Short stature		FP
HP:0000158	Macroglossia	18578866	TP	HP:0100829	Galactorrhoea	11352287	TP
HP:0001000	Abnormality of skin pigmentation		FP	HP:0002014	Diarrhea		FP
HP:0001952	Abnormal glucose tolerance		FP	HP:0003510	Severe short stature		FP
HP:0000831	Insulin-resistant diabetes mellitus	18578866	TP	HP:0001677	Coronary artery disease		FP
HP:0100774	Hyperostosis	18578866	TP	HP:0003005	Ganglioneuroma		FP
HP:0000098	Tall stature	21158216	TP	HP:0011675	Arrhythmia		FP
HP:0002666	Pheochromocytoma		FP	HP:0002039	Anorexia		FP
HP:0002910	Elevated hepatic transaminases		FP	HP:0002858	Meningioma		FP
HP:0001644	Dilated cardiomyopathy		FP	HP:0001627	Abnormality of the heart		FP
HP:0002690	Large sella turcica	9474613	TP	HP:0000505	Visual impairment		FP
HP:0010541	Cutis gyrata of scalp	18211488	TP	HP:0000956	Acanthosis nigricans	7951506	TP
HP:0002781	Upper airway obstruction	18578866	TP	HP:0001654	Abnormality of the heart valves		FP
HP:0002758	Osteoarthritis		FP	HP:0003774	End stage renal disease		FP
HP:0002119	Ventriculomegaly		FP	HP:0008291	Pituitary corticotropic cell adenoma		FP
HP:0011760	Pituitary growth hormone cell adenoma		FP	HP:0000147	Polycystic ovaries	17651451	TP
HP:0000103	Polyuria		FP	HP:0100651	Type I diabetes mellitus		FP
HP:0005978	Type II diabetes mellitus		FP	HP:0002684	Thickened calvaria		FP
HP:0009800	Maternal diabetes		FP	HP:0000140	Abnormality of the menstrual cycle		FP
HP:0000823	Delayed puberty		FP	HP:0003162	Fasting hypoglycemia		FP
HP:0003351	Decreased circulating renin level		FP	HP:0011762	Pituitary thyrotropic cell adenoma		FP
HP:0002737	Thick skull base		FP	HP:0002681	Deformed sella turcica		FP

continued on the next page

Table S19. Acromegaly – continued

ID	Name	pmid	status	ID	Name	pmid	status
HP:0002007	Frontal bossing	18578866	FN	HP:0000280	Coarse facial features	23329711	FN
HP:0010609	Skin tags	18578866	FN	HP:0001670	Asymmetric septal hypertrophy	154293	FN
HP:0001176	Large hands	18578866	FN	HP:0000858	Menstrual irregularities	17651451	FN
HP:0004416	Precocious atherosclerosis	9389993	FN	HP:0005987	Multinodular goiter	18578866	FN
HP:0005994	Nodular goiter	18578866	FN	HP:0002150	Hypercalciuria	1668402	FN
HP:0001685	Myocardial fibrosis	1395769	FN	HP:0008843	Hip osteoarthritis	21131647	FN
HP:0001007	Hirsutism	10443669	FN	HP:0003416	Spinal canal stenosis	6664455 7919651	FN
HP:0000689	Dental malocclusion	10196815	FN	HP:0001548	Overgrowth	6805079	FN
HP:0001639	Hypertrophic cardiomyopathy	20834198 9711886	FN	HP:0001653	Mitral regurgitation	16580860	FN
HP:0001081	Cholelithiasis	8432484	FN	HP:0001714	Ventricular hypertrophy	18578866	FN
HP:0001072	Thickened skin	18578866	FN	HP:0004308	Ventricular arrhythmia	18578866	FN
HP:0004438	Hyperostosis frontalis interna	3731577	FN				

Table S20. Overview of HPO annotations for **Primary Hyperparathyroidism** that were derived by concept recognition in PubMed using BioLark. There were 14 true positives, 22 false positives, and 19 false negatives.

ID	Name	pmid	status	ID	Name	pmid	status
HP:0008200	Primary hyperparathyroidism	22173046	TP	HP:0002897	Parathyroid adenoma		FP
HP:0003072	Hypercalcemia	22271812	TP	HP:0006780	Parathyroid carcinoma		FP
HP:0000787	Nephrolithiasis	22173046	TP	HP:0100646	Thyroiditis		FP
HP:0100568	Neoplasm of the endocrine system		FP	HP:0000867	Secondary hyperparathyroidism		FP
HP:0002901	Hypocalcemia		FP	HP:0008208	Parathyroid hyperplasia	19679950	TP
HP:0000828	Abnormality of the parathyroid gland		FP	HP:0000843	Hyperparathyroidism		FP
HP:0011769	Ectopic parathyroid		FP	HP:0000939	Osteoporosis	18057667	TP
HP:0002757	Recurrent fractures	23098341	TP	HP:0011770	Tertiary hyperparathyroidism		FP
HP:0000820	Abnormality of the thyroid gland		FP	HP:0000938	Osteopenia	19685826 18057655	TP
HP:0000829	Hypoparathyroidism		FP	HP:0003165	Elevated circulating parathyroid hormone (PTH) level	23374741	TP
HP:0002150	Hypercalciuria	22584631	TP	HP:0000121	Nephrocalcinosis	23715355	TP
HP:0002653	Bone pain	17263969	TP	HP:0000822	Hypertension		FP
HP:0001324	Muscle weakness	22271812	TP	HP:0000853	Goiter		FP
HP:0002835	Aspiration		FP	HP:0002895	Papillary thyroid carcinoma		FP
HP:0005987	Multinodular goiter		FP	HP:0000103	Polyuria	20200146	TP
HP:0005897	Severe osteoporosis	23553864	TP	HP:0000836	Hyperthyroidism		FP
HP:0002527	Falls		FP	HP:0003774	End stage renal disease		FP
HP:0001733	Pancreatitis		FP	HP:0003127	Hypocalciuria		FP
HP:0002148	Hypophosphatemia	17201799	FN	HP:0003155	Elevated alkaline phosphatase	17370440	FN
HP:0002756	Pathologic fracture	19685826	FN	HP:0004934	Vascular calcification	23046088	FN
HP:0003326	Myalgia	21153954	FN	HP:0002019	Constipation	21723154	FN
HP:0002354	Memory impairment	17263969	FN	HP:0001735	Acute pancreatitis	18194938	FN
HP:0002039	Anorexia	19999395	FN	HP:0002018	Nausea	17263969	FN
HP:0000737	Irritability	19999395	FN	HP:0002027	Abdominal pain	17602056	FN
HP:0004349	Reduced bone mineral density	17602056	FN	HP:0001824	Weight loss	17263969	FN
HP:0000716	Depression	23374740	FN	HP:0001254	Lethargy	17602056	FN
HP:0000720	Mood swings	17263969	FN	HP:0002748	Rickets	19189688	FN
HP:0004724	Calcium nephrolithiasis	21183554	FN				

Table S21. Overview of HPO annotations for **Alcoholic Pancreatitis** that were derived by concept recognition in PubMed using BioLark. There were 18 true positives, 8 false positives, and 17 false negatives.

ID	Name	pmid	status	ID	Name	pmid	status
HP:0006280	Chronic pancreatitis		FP	HP:0001735	Acute pancreatitis	12828958	TP
HP:0005206	Pancreatic pseudocyst	17967181	TP	HP:0001733	Pancreatitis		FP
HP:0100732	Pancreatic fibrosis	12828957	TP	HP:0100027	Recurrent pancreatitis	18415757	TP
HP:0005213	Pancreatic calcification	13509579	TP	HP:0002027	Abdominal pain	12170706	TP
HP:0001081	Cholelithiasis		FP	HP:0002894	Neoplasm of the pancreas		FP
HP:0001738	Exocrine pancreatic insufficiency	12828957	TP	HP:0001394	Cirrhosis		FP
HP:0100844	Pancreatic fistula	22560825	TP	HP:0002239	Gastrointestinal hemorrhage	9445739	TP
HP:0000819	Diabetes mellitus	10430382	TP	HP:0002960	Autoimmunity		FP
HP:0002570	Steatorrhea	18985807	TP	HP:0006725	Pancreatic adenocarcinoma	20455050	TP
HP:0002202	Pleural effusion	17516324	TP	HP:0005236	Chronic calcifying pancreatitis	13509579	TP
HP:0001541	Ascites	17516324	TP	HP:0001396	Cholestasis	11393404	TP
HP:0000952	Jaundice	17198198	TP	HP:0004395	Malnutrition		FP
HP:0000488	Retinopathy	15803177	TP	HP:0002617	Aneurysm		FP
HP:0001737	Pancreatic cysts	20232071	FN	HP:0002248	Hematemesis	12353152	FN
HP:0002024	Malabsorption	9139143	FN	HP:0001409	Portal hypertension	16001677	FN
HP:0003077	Hyperlipidemia	22487474	FN	HP:0003418	Back pain	12170706	FN
HP:0002014	Diarrhea	9168660	FN	HP:0001824	Weight loss	15986640	FN
HP:0002586	Peritonitis	15500780	FN	HP:0001945	Fever	15273919	FN
HP:0100867	Duodenal stenosis	17198198 12383218	FN	HP:0002574	Episodic abdominal pain	19697839	FN
HP:0002013	Vomiting	15841034	FN	HP:0002573	Hematochezia	17148930	FN
HP:0003270	Abdominal distention	18516005	FN	HP:0001698	Pericardial effusion	9231991 12439127	FN
HP:0002249	Melena	17925742	FN				

Table S22. Overview of HPO annotations for **Angioedema** that were derived by concept recognition in PubMed using BioLark. There were 27 true positives, 24 false positives, and 47 false negatives.

ID	Name	pmid	status	ID	Name	pmid	status
HP:0100665	Angioedema	7083632	TP	HP:0001025	Urticaria	7083632	TP
HP:0012027	Laryngeal edema	18030852	TP	HP:0002027	Abdominal pain	11823949	TP
HP:0002099	Asthma		FP	HP:0100845	Anaphylactic shock	17244953	TP
HP:0004431	Complement deficiency		FP	HP:0010783	Erythema	-	TP
HP:0002781	Upper airway obstruction	8599504	TP	HP:0001880	Eosinophilia		FP
HP:0000822	Hypertension		FP	HP:0002574	Episodic abdominal pain	5511819	TP
HP:0000282	Facial edema	12139356	TP	HP:0002725	Systemic lupus erythematosus		FP
HP:0000964	Eczema		FP	HP:0002615	Hypotension	11842287	TP
HP:0005523	Lymphoproliferative disorder		FP	HP:0002013	Vomiting	17343080	TP
HP:0001541	Ascites	17285209	TP	HP:0001047	Atopic dermatitis		FP
HP:0001635	Congestive heart failure		FP	HP:0002014	Diarrhea	16464219	TP
HP:0002098	Respiratory distress	12829880	TP	HP:0002665	Lymphoma		FP
HP:0005945	Laryngeal obstruction	17487816	TP	HP:0002094	Dyspnea	17694700	TP
HP:0011458	Abdominal symptom	8438855	TP	HP:0100646	Thyroiditis		FP
HP:0100495	Mastocytosis		FP	HP:0002017	Nausea and vomiting		FP
HP:0011855	Pharyngeal edema	6649745	TP	HP:0005225	Intestinal edema	1215911	TP
HP:0003365	Arthralgia of the hip		FP	HP:0002015	Dysphagia	17296538	TP
HP:0002018	Nausea		FP	HP:0003193	Allergic rhinitis		FP
HP:0000099	Glomerulonephritis		FP	HP:0010307	Stridor	17487816	TP
HP:0003493	Antinuclear antibody positivity		FP	HP:0100539	Periorbital edema	21570492	TP
HP:0002527	Falls		FP	HP:0001369	Arthritis		FP
HP:0007430	Generalized edema	1611187	TP	HP:0005550	Chronic lymphatic leukemia		FP
HP:0002576	Intussusception	16464219	TP	HP:0001386	Joint swelling	1249347	TP
HP:0001279	Syncope		FP	HP:0002037	Inflammation of the large intestine	22408362	TP
HP:0004791	Esophageal ulceration		FP	HP:0006775	Multiple myeloma		FP
HP:0010742	Edema of the upper limbs	1650077	TP	HP:0010749	Blepharochalasis	18319025	FN
HP:0006511	Laryngeal stridor	2700663	FN	HP:0000158	Macroglossia	21495883	FN
HP:0002307	Drooling	18036423	FN	HP:0001609	Hoarse voice	3071076	FN
HP:0000988	Skin rash	1514010	FN	HP:0009763	Limb pain	9542615	FN
HP:0003270	Abdominal distention	11823949	FN	HP:0002321	Vertigo	22791189	FN
HP:0002202	Pleural effusion	11524698	FN	HP:0100749	Chest pain	14696809	FN
HP:0001945	Fever	20873964	FN	HP:0002960	Autoimmunity	17547847	FN
HP:0011848	Abdominal colic	10525217	FN	HP:0010808	Protruding tongue	8599504	FN
HP:0005339	Abnormality of complement system	589782	FN	HP:0003565	Elevated erythrocyte sedimentation rate	3823366	FN
HP:0001742	Nasal obstruction	2814292	FN	HP:0005521	Disseminated intravascular coagulation	341410	FN
HP:0004796	Gastrointestinal obstruction	23137231	FN	HP:0003496	Increased IgM level	9873168	FN
HP:0005348	Inspiratory stridor	-	FN	HP:0002880	Respiratory difficulties	10887769	FN
HP:0002789	Tachypnea	16230465	FN	HP:0011106	Hypovolemia	16271103	FN
HP:0000508	Ptosis	19298902	FN	HP:0100724	Hypercoagulability	9652897	FN
HP:0100598	Pulmonary edema	269002	FN	HP:0000967	Petechiae	6627625	FN
HP:0001618	Dysphonia	16267649	FN	HP:0001260	Dysarthria	8358121	FN

continued on the next page

Table S22. Angioedema – continued

ID	Name	pmid	status	ID	Name	pmid	status
HP:0002113	Pulmonary infiltrates	3264480	FN	HP:0000520	Proptosis	1911519	FN
HP:0002093	Respiratory insufficiency	1963718	FN	HP:0100326	Immunologic hypersensitivity	8959545	FN
HP:0000232	Everted lower lip vermilion	267704	FN	HP:0001225	Wrist swelling	11315937	FN
HP:0001041	Facial erythema	17505688	FN	HP:0100540	Palpebral edema	17694700	FN
HP:0001741	Phimosis	22560272	FN	HP:0010741	Edema of the lower limbs	15924048	FN
HP:0000157	Abnormality of the tongue	10619346	FN	HP:0004313	Hypogammaglobulinemia	3405564	FN
HP:0005214	Intestinal obstruction	17395288	FN	HP:0005268	Spontaneous abortion	-	FN
HP:0002890	Thyroid carcinoma	7170879	FN	HP:0001928	Abnormality of coagulation	-	FN

Table S23. Overview of HPO annotations for **Phototoxic Dermatitis** that were derived by concept recognition in PubMed using BioLark. There were 7 true positives, 10 false positives, and 1 false negatives.

ID	Name	pmid	status	ID	Name	pmid	status
HP:0000992	Cutaneous photosensitivity		FP	HP:0010783	Erythema		FP
HP:0000737	Irritability		FP	HP:0000964	Eczema	10438232	TP
HP:0007354	Amyotrophic lateral sclerosis		FP	HP:0001000	Abnormality of skin pigmentation	-	TP
HP:0000969	Edema	17223870	TP	HP:0001324	Muscle weakness		FP
HP:0007537	Severe photosensitivity	11868977	TP	HP:0008066	Abnormal blistering of the skin	17459294	TP
HP:0003765	Psoriasis		FP	HP:0000613	Photophobia		FP
HP:0001025	Urticaria		FP	HP:0002860	Squamous cell carcinoma		FP
HP:0002861	Malignant melanoma		FP	HP:0000989	Pruritus	19138025	TP
HP:0000953	Hyperpigmentation of the skin	19687425	TP	HP:0001806	Onycholysis	17688387	FN

Table S24. Overview of HPO annotations for **Dishidrotic Eczema** that were derived by concept recognition in PubMed using BioLark. There were 5 true positives, 2 false positives, and 5 false negatives.

ID	Name	pmid	status	ID	Name	pmid	status
HP:0000964	Eczema	11011918	TP	HP:0000975	Hyperhidrosis	1293189	TP
HP:0001047	Atopic dermatitis		FP	HP:0000989	Pruritus	22545332	TP
HP:0007410	Palmoplantar hyperhidrosis	8982415	TP	HP:0010783	Erythema	22691103	TP
HP:0003765	Psoriasis		FP	HP:0008391	Dystrophic fingernails	19076887	FN
HP:0007446	Palmoplantar blistering	8113043	FN	HP:0001065	Striae distensae	11395652	FN
HP:0003212	Increased IgE level	14616819	FN	HP:0000988	Skin rash	22738245	FN

Table S25. Overview of HPO annotations for **Viral Encephalitis** that were derived by concept recognition in PubMed using BioLark. There were 17 true positives, 32 false positives, and 32 false negatives.

ID	Name	pmid	status	ID	Name	pmid	status
HP:0002383	Encephalitis	-	TP	HP:0001298	Encephalopathy		FP
HP:0001287	Meningitis		FP	HP:0001250	Seizures		FP
HP:0001945	Fever	10349352 11858542 17326940	TP	HP:0002721	Immunodeficiency		FP
HP:0006846	Acute encephalopathy	11857527	TP	HP:0011096	Peripheral demyelination		FP
HP:0001259	Coma	11022140	TP	HP:0002373	Febrile seizures		FP
HP:0002181	Cerebral edema	15461026	TP	HP:0001974	Leukocytosis		FP
HP:0002331	Headache (with pheochromocytoma)		FP	HP:0003470	Paralysis		FP
HP:0011450	CNS infection		FP	HP:0001289	Confusion	23307455	TP
HP:0000726	Dementia		FP	HP:0001251	Ataxia	9471108	TP
HP:0003881	Humeral sclerosis		FP	HP:0001025	Urticaria		FP
HP:0002133	Status epilepticus	11022140	TP	HP:0002354	Memory impairment		FP
HP:0002171	Gliosis	7472530 12126146	TP	HP:0002960	Autoimmunity		FP
HP:0001336	Myoclonus		FP	HP:0002719	Recurrent infections		FP
HP:0006980	Leukoencephalopathy, progressive	19001657	TP	HP:0010280	Stomatitis		FP
HP:0002665	Lymphoma		FP	HP:0002329	Drowsiness	11858542	TP
HP:0100598	Pulmonary edema		FP	HP:0006965	Acute necrotizing encephalopathy	12116748 21801621	TP
HP:0001324	Muscle weakness		FP	HP:0002013	Vomiting	9471108	TP
HP:0002633	Vasculitis		FP	HP:0011947	Respiratory tract infection		FP
HP:0002093	Respiratory insufficiency		FP	HP:0001269	Hemiparesis	18045307 12353193	TP
HP:0003006	Neuroblastoma		FP	HP:0000639	Nystagmus	11857527 20544248	TP
HP:0001297	Stroke		FP	HP:0002045	Hypothermia		FP
HP:0002084	Encephalocele		FP	HP:0010543	Opsoclonus	9103875	TP
HP:0002301	Hemiplegia	16638508	TP	HP:0006530	Interstitial pulmonary disease		FP
HP:0002179	Opisthotonus		FP	HP:0001285	Spastic tetraparesis		FP
HP:0006957	Loss of ability to walk		FP	HP:0007307	Rapid neurologic deterioration	23107158	FN
HP:0002922	Increased CSF protein	11809148	FN	HP:0005318	Cerebral vasculitis	11118800	FN
HP:0002448	Progressive encephalopathy	8666375 14749962	FN	HP:0002446	Astrocytosis	22797933	FN
HP:0002300	Mutism	16847369 16776434	FN	HP:0002367	Visual hallucinations	12134688 12690279	FN
HP:0000741	Apathy	22790284	FN	HP:0002384	Focal seizures with impairment of consciousness or awareness	11022140	FN
HP:0000751	Personality changes	23307455	FN	HP:0001262	Somnolence	18021926	FN
HP:0002516	Increased intracranial pressure	17074607	FN	HP:0002072	Chorea	23307455	FN
HP:0007185	Loss of consciousness	10191896	FN	HP:0001254	Lethargy	23607233	FN
HP:0002059	Cerebral atrophy	15626538	FN	HP:0002902	Hyponatremia	23173742	FN
HP:0002353	EEG abnormality	16047296	FN	HP:0000713	Agitation	15730900 16283448 20549967	FN
HP:0002381	Aphasia	16909792	FN	HP:0010628	Facial palsy	11890853	FN

continued on the next page

Table S25. Viral Encephalitis – continued

ID	Name	pmid	status	ID	Name	pmid	status
HP:0100785	Insomnia	22971937 11706967 7555630 14679780	FN	HP:0006824	Cranial nerve paralysis	17116698	FN
HP:0002197	Generalized seizures	11022140	FN	HP:0001337	Tremor	15077022	FN
HP:0004305	Involuntary movements	7702698	FN	HP:0002921	Abnormality of the cerebrospinal fluid	-	FN
HP:0000737	Irritability	22357720	FN	HP:0001266	Choreoathetosis	10513697	FN
HP:0002069	Generalized tonic-clonic seizures	-	FN	HP:0002315	Headache	12757229	FN
HP:0008765	Auditory hallucinations	16047296	FN				

| | |

Table S26. Overview of HPO annotations for **Isaacs Syndrome** that were derived by concept recognition in PubMed using BioLark. There were 17 true positives, 9 false positives, and 15 false negatives.

ID	Name	pmid	status	ID	Name	pmid	status
HP:0002411	Myokymia	15257376	TP	HP:0003473	Fatigable weakness		FP
HP:0003394	Muscle cramps	15257376	TP	HP:0003552	Muscle stiffness	16770779	TP
HP:0100522	Thymoma	20420181	TP	HP:0002960	Autoimmunity	18801496	TP
HP:0002380	Fasciculations	16770779	TP	HP:0000651	Diplopia	17633107	TP
HP:0000975	Hyperhidrosis	15257376	TP	HP:0002131	Episodic ataxia		FP
HP:0002383	Encephalitis		FP	HP:0001250	Seizures		FP
HP:0009830	Peripheral neuropathy	17114847	TP	HP:0002486	Myotonia	12691809	TP
HP:0003401	Paresthesia	18801496	TP	HP:0001251	Ataxia		FP
HP:0100785	Insomnia	17114847	TP	HP:0002459	Dysautonomia		FP
HP:0000577	Exotropia	18607604	TP	HP:0003712	Muscle hypertrophy	20382536	TP
HP:0000486	Strabismus	18377936	TP	HP:0001260	Dysarthria	11360270	TP
HP:0003470	Paralysis		FP	HP:0001289	Confusion		FP
HP:0008978	Necrotizing myopathy		FP	HP:0001371	Flexion contracture	17048446	TP
HP:0002063	Rigidity	21576838	FN	HP:0001324	Muscle weakness	10768605	FN
HP:0002355	Difficulty walking	17048446	FN	HP:0000508	Ptosis	23337349	FN
HP:0002019	Constipation	15753614	FN	HP:0000317	Facial myokymia	12766989	FN
HP:0008981	Calf muscle hypertrophy	16607862	FN	HP:0010546	Muscle fibrillation	19679588	FN
HP:0009473	Joint contracture of the hand	17048446	FN	HP:0000565	Esotropia	17204915	FN
HP:0009763	Limb pain	16934467	FN	HP:0001311	Neurophysiological abnormality	16570308	FN
HP:0010628	Facial palsy	17114847	FN	HP:0002015	Dysphagia	17486731	FN
HP:0000737	Irritability	17114847	FN				

Table S27. Overview of HPO annotations for **Tibial Neuropathy** that were derived by concept recognition in PubMed using BioLark. There were 5 true positives, 3 false positives, and 5 false negatives.

ID	Name	pmid	status	ID	Name	pmid	status
HP:0003450	Axonal regeneration	18078754	TP	HP:0000763	Sensory neuropathy	-	TP
HP:0003202	Amyotrophy	20483119	TP	HP:0001324	Muscle weakness	-	TP
HP:0011096	Peripheral demyelination		FP	HP:0009831	Mononeuropathy	-	TP
HP:0002617	Aneurysm		FP	HP:0100537	Fasciitis		FP
HP:0003470	Paralysis	21284369	FN	HP:0100963	Hyperesthesia	7794070	FN
HP:0000762	Decreased nerve conduction velocity	21284369	FN	HP:0003401	Paresthesia	21600444	FN
HP:0001288	Gait disturbance	3970662	FN				

Table S28. Overview of HPO annotations for **Adult T-Cell Leukemia Lymphoma** that were derived by concept recognition in PubMed using BioLark. There were 13 true positives, 46 false positives, and 23 false negatives.

ID	Name	pmid	status	ID	Name	pmid	status
HP:0005517	T-cell lymphoma/leukemia	10397475	TP	HP:0001909	Leukemia		FP
HP:0006721	Acute lymphatic leukemia		FP	HP:0002665	Lymphoma		FP
HP:0002196	Myelopathy		FP	HP:0002488	Acute leukemia		FP
HP:0000718	Aggressive behavior		FP	HP:0003072	Hypercalcemia	18042693	TP
HP:0005526	Lymphoid leukemia		FP	HP:0004808	Acute myeloid leukemia		FP
HP:0002716	Lymphadenopathy	11798657	TP	HP:0004332	Abnormality of lymphocytes		FP
HP:0002721	Immunodeficiency		FP	HP:0005523	Lymphoproliferative disorder		FP
HP:0004430	Severe combined immunodeficiency		FP	HP:0001433	Hepatosplenomegaly	19684985	TP
HP:0005550	Chronic lymphatic leukemia		FP	HP:0004377	Hematological neoplasm		FP
HP:0002843	Abnormality of T cells		FP	HP:0001945	Fever	11798657	TP
HP:0001744	Splenomegaly	10996836	TP	HP:0001974	Leukocytosis	1920841	TP
HP:0000554	Uveitis		FP	HP:0001875	Neutropenia		FP
HP:0001324	Muscle weakness		FP	HP:0007354	Amyotrophic lateral sclerosis		FP
HP:0005558	Chronic leukemia		FP	HP:0001873	Thrombocytopenia		FP
HP:0100827	Lymphocytosis	15353320	TP	HP:0002090	Pneumonia		FP
HP:0008940	Generalized lymphadenopathy	15353320	TP	HP:0002240	Hepatomegaly	10495418	TP
HP:0001903	Anemia		FP	HP:0002960	Autoimmunity		FP
HP:0002202	Pleural effusion		FP	HP:0009919	Retinoblastoma		FP
HP:0004836	Acute promyelocytic leukemia		FP	HP:0009824	Hypoplasia involving bones of the upper limbs		FP
HP:0002863	Myelodysplasia		FP	HP:0001251	Ataxia		FP
HP:0001009	Telangiectasia		FP	HP:0002719	Recurrent infections		FP
HP:0000964	Eczema		FP	HP:0002835	Aspiration		FP
HP:0006775	Multiple myeloma		FP	HP:0008069	Neoplasm of the skin		FP
HP:0005531	Biphenotypic acute leukaemia		FP	HP:0004845	Acute monocytic leukemia		FP
HP:0002094	Dyspnea		FP	HP:0005506	Chronic myelogenous leukemia		FP
HP:0010783	Erythema	1942589	TP	HP:0004820	Acute myelomonocytic leukemia		FP
HP:0000952	Jaundice	11798657	TP	HP:0001000	Abnormality of skin pigmentation		FP
HP:0005547	Myeloproliferative disorder		FP	HP:0001882	Leukopenia	2975453	TP
HP:0011945	Bronchiolitis obliterans organizing pneumonia		FP	HP:0011946	Bronchiolitis obliterans		FP
HP:0001888	Lymphopenia		FP	HP:0001019	Erythroderma	17938020	FN
HP:0001482	Subcutaneous nodules	9010100	FN	HP:0000988	Skin rash	18516870	FN
HP:0002113	Pulmonary infiltrates	1321303	FN	HP:0001698	Pericardial effusion	1658079	FN
HP:0003401	Paresthesia	18035189 1662570 12350404	FN	HP:0002797	Osteolysis	16093798	FN
HP:0001876	Pancytopenia	11798657	FN	HP:0001880	Eosinophilia	11798657	FN
HP:0006530	Interstitial pulmonary disease	8331846 22578413	FN	HP:0002039	Anorexia	3204683	FN
HP:0002014	Diarrhea	3204683	FN	HP:0100806	Sepsis	16093798	FN
HP:0001824	Weight loss	15148763 11257818	FN	HP:0010702	Hypergammaglobulinemia	3067902	FN
HP:0010628	Facial palsy	19684985	FN	HP:0002653	Bone pain	9643532	FN
HP:0002756	Pathologic fracture	12432996	FN	HP:0200023	Priapism	15537405 10220081	FN
HP:0002249	Melena	3204683	FN	HP:0011974	Myelofibrosis	14715100	FN

continued on the next page

Table S28. Adult T-Cell Leukemia Lymphoma – continued

ID	Name	pmid	status	ID	Name	pmid	status
HP:0000979	Purpura	17973821 23224072	FN	HP:0100828	Increase in T cell number	-	FN

| |

Table S29. Overview of HPO annotations for **Plasma Cell Leukemia** that were derived by concept recognition in PubMed using BioLark. There were 14 true positives, 15 false positives, and 10 false negatives.

ID	Name	pmid	status	ID	Name	pmid	status
HP:0006775	Multiple myeloma		FP	HP:0001909	Leukemia		FP
HP:0011857	Plasmacytoma	21229400 9750458	TP	HP:0000718	Aggressive behavior		FP
HP:0002665	Lymphoma		FP	HP:0001903	Anemia	7571482	TP
HP:0001873	Thrombocytopenia	20391976	TP	HP:0000083	Renal insufficiency	11166824	TP
HP:0002488	Acute leukemia		FP	HP:0003072	Hypercalcemia	11166824	TP
HP:0002835	Aspiration		FP	HP:0005550	Chronic lymphatic leukemia		FP
HP:0002653	Bone pain	20391976	TP	HP:0000093	Proteinuria	1770327	TP
HP:0001433	Hepatosplenomegaly	3116673	TP	HP:0005526	Lymphoid leukemia		FP
HP:0001635	Congestive heart failure		FP	HP:0006721	Acute lymphatic leukemia		FP
HP:0005508	Waldenstrom macroglobulinemia		FP	HP:0005523	Lymphoproliferative disorder		FP
HP:0010702	Hypergammaglobulinemia	8086511 1578643 16454587 9796403	TP	HP:0001974	Leukocytosis	1942529	TP
HP:0011034	Amyloidosis		FP	HP:0001324	Muscle weakness		FP
HP:0002202	Pleural effusion	15078773 823757	TP	HP:0009824	Hypoplasia involving bones of the upper limbs		FP
HP:0001744	Splenomegaly	11166824	TP	HP:0002716	Lymphadenopathy	3116673	TP
HP:0002240	Hepatomegaly	3116673	TP	HP:0100806	Sepsis	3920242	FN
HP:0001824	Weight loss	12185504 18854288	FN	HP:0002090	Pneumonia	2652343	FN
HP:0001945	Fever	16304856	FN	HP:0100827	Lymphocytosis	15938728 10774246	FN
HP:0011974	Myelofibrosis	403845	FN	HP:0001919	Acute renal failure	16844565	FN
HP:0002797	Osteolysis	17453381	FN	HP:0001875	Neutropenia	17675269	FN
HP:0001876	Pancytopenia	687831	FN				

Table S30. Overview of HPO annotations for **Meningioma** that were derived by concept recognition in PubMed using BioLark. There were 17 true positives, 73 false positives, and 47 false negatives.

ID	Name	pmid	status	ID	Name	pmid	status
HP:0002858	Meningioma	7079409	TP	HP:0100009	Intracranial meningioma	6805281	TP
HP:0009733	Glioma		FP	HP:0100008	Schwannoma		FP
HP:0001287	Meningitis		FP	HP:0009588	Vestibular Schwannoma		FP
HP:0001067	Neurofibromas		FP	HP:0100843	Glioblastoma		FP
HP:0100010	Spinal meningioma	18485685	TP	HP:0000718	Aggressive behavior		FP
HP:0002331	Headache (with pheochromocytoma)		FP	HP:0009592	Astrocytoma		FP
HP:0000969	Edema		FP	HP:0002893	Pituitary adenoma		FP
HP:0002181	Cerebral edema	7242894	TP	HP:0001250	Seizures		FP
HP:0002888	Ependymoma		FP	HP:0010302	Spinal cord tumor		FP
HP:0002885	Medulloblastoma		FP	HP:0000520	Proptosis		FP
HP:0000246	Sinusitis		FP	HP:0001269	Hemiparesis	12826353	TP
HP:0100006	Neoplasm of the central nervous system		FP	HP:0010762	Chordoma		FP
HP:0010797	Hemangioblastoma		FP	HP:0100774	Hyperostosis		FP
HP:0000572	Visual loss		FP	HP:0000365	Hearing impairment		FP
HP:0001324	Muscle weakness		FP	HP:0003002	Breast carcinoma		FP
HP:0000651	Diplopia	12826353	TP	HP:0010628	Facial palsy	22236763	TP
HP:0000505	Visual impairment		FP	HP:0000529	Progressive visual loss	12812948	TP
HP:0100661	Trigeminal neuralgia		FP	HP:0001123	Visual field defect		FP
HP:0009734	Optic glioma		FP	HP:0100026	Arteriovenous malformation		FP
HP:0000648	Optic atrophy		FP	HP:0004944	Cerebral aneurysm		FP
HP:0002617	Aneurysm		FP	HP:0002321	Vertigo	12826353	TP
HP:0002835	Aspiration		FP	HP:0010799	Pinealoma		FP
HP:0001085	Papilledema	11018836	TP	HP:0002668	Paraganglioma		FP
HP:0007807	Optic nerve compression	17019421	TP	HP:0011750	Neoplasm of the anterior pituitary		FP
HP:0001138	Optic neuropathy		FP	HP:0001048	Cavernous hemangioma		FP
HP:0000360	Tinnitus	12836076	TP	HP:0001362	Skull defect		FP
HP:0009792	Teratoma		FP	HP:0002138	Subarachnoid hemorrhage	1664596 1085038	TP
HP:0001028	Hemangioma		FP	HP:0009589	Bilateral vestibular Schwannoma		FP
HP:0000822	Hypertension		FP	HP:0200022	Choroid plexus papilloma		FP
HP:0006765	Chondrosarcoma		FP	HP:0009830	Peripheral neuropathy		FP
HP:0005584	Renal cell carcinoma		FP	HP:0001297	Stroke		FP
HP:0003881	Humeral sclerosis		FP	HP:0000458	Anosmia	21840726	TP
HP:0002170	Intracranial hemorrhage		FP	HP:0004947	Arteriovenous fistula		FP
HP:0001342	Cerebral hemorrhage		FP	HP:0000508	Ptosis	20148271	TP
HP:0010828	Hemifacial spasm	11346028	TP	HP:0100646	Thyroiditis		FP
HP:0002013	Vomiting		FP	HP:0100608	Metrorrhagia		FP
HP:0009797	Cholesteatoma		FP	HP:0100309	Subdural hemorrhage	1327621	TP
HP:0009824	Hypoplasia involving bones of the upper limbs		FP	HP:0000265	Mastoiditis		FP
HP:0100699	Scarring		FP	HP:0000602	Ophthalmoplegia		FP
HP:0100246	Osteoma		FP	HP:0000024	Prostatitis		FP
HP:0003001	Glomus jugular tumor		FP	HP:0000873	Diabetes insipidus		FP
HP:0100310	Epidural hemorrhage		FP	HP:0006880	Cerebellar hemangioblastoma		FP
HP:0001291	Abnormality of the cranial nerves		FP	HP:0011695	Cerebellar hemorrhage		FP
HP:0009718	Subependymal giant-cell astrocytoma		FP	HP:0100634	Neuroendocrine neoplasm		FP
HP:0100570	Carcinoid		FP	HP:0009590	Unilateral vestibular Schwannoma		FP
HP:0005758	Foramen magnum lesion	2711319	FN	HP:0002423	Long-tract signs	22430127	FN
HP:0000543	Optic disc pallor	17548990 18421411	FN	HP:0002512	Brain stem compression	20148271	FN

continued on the next page

Table S30. Meningioma – continued

ID	Name	pmid	status	ID	Name	pmid	status
HP:0004840	Hypochromic microcytic anemia	9452243 18987835	FN	HP:0001285	Spastic tetraparesis	3885068	FN
HP:0002078	Truncal ataxia	11561352	FN	HP:0009916	Anisocoria	16331147	FN
HP:0001133	Constricted visual fields	7474790	FN	HP:0010534	Transient global amnesia	3990976 21629022	FN
HP:0011349	Abducens palsy	8677007 835387	FN	HP:0004409	Hyposmia	6449061	FN
HP:0010523	Alexia	12826353	FN	HP:0000603	Central scotoma	16793428 7132192	FN
HP:0001293	Cranial nerve compression	16331147 16455530	FN	HP:0003487	Babinski sign	8677007	FN
HP:0002312	Clumsiness	3885068 12233093	FN	HP:0000871	Panhypopituitarism	8986165	FN
HP:0002317	Unsteady gait	7520545	FN	HP:0007340	Lower limb muscle weakness	16850962	FN
HP:0002357	Dysphasia	7335302	FN	HP:0001334	Communicating hydrocephalus	16776435 8205731	FN
HP:0010532	Paroxysmal vertigo	9560091	FN	HP:0002355	Difficulty walking	18548187	FN
HP:0002073	Progressive cerebellar ataxia	3704430	FN	HP:0001730	Progressive hearing impairment	16792549	FN
HP:0010524	Agnosia	7566392	FN	HP:0002066	Gait ataxia	12826353	FN
HP:0002273	Tetraparesis	16917615	FN	HP:0002367	Visual hallucinations	8729606	FN
HP:0002427	Motor aphasia	2325489	FN	HP:0002277	Horner syndrome	23230622	FN
HP:0002313	Spastic paraparesis	3249615	FN	HP:0002318	Cervical myelopathy	2253423	FN
HP:0001347	Hyperreflexia	18080720	FN	HP:0000751	Personality changes	11386827	FN
HP:0002301	Hemiplegia	17021731	FN	HP:0001260	Dysarthria	9736091	FN
HP:0002176	Spinal cord compression	12820045	FN	HP:0002353	EEG abnormality	3990976 426936 1189455	FN
HP:0002797	Osteolysis	22836795	FN	HP:0002381	Aphasia	12826353	FN
HP:0000639	Nystagmus	3871599	FN	HP:0007359	Focal seizures	-	FN
HP:0000975	Hyperhidrosis	6453259 12691806	FN	HP:0002197	Generalized seizures	16910461	FN
HP:0002354	Memory impairment	23359077	FN				

Table S31. Overview of HPO annotations for **Budd-Chiari Syndrome** that were derived by concept recognition in PubMed using BioLark. There were 21 true positives, 32 false positives, and 7 false negatives.

ID	Name	pmid	status	ID	Name	pmid	status
HP:0002639	Budd-Chiari syndrome	-	TP	HP:0001541	Ascites	4058415	TP
HP:0001409	Portal hypertension	18618075	TP	HP:0001394	Cirrhosis		FP
HP:0002240	Hepatomegaly	9462648	TP	HP:0100724	Hypercoagulability		FP
HP:0004936	Venous thrombosis		FP	HP:0005547	Myeloproliferative disorder		FP
HP:0001399	Hepatic failure		FP	HP:0002619	Varicose veins		FP
HP:0001901	Polycythemia		FP	HP:0002027	Abdominal pain	15695963	TP
HP:0001402	Hepatocellular carcinoma	9828703	TP	HP:0004818	Paroxysmal nocturnal hemoglobinuria		FP
HP:0002040	Esophageal varices	12107787	TP	HP:0006554	Acute hepatic failure	2069474	TP
HP:0002204	Pulmonary embolism		FP	HP:0001894	Thrombocytosis		FP
HP:0000969	Edema	4012606	TP	HP:0001744	Splenomegaly	12848215	TP
HP:0001410	Decreased liver function	-	TP	HP:0005543	Reduced protein C activity		FP
HP:0003270	Abdominal distention	16295731	TP	HP:0002308	Arnold-Chiari malformation		FP
HP:0002239	Gastrointestinal hemorrhage		FP	HP:0001433	Hepatosplenomegaly	1797212	TP
HP:0000952	Jaundice	20387679	TP	HP:0001298	Encephalopathy		FP
HP:0003396	Syringomyelia		FP	HP:0002910	Elevated hepatic transaminases	19560555	TP
HP:0003613	Antiphospholipid antibody positivity		FP	HP:0004420	Arterial thrombosis		FP
HP:0002605	Hepatic necrosis		FP	HP:0100243	Leiomyosarcoma		FP
HP:0004855	Reduced protein S activity		FP	HP:0004419	Recurrent thrombophlebitis	23373054	TP
HP:0001976	Reduced antithrombin III activity		FP	HP:0002625	Deep venous thrombosis		FP
HP:0001907	Thromboembolism		FP	HP:0010741	Edema of the lower limbs	4058415	TP
HP:0006580	Portal fibrosis	16534866	TP	HP:0100523	Liver abscess		FP
HP:0003256	Abnormality of the coagulation cascade		FP	HP:0100806	Sepsis		FP
HP:0001395	Hepatic fibrosis	8900915	TP	HP:0004448	Fulminant hepatic failure	9399778	TP
HP:0011874	Heparin-induced thrombocytopenia		FP	HP:0002633	Vasculitis		FP
HP:0001873	Thrombocytopenia		FP	HP:0002202	Pleural effusion		FP
HP:0002248	Hematemesis	15185028	TP	HP:0000718	Aggressive behavior		FP
HP:0005521	Disseminated intravascular coagulation		FP	HP:0003645	Prolonged partial thromboplastin time	12696825	FN
HP:0001971	Hypersplenism	16534866	FN	HP:0002480	Hepatic encephalopathy	15095845	FN
HP:0003073	Hypoalbuminemia	9519690 3197587	FN	HP:0006846	Acute encephalopathy	16318042 1008048 2162656	FN
HP:0003155	Elevated alkaline phosphatase	23143028 7724132	FN	HP:0002904	Hyperbilirubinemia	20112074 21074693 17763380	FN

Table S32. Overview of HPO annotations for **Celiac Disease** that were derived by concept recognition in PubMed using BioLark. There were 30 true positives, 63 false positives, and 51 false negatives.

ID	Name	pmid	status	ID	Name	pmid	status
HP:0002608	Celiac disease	-	TP	HP:0011473	Villous atrophy	21901262	TP
HP:0002024	Malabsorption	-	TP	HP:0002960	Autoimmunity	-	TP
HP:0002242	Abnormality of the intestine	-	TP	HP:0002570	Steatorrhea	6523389	TP
HP:0000964	Eczema		FP	HP:0002014	Diarrhea	-	TP
HP:0002720	IgA deficiency		FP	HP:0001903	Anemia		FP
HP:0002665	Lymphoma		FP	HP:0100280	Crohn's disease		FP
HP:0001824	Weight loss		FP	HP:0001738	Exocrine pancreatic insufficiency		FP
HP:0002037	Inflammation of the large intestine		FP	HP:0002028	Chronic diarrhea	-	TP
HP:0002027	Abdominal pain	-	TP	HP:0004395	Malnutrition		FP
HP:0001733	Pancreatitis		FP	HP:0100279	Ulcerative colitis		FP
HP:0000939	Osteoporosis	-	TP	HP:0000820	Abnormality of the thyroid gland		FP
HP:0100651	Type I diabetes mellitus		FP	HP:0004322	Short stature		FP
HP:0001891	Iron deficiency anemia	17325959	TP	HP:0000819	Diabetes mellitus		FP
HP:0000737	Irritability		FP	HP:0006280	Chronic pancreatitis		FP
HP:0100646	Thyroiditis		FP	HP:0000938	Osteopenia	11560797	TP
HP:0001508	Failure to thrive	-	TP	HP:0001984	Intolerance to protein		FP
HP:0002583	Colitis		FP	HP:0001510	Growth delay		FP
HP:0001251	Ataxia		FP	HP:0001250	Seizures		FP
HP:0003270	Abdominal distention	18060282	TP	HP:0003261	Increased IgA level		FP
HP:0002613	Biliary cirrhosis		FP	HP:0001548	Overgrowth		FP
HP:0100827	Lymphocytosis		FP	HP:0002019	Constipation		FP
HP:0002749	Osteomalacia	22593794	TP	HP:0001324	Muscle weakness		FP
HP:0009830	Peripheral neuropathy	-	TP	HP:0004789	Lactose intolerance	25072743	TP
HP:0000821	Hypothyroidism		FP	HP:0002514	Cerebral calcification	7558773 9822844	TP
HP:0001370	Rheumatoid arthritis		FP	HP:0000789	Infertility	-	TP
HP:0004315	IgG deficiency		FP	HP:0002630	Fat malabsorption	21447770	TP
HP:0008207	Primary adrenal insufficiency		FP	HP:0011107	Recurrent aphthous stomatitis	17919276	TP
HP:0002725	Systemic lupus erythematosus		FP	HP:0002721	Immunodeficiency		FP
HP:0000740	Anxiety (with pheochromocytoma)		FP	HP:0005268	Spontaneous abortion		FP
HP:0001513	Obesity		FP	HP:0100512	Vitamin D deficiency	23328299	TP
HP:0000867	Secondary hyperparathyroidism	20387675	TP	HP:0001369	Arthritis		FP
HP:0000872	Hashimoto thyroiditis		FP	HP:0003881	Humeral sclerosis		FP
HP:0003765	Psoriasis		FP	HP:0100647	Graves disease		FP
HP:0003159	Hyperoxaluria	835313	TP	HP:0003073	Hypoalbuminemia		FP
HP:0002835	Aspiration		FP	HP:0002527	Falls		FP
HP:0100753	Schizophrenia		FP	HP:0000823	Delayed puberty	8338991	TP
HP:0005229	Jejunoileal ulceration	16292096	TP	HP:0100327	Cow milk allergy		FP
HP:0005505	Refractory anemia	17704578	TP	HP:0002239	Gastrointestinal hemorrhage	8602182	TP
HP:0005202	Helicobacter pylori infection		FP	HP:0004332	Abnormality of lymphocytes		FP
HP:0007354	Amyotrophic lateral sclerosis		FP	HP:0005681	Juvenile rheumatoid arthritis		FP
HP:0004313	Hypogammaglobulinemia		FP	HP:0002757	Recurrent fractures	12867795	TP
HP:0004325	Decreased body weight		FP	HP:0000836	Hyperthyroidism		FP
HP:0003198	Myopathy	15389648	TP	HP:0001518	Small for gestational age		FP
HP:0000794	IgA nephropathy		FP	HP:0001945	Fever		FP

continued on the next page

Table S32. Celiac Disease – continued

ID	Name	pmid	status	ID	Name	pmid	status
HP:0002331	Headache (with pheochromocytoma)		FP	HP:0000980	Pallor		FP
HP:0001047	Atopic dermatitis		FP	HP:0000975	Hyperhidrosis		FP
HP:0000853	Goiter		FP	HP:0004385	Protracted diarrhea	7714682	FN
HP:0005265	Abnormality of the jejunum	6391982	FN	HP:0002041	Intractable diarrhea	24355936	FN
HP:0004840	Hypochromic microcytic anemia	8956178	FN	HP:0003146	Hypocholesterolemia	16945614	FN
HP:0002750	Delayed skeletal maturation	19201117	FN	HP:0007141	Sensorimotor neuropathy	9416814	FN
HP:0006297	Hypoplasia of dental enamel	10883318 20540401	FN	HP:0011892	Vitamin K deficiency	17768663	FN
HP:0002243	Protein-losing enteropathy	619623	FN	HP:0000869	Secondary amenorrhea	11150866	FN
HP:0002574	Episodic abdominal pain	4085741	FN	HP:0001972	Macrocytic anemia	20455043	FN
HP:0008151	Prolonged prothrombin time	16093880	FN	HP:0003075	Hypoproteinemia	14074696 15125378	FN
HP:0011856	Pica	2305699	FN	HP:0002073	Progressive cerebellar ataxia	19622110	FN
HP:0005897	Severe osteoporosis	21611842	FN	HP:0003701	Proximal muscle weakness	12439125	FN
HP:0002229	Alopecia areata	12603809	FN	HP:0002584	Intestinal bleeding	12664131	FN
HP:0100513	Vitamin E deficiency	16100995	FN	HP:0002672	Gastrointestinal carcinoma	19408741	FN
HP:0002917	Hypomagnesemia	16358091	FN	HP:0003477	Peripheral axonal neuropathy	16835287	FN
HP:0002748	Rickets	11132463	FN	HP:0002580	Volvulus	9587089	FN
HP:0002576	Intussusception	9464437 11003969	FN	HP:0004326	Cachexia	12368936	FN
HP:0002900	Hypokalemia	21525142	FN	HP:0003613	Antiphospholipid antibody positivity	21839587	FN
HP:0002459	Dysautonomia	16967315	FN	HP:0000141	Amenorrhea	20359791	FN
HP:0002901	Hypocalcemia	7088767 22593794	FN	HP:0001336	Myoclonus	3504245 16638509 22225790	FN
HP:0003401	Paresthesia	12771245	FN	HP:0011459	Esophageal carcinoma	8783767	FN
HP:0002240	Hepatomegaly	12685387	FN	HP:0002196	Myelopathy	12151653	FN
HP:0003493	Antinuclear antibody positivity	8293004	FN	HP:0001271	Polyneuropathy	15389648	FN
HP:0003326	Myalgia	22138844	FN	HP:0002829	Arthralgia	11907355	FN
HP:0001744	Splenomegaly	23619270 16175383	FN	HP:0000554	Uveitis	22408231	FN
HP:0000802	Impotence	20017709	FN	HP:0001397	Hepatic steatosis	23315648	FN
HP:0002244	Abnormality of the small intestine	-	FN	HP:0004349	Reduced bone mineral density	-	FN
HP:0004386	Gastrointestinal inflammation	-	FN	HP:0011458	Abdominal symptom	-	FN

Table S33. Overview of HPO annotations for **Acute Cholecystitis** that were derived by concept recognition in PubMed using BioLark. There were 9 true positives, 4 false positives, and 8 false negatives.

ID	Name	pmid	status	ID	Name	pmid	status
HP:0001082	Cholecystitis	-	TP	HP:0001081	Cholelithiasis	-	TP
HP:0100758	Gangrene	23132628	TP	HP:0002027	Abdominal pain	22872303	TP
HP:0001735	Acute pancreatitis		FP	HP:0001945	Fever	22872303	TP
HP:0000952	Jaundice	19691802	TP	HP:0002586	Peritonitis	17252294	TP
HP:0100806	Sepsis	17203529	TP	HP:0001733	Pancreatitis		FP
HP:0001974	Leukocytosis	23340953	TP	HP:0002835	Aspiration		FP
HP:0001513	Obesity		FP	HP:0002910	Elevated hepatic transaminases	19275859	FN
HP:0005609	Gallbladder dysfunction	17252300	FN	HP:0005230	Biliary tract obstruction	23271073	FN
HP:0003155	Elevated alkaline phosphatase	21876567	FN	HP:0002013	Vomiting	22153541	FN
HP:0001396	Cholestasis	17427067	FN	HP:0011227	Elevated C-reactive protein level	22872303	FN
HP:0002018	Nausea	25239990	FN				

Table S34. Overview of HPO annotations for **Duodenogastric Reflux** that were derived by concept recognition in PubMed using BioLark. There were 13 true positives, 8 false positives, and 4 false negatives.

ID	Name	pmid	status	ID	Name	pmid	status
HP:0002020	Gastroesophageal reflux	14518219	TP	HP:0100633	Esophagitis	19662586	TP
HP:0005263	Gastritis	-	TP	HP:0002592	Gastric ulcer	6690637	TP
HP:0002588	Duodenal ulcer	20458957	TP	HP:0100580	Barrett esophagus	19662586	TP
HP:0002835	Aspiration		FP	HP:0005231	Chronic gastritis	22289498	TP
HP:0001733	Pancreatitis		FP	HP:0004398	Peptic ulcer	11396533	TP
HP:0001081	Cholelithiasis		FP	HP:0005202	Helicobacter pylori infection		FP
HP:0002582	Chronic atrophic gastritis	1397852	TP	HP:0002013	Vomiting	17245178	TP
HP:0002017	Nausea and vomiting	-	TP	HP:0011459	Esophageal carcinoma	15102519	TP
HP:0004791	Esophageal ulceration		FP	HP:0001082	Cholecystitis		FP
HP:0002860	Squamous cell carcinoma		FP	HP:0002027	Abdominal pain	8674397	TP
HP:0002578	Gastroparesis		FP	HP:0006753	Neoplasm of the stomach	12429172	FN
HP:0003270	Abdominal distention	19099725	FN	HP:0100751	Esophageal neoplasm	-	FN
HP:0002018	Nausea	3863229	FN				

Table S35. Overview of HPO annotations for **Acute Necrotizing Pancreatitis** that were derived by concept recognition in PubMed using BioLark. There were 19 true positives, 17 false positives, and 21 false negatives.

ID	Name	pmid	status	ID	Name	pmid	status
HP:0001735	Acute pancreatitis	-	TP	HP:0001733	Pancreatitis		FP
HP:0000789	Infertility		FP	HP:0005206	Pancreatic pseudocyst	23268585	TP
HP:0100806	Sepsis	12748429	TP	HP:0006280	Chronic pancreatitis		FP
HP:0002027	Abdominal pain	11847949	TP	HP:0002586	Peritonitis		FP
HP:0001081	Cholelithiasis		FP	HP:0100844	Pancreatic fistula	17516324 23386143 21253397	TP
HP:0001541	Ascites	17516324	TP	HP:0000969	Edema		FP
HP:0100027	Recurrent pancreatitis		FP	HP:0000718	Aggressive behavior		FP
HP:0000083	Renal insufficiency	19262525	TP	HP:0100819	Intestinal fistula	23386143	TP
HP:0001945	Fever	23286256 10430383	TP	HP:0002098	Respiratory distress	17533079	TP
HP:0002155	Hypertriglyceridemia		FP	HP:0002093	Respiratory insufficiency		FP
HP:0001919	Acute renal failure	16282052	TP	HP:0002013	Vomiting	17219076 18716785 16106939	TP
HP:0002615	Hypotension	17106218	TP	HP:0002202	Pleural effusion	17516324	TP
HP:0000952	Jaundice		FP	HP:0001974	Leukocytosis	18981549	TP
HP:0002239	Gastrointestinal hemorrhage	16282052	TP	HP:0003270	Abdominal distention	15239271	TP
HP:0001738	Exocrine pancreatic insufficiency		FP	HP:0003077	Hyperlipidemia		FP
HP:0002090	Pneumonia		FP	HP:0004872	Incisional hernia		FP
HP:0000819	Diabetes mellitus	-	TP	HP:0001873	Thrombocytopenia		FP
HP:0001899	Increased hematocrit	12123089 18596637	TP	HP:0002625	Deep venous thrombosis		FP
HP:0002574	Episodic abdominal pain	19822503	FN	HP:0010444	Pulmonary insufficiency	20461065	FN
HP:0003073	Hypoalbuminemia	16282052	FN	HP:0011106	Hypovolemia	17163376	FN
HP:0002595	Ileus	16768334 19822503	FN	HP:0002901	Hypocalcemia	12608652 19696761 15007192 22049070 18405600	FN
HP:0005521	Disseminated intravascular coagulation	15998382 12001677 15782108	FN	HP:0002910	Elevated hepatic transaminases	22825263 19800984 9882816	FN
HP:0100598	Pulmonary edema	1101836	FN	HP:0001399	Hepatic failure	17444596	FN
HP:0003074	Hyperglycemia	15627657	FN	HP:0001298	Encephalopathy	18334145 18405600	FN
HP:0001824	Weight loss	19696761	FN	HP:0100592	Peritoneal abscess	11036297	FN
HP:0002570	Steatorrhea	16895491 14707732	FN	HP:0003075	Hypoproteinemia	20517265	FN
HP:0003418	Back pain	15911961	FN	HP:0002590	Paralytic ileus	18759203	FN
HP:0006846	Acute encephalopathy	17879709	FN	HP:0011227	Elevated C-reactive protein level	16145344	FN
HP:0100732	Pancreatic fibrosis	9445116	FN				

Table S36. Overview of HPO annotations for **Epididymitis** that were derived by concept recognition in PubMed using BioLark. There were 9 true positives, 20 false positives, and 2 false negatives.

**ID**	**Name**	**pmid**	**status**	**ID**	**Name**	**pmid**	**status**
HP:0000031	Epididymitis	22787516	TP	HP:0100796	Orchitis		FP
HP:0100813	Testicular torsion		FP	HP:0000024	Prostatitis		FP
HP:0000789	Infertility		FP	HP:0000010	Recurrent urinary tract infections		FP
HP:0001945	Fever	16294792	TP	HP:0000029	Testicular atrophy	3534264	TP
HP:0003251	Male infertility	23482360	TP	HP:0010788	Testicular neoplasm		FP
HP:0002835	Aspiration		FP	HP:0000969	Edema		FP
HP:0011962	Obstructive azoospermia	15064321	TP	HP:0002633	Vasculitis		FP
HP:0100790	Hernia		FP	HP:0000027	Azoospermia	2120839	TP
HP:0000798	Oligospermia	9542967	TP	HP:0000028	Cryptorchidism		FP
HP:0002960	Autoimmunity		FP	HP:0100518	Dysuria	22787516	TP
HP:0000979	Purpura		FP	HP:0010783	Erythema	3788880	TP
HP:0002721	Immunodeficiency		FP	HP:0100806	Sepsis		FP
HP:0002719	Recurrent infections		FP	HP:0200023	Priapism		FP
HP:0008222	Female infertility		FP	HP:0000796	Urethral obstruction		FP
HP:0000041	Chordee		FP	HP:0001974	Leukocytosis	18329081	FN
HP:0009714	Abnormality of the epididymis	618030 8520650	FN				

Table S37. Overview of HPO annotations for **Spermatic Cord Torsion** that were derived by concept recognition in PubMed using BioLark. There were 5 true positives, 15 false positives, and 8 false negatives.

ID	Name	pmid	status	ID	Name	pmid	status
HP:0100813	Testicular torsion	4087084	TP	HP:0000031	Epididymitis		FP
HP:0100796	Orchitis		FP	HP:0000028	Cryptorchidism		FP
HP:0000029	Testicular atrophy	4087084	TP	HP:0000789	Infertility		FP
HP:0000969	Edema		FP	HP:0100790	Hernia		FP
HP:0000023	Inguinal hernia		FP	HP:0100758	Gangrene	5007848	TP
HP:0003251	Male infertility	3090760	TP	HP:0002027	Abdominal pain	11019377 21903353	TP
HP:0010788	Testicular neoplasm		FP	HP:0010470	Supernumerary testes		FP
HP:0002017	Nausea and vomiting		FP	HP:0000979	Purpura		FP
HP:0000035	Abnormality of the testis		FP	HP:0008733	Dysplastic testes		FP
HP:0000053	Macroorchidism		FP	HP:0008720	Primary testicular failure		FP
HP:0000798	Oligospermia	3090760	FN	HP:0008669	Impaired spermatogenesis	3090760	FN
HP:0000802	Impotence	16138584	FN	HP:0010783	Erythema	10999695	FN
HP:0002013	Vomiting	21490540	FN	HP:0001945	Fever	11019377 21903353	FN
HP:0008734	Decreased testicular size	6776291	FN	HP:0000027	Azoospermia	16138584	FN

Table S38. Overview of HPO annotations for **Uterine Inversion** that were derived by concept recognition in PubMed using BioLark. There were 2 true positives, 7 false positives, and 4 false negatives.

**ID**	**Name**	**pmid**	**status**	**ID**	**Name**	**pmid**	**status**
HP:0100242	Sarcoma		FP	HP:0100718	Uterine rupture		FP
HP:0000139	Uterine prolapse	2647797	TP	HP:0000016	Urinary retention		FP
HP:0002027	Abdominal pain	17197359	TP	HP:0100519	Anuria		FP
HP:0006743	Embryonal rhabdomyosarcoma		FP	HP:0000718	Aggressive behavior		FP
HP:0000131	Uterine leiomyoma		FP	HP:0011891	Post-partum hemorrhage	12464994	FN
HP:0011106	Hypovolemia	15228824	FN	HP:0100608	Metrorrhagia	11848030	FN
HP:0001892	Abnormal bleeding	17578377	FN				

Table S39. Overview of HPO annotations for **Nephrogenic Diabetes Insipidus** that were derived by concept recognition in PubMed using BioLark. There were 11 true positives, 12 false positives, and 10 false negatives.

ID	Name	pmid	status	ID	Name	pmid	status
HP:0009806	Nephrogenic diabetes insipidus	-	TP	HP:0000103	Polyuria	22503803	TP
HP:0001959	Polydipsia	22503803	TP	HP:0001944	Dehydration	9831428	TP
HP:0003228	Hypernatremia	18715941	TP	HP:0000863	Central diabetes insipidus		FP
HP:0007302	Bipolar affective disorder		FP	HP:0000873	Diabetes insipidus		FP
HP:0003158	Hyposthenuria	16580609	TP	HP:0001508	Failure to thrive	16240160 15249704	TP
HP:0002900	Hypokalemia	12503936	TP	HP:0000126	Hydronephrosis	10332005	TP
HP:0000083	Renal insufficiency		FP	HP:0002902	Hyponatremia		FP
HP:0001249	Intellectual disability	16580609	TP	HP:0001947	Renal tubular acidosis		FP
HP:0001510	Growth delay	18584216	TP	HP:0010677	Enuresis nocturna		FP
HP:0003072	Hypercalcemia		FP	HP:0008341	Distal renal tubular acidosis		FP
HP:0001942	Metabolic acidosis		FP	HP:0001276	Hypertonia		FP
HP:0011037	Decreased urine output		FP	HP:0001263	Global developmental delay	15985744	FN
HP:0003774	End stage renal disease	18519085	FN	HP:0001945	Fever	10332005	FN
HP:0001250	Seizures	10332005	FN	HP:0002013	Vomiting	19703807 16240160	FN
HP:0000072	Hydroureter	10332005	FN	HP:0002514	Cerebral calcification	10332005	FN
HP:0000017	Nocturia	15249704 12784095	FN	HP:0001986	Hypertonic dehydration	10332005	FN
HP:0011106	Hypovolemia	18715941	FN				

Table S40. Overview of HPO annotations for **Focal Segmental Glomerulosclerosis.tab** that were derived by concept recognition in PubMed using BioLark. There were 13 true positives, 51 false positives, and 4 false negatives.

ID	Name	pmid	status	ID	Name	pmid	status
HP:0000097	Focal segmental glomerulosclerosis	-	TP	HP:0000093	Proteinuria	11863085	TP
HP:0000100	Nephrotic syndrome		FP	HP:0000096	Glomerulosclerosis		FP
HP:0000112	Nephropathy		FP	HP:0003774	End stage renal disease	12817066	TP
HP:0003881	Humeral sclerosis		FP	HP:0000099	Glomerulonephritis		FP
HP:0000822	Hypertension		FP	HP:0100820	Glomerulopathy	-	TP
HP:0000083	Renal insufficiency	11863085	TP	HP:0000794	IgA nephropathy		FP
HP:0000793	Membranoproliferative glomerulonephritis		FP	HP:0000123	Nephritis		FP
HP:0000092	Tubular atrophy		FP	HP:0005576	Tubulointerstitial fibrosis		FP
HP:0001967	Diffuse mesangial sclerosis		FP	HP:0000790	Hematuria		FP
HP:0003259	Increased creatinine	8706354	TP	HP:0100699	Scarring		FP
HP:0004737	global glomerulosclerosis	-	TP	HP:0002721	Immunodeficiency		FP
HP:0003077	Hyperlipidemia		FP	HP:0009741	Nephrosclerosis		FP
HP:0001513	Obesity		FP	HP:0003124	Hypercholesterolemia		FP
HP:0002907	Microhematuria	11863085	TP	HP:0000969	Edema		FP
HP:0003073	Hypoalbuminemia		FP	HP:0008653	Necrotizing glomerulonephritis		FP
HP:0002667	Nephroblastoma (Wilms tumor)		FP	HP:0003453	Antineutrophil antibody positivity		FP
HP:0004722	Thickening of the glomerular basement membrane	7301001	TP	HP:0002725	Systemic lupus erythematosus		FP
HP:0002633	Vasculitis		FP	HP:0000718	Aggressive behavior		FP
HP:0000819	Diabetes mellitus		FP	HP:0011034	Amyloidosis		FP
HP:0000859	Hyperaldosteronism		FP	HP:0002621	Atherosclerosis		FP
HP:0002586	Peritonitis		FP	HP:0007354	Amyotrophic lateral sclerosis		FP
HP:0002955	Granulomatosis		FP	HP:0000979	Purpura		FP
HP:0002157	Azotemia	-	TP	HP:0002155	Hypertriglyceridemia		FP
HP:0000037	Male pseudohermaphroditism		FP	HP:0007430	Generalized edema	17044480 20199191	TP
HP:0100608	Metrorrhagia		FP	HP:0003128	Lactic acidosis		FP
HP:0003613	Antiphospholipid antibody positivity		FP	HP:0003493	Antinuclear antibody positivity		FP
HP:0100778	Cryoglobulinemia		FP	HP:0001917	Renal amyloidosis		FP
HP:0001945	Fever		FP	HP:0003076	Glycosuria		FP
HP:0000855	Insulin resistance		FP	HP:0000028	Cryptorchidism		FP
HP:0003075	Hypoproteinemia	18789122	TP	HP:0003126	Low-molecular-weight proteinuria		FP
HP:0003138	Increased blood urea nitrogen (BUN)	-	TP	HP:0010741	Edema of the lower limbs		FP
HP:0008723	Gonadal dysgenesis with female appearance, male		FP	HP:0003206	Decreased activity of NADPH oxidase		FP
HP:0001966	Mesangial abnormality	9064487	FN	HP:0004421	Elevated systolic blood pressure	-	FN
HP:0100520	Oliguria	3987100 9532830	FN	HP:0010980	Hyperlipoproteinemia	8147062 9163848 3292816	FN

Table S41. Overview of HPO annotations for **Renal Artery Obstruction** that were derived by concept recognition in PubMed using BioLark. There were 13 true positives, 47 false positives, and 9 false negatives.

ID	Name	pmid	status	ID	Name	pmid	status
HP:0001920	Renal artery stenosis	-	TP	HP:0100817	Renovascular hypertension	-	TP
HP:0000822	Hypertension	-	TP	HP:0000083	Renal insufficiency		FP
HP:0002621	Atherosclerosis		FP	HP:0004420	Arterial thrombosis		FP
HP:0002617	Aneurysm		FP	HP:0001919	Acute renal failure	4006581	TP
HP:0000112	Nephropathy		FP	HP:0100545	Arterial stenosis		FP
HP:0003774	End stage renal disease	12823672	TP	HP:0003259	Increased creatinine	9527401	TP
HP:0004953	Abdominal aortic aneurysm		FP	HP:0000859	Hyperaldosteronism	19917331	TP
HP:0004950	Peripheral arterial disease		FP	HP:0001635	Congestive heart failure		FP
HP:0005313	Arterial fibromuscular dysplasia		FP	HP:0005294	Arterial dissection		FP
HP:0100519	Anuria	1259486	TP	HP:0001677	Coronary artery disease		FP
HP:0000093	Proteinuria	20665031	TP	HP:0004942	Aortic aneurysm		FP
HP:0100598	Pulmonary edema		FP	HP:0002527	Falls		FP
HP:0005315	Occlusive arterial disease		FP	HP:0000089	Renal hypoplasia		FP
HP:0000848	Increased circulating renin level	951017	TP	HP:0001067	Neurofibromas		FP
HP:0002157	Azotemia	19671391 8720081	TP	HP:0008682	Acute tubular necrosis		FP
HP:0001658	Myocardial infarction		FP	HP:0001650	Aortic valve stenosis		FP
HP:0000718	Aggressive behavior		FP	HP:0001297	Stroke		FP
HP:0000819	Diabetes mellitus		FP	HP:0002615	Hypotension		FP
HP:0000110	Renal dysplasia		FP	HP:0001680	Coarctation of aorta		FP
HP:0004974	Coarctation of abdominal aorta		FP	HP:0002666	Pheochromocytoma		FP
HP:0004713	Reversible renal failure	9527401	TP	HP:0004947	Arteriovenous fistula		FP
HP:0000790	Hematuria		FP	HP:0009741	Nephrosclerosis	8720083	TP
HP:0000099	Glomerulonephritis		FP	HP:0005145	Coronary artery stenosis		FP
HP:0002647	Aortic dissection		FP	HP:0002092	Pulmonary hypertension		FP
HP:0000969	Edema		FP	HP:0001907	Thromboembolism		FP
HP:0000126	Hydronephrosis		FP	HP:0006000	Ureteral obstruction		FP
HP:0000077	Abnormality of the kidney		FP	HP:0009726	Renal neoplasm		FP
HP:0004936	Venous thrombosis		FP	HP:0001681	Angina pectoris		FP
HP:0004929	Coronary atherosclerosis		FP	HP:0002641	Peripheral thrombosis		FP
HP:0100860	Inferior mesenteric artery aneurysm		FP	HP:0002204	Pulmonary embolism		FP
HP:0011741	Secondary hyperaldosteronism	6397520	FN	HP:0004421	Elevated systolic blood pressure	9507221	FN
HP:0001095	Hypertensive retinopathy	17051904	FN	HP:0100735	Hypertensive crisis	7603800	FN
HP:0000092	Tubular atrophy	8684534	FN	HP:0100520	Oliguria	12141408	FN
HP:0002900	Hypokalemia	19365633	FN	HP:0002902	Hyponatremia	15503172	FN
HP:0001578	Hypercortisolism	3725709	FN				

References

[1] Lynn Marie Schriml, Cesar Arze, Suvarna Nadendla, Yu-Wei Wayne Chang, Mark Mazaitis, Victor Felix, Gang Feng, and Warren Alden Kibbe. Disease Ontology: a backbone for disease semantic integration. *Nucleic Acids Res*, 40(Database issue):D940–D946, Jan 2012.
